# Detecting negative selection on recurrent mutations using gene genealogy

**DOI:** 10.1186/1471-2156-14-37

**Published:** 2013-05-07

**Authors:** Kiyoshi Ezawa, Giddy Landan, Dan Graur

**Affiliations:** 1 Department of Biology and Biochemistry, University of Houston, Houston, TX 77204-5001, USA; 2 Present address: Department of Bioscience and Bioinformatics, Kyushu Institute of Technology, Iizuka, Fukuoka 820-8502, Japan; 3 Present address: Institute of Genomic Microbiology, Heinrich-Heine University Düsseldorf, Universitätsstr. 1, Düsseldorf 40225, Germany

**Keywords:** Population genetics, Recurrent mutation, Negative selection, Deleterious mutation, Neutrality test

## Abstract

**Background:**

Whether or not a mutant allele in a population is under selection is an important issue in population genetics, and various neutrality tests have been invented so far to detect selection. However, detection of negative selection has been notoriously difficult, partly because negatively selected alleles are usually rare in the population and have little impact on either population dynamics or the shape of the gene genealogy. Recently, through studies of genetic disorders and genome-wide analyses, many structural variations were shown to occur recurrently in the population. Such “recurrent mutations” might be revealed as deleterious by exploiting the signal of negative selection in the gene genealogy enhanced by their recurrence.

**Results:**

Motivated by the above idea, we devised two new test statistics. One is the total number of mutants at a recurrently mutating locus among sampled sequences, which is tested conditionally on the number of forward mutations mapped on the sequence genealogy. The other is the size of the most common class of identical-by-descent mutants in the sample, again tested conditionally on the number of forward mutations mapped on the sequence genealogy. To examine the performance of these two tests, we simulated recurrently mutated loci each flanked by sites with neutral single nucleotide polymorphisms (SNPs), with no recombination. Using neutral recurrent mutations as null models, we attempted to detect deleterious recurrent mutations. Our analyses demonstrated high powers of our new tests under constant population size, as well as their moderate power to detect selection in expanding populations. We also devised a new maximum parsimony algorithm that, given the states of the sampled sequences at a recurrently mutating locus and an incompletely resolved genealogy, enumerates mutation histories with a minimum number of mutations while partially resolving genealogical relationships when necessary.

**Conclusions:**

With their considerably high powers to detect negative selection, our new neutrality tests may open new venues for dealing with the population genetics of recurrent mutations as well as help identifying some types of genetic disorders that may have escaped identification by currently existing methods.

## Background

Whether and how a mutant allele is selected is an important topic in population genetics, because it, along with the population size, demography, and the mode and tempo of mutation, crucially dictates the evolutionary fate of the mutant allele and/or the polymorphism pattern in the population (e.g., [1-4]). The type and intensity of selection also indicate the functional impact and the evolutionary history of a mutation and the locus that underwent it. A number of statistical tests have been developed to detect selection on mutant alleles (e.g., [5-12]). Most of them are based on the null-hypothesis that mutants are selectively neutral [13-17] and are called “neutrality tests.” These neutrality tests were successful to some degree in detecting balancing selection (e.g., [18-21]) and positive selection (e.g., [22-24]). Detection of negative selection, in contrast, has generally been unsuccessful, probably because of the weak signals displayed by deleterious mutants (e.g., [25] and references therein).

So far, development of tools for population genetics analyses has centered around the infinite-site model [26], which suitably describes single-nucleotide polymorphisms (SNPs), one of the commonest and most actively studied types of polymorphisms (e.g., [27-30]). Recent technological innovations, however, enabled the detection of another type of polymorphism, namely structural variations (SVs), including copy number variations (CNVs) (e.g., [31-35]). These studies have revealed that SVs are very common in eukaryotic genomes (e.g., [36-40]) including the human genome (e.g., [41-43]).

Some of the structurally variant mutations (SV mutations) associated with genomic diseases have long been known to recur in the human population (e.g., [44]). A recent genome-wide analysis suggested that such “recurrent mutations” are quite common among CNVs [45]. Recurrent mutations are also quite common among inversions, another well-known type of SV [46]. Assessing the selective force on each of such recurrent mutations is essential for estimating its evolutionary and/or medical impacts on the genome undergoing them. Positively selected (e.g., [47]) and selectively neutral (e.g., [48]) recurrent SV mutations drive genome evolution. Negatively selected recurrent SV mutations (reviewed e.g., in [44]), in contrast, will not substantially contribute to genomic differences between species. New identification of such deleterious recurrent mutations, however, may reveal some disorders whose genetic causes have so far remained elusive.

In this study, we attempt to detect negative selection on recurrent mutations, such as those generating SVs, by exploiting the gene genealogy of sampled sequences. Broadly speaking, our rationale is the following. Although the signal of negative selection on a single mutation event may be too weak to be detected, the synergistic effect of the signals from multiple mutation events of a specific type might become strong enough to enable detection. Therefore, if the genealogy of sampled sequences reveals recurrent mutation events, we may be able to detect negative selection on the mutants.

To validate this idea, we conducted an extensive computer simulation analysis. In the analysis, we first simulated recurrent mutations under different conditions in a population with a constant size of 10,000 and in populations that expanded from a bottleneck, all without recombination, using a coalescent simulator, *msms*[49]. Then we examined the ability of our new neutrality tests to correctly detect negative selection on recurrent mutations at each simulated locus. Our computer simulation analyses demonstrated that our new tests can correctly detect negative selection with high true-positive rates in constant-size population, and at moderate true-positive rates in expanding populations. This gives us some hope that our new neutrality tests may provide a useful means for real data scans to detect deleterious recurrent mutations, and also opens the possibility of further developing methods to address some outstanding issues, such as recombination and population substructure, that could not have been dealt with in this study.

Our new tests require an algorithm to map mutation events on a gene genealogy at the recurrently mutating locus. In this study, the genealogy is reconstructed from SNPs flanking (or residing within) the locus in question. For this purpose, we also developed a new maximum parsimony algorithm that overcomes a problem inherent in any traditional tree reconstruction algorithm coupled with any traditional parsimony-based mutation mapping algorithm, which is the tendency to overestimate the number of mutation events if the genealogy is inferred from SNPs (see *Methods*).

### Subjects of our new neutrality tests

Before going into the details of our methods, we would like to clarify what our new neutrality tests are intended for. In principle, our new tests are aimed at detecting negative selection on any type of recurrent mutations that satisfy the following two conditions: (i) the subject mutations in a test share some features clearly distinguishable from other mutations, especially neutral SNPs; and (ii) sequences with subject mutations can be sub-classified at least approximately into classes of shared origins (*i.e*., classes of identical-by-descent mutants) by some means, such as the genealogy of sequences, identifying characteristics, and/or exact locations.

Our original purpose was to judge whether recurrent mutations at each structurally variant (SV) locus are deleterious or not, using the sequence genealogy reconstructed with SNPs to identify the recurrent mutation events. SV mutations often have rates *θ*_*μ*_ (≡4*Nμ*) ∼ 1 (e.g., [44,45], where *N* is the (effective) population size and *μ* is the mutation rate per haploid locus per generation). Occasionally, *θ*_*μ*_>10 [45]. A second conceivable kind of subject is a set of mutations at a micro-satellite locus, which are known to occur at a very high rate, with *θ*_*μ*_ typically ranging from 1 to 100 (see e.g., [50]).

A third kind of subject would be a class of mutations that satisfy two conditions: (i) they occurred in a region, such as in a haplotype block, that consists of sites reasonably linked with one another; and (ii) they exhibit suspected signs of functional loss or impairment (e.g., insertions, frame-shifting indels, nonsense point mutations, and mutations on signals of splicing or gene expression) of a putative gene, such as the one predicted by a genome-wide annotation. The new tests on this class may be useful for inferring whether or not a putative gene is functional, especially when there are no other data to ascertain its purported functionality (see also *Discussion*).

Although the methods described in this paper are intended for applications to simple SV mutations, other potential subject mutations, such as the ones mentioned above, could also be examined by our new neutrality tests, as long as we can define appropriate null models.

## Methods

### Detecting negative selection on recurrent mutations using gene genealogy (I): Theoretical rationale and test statistics

**Figure 1:**
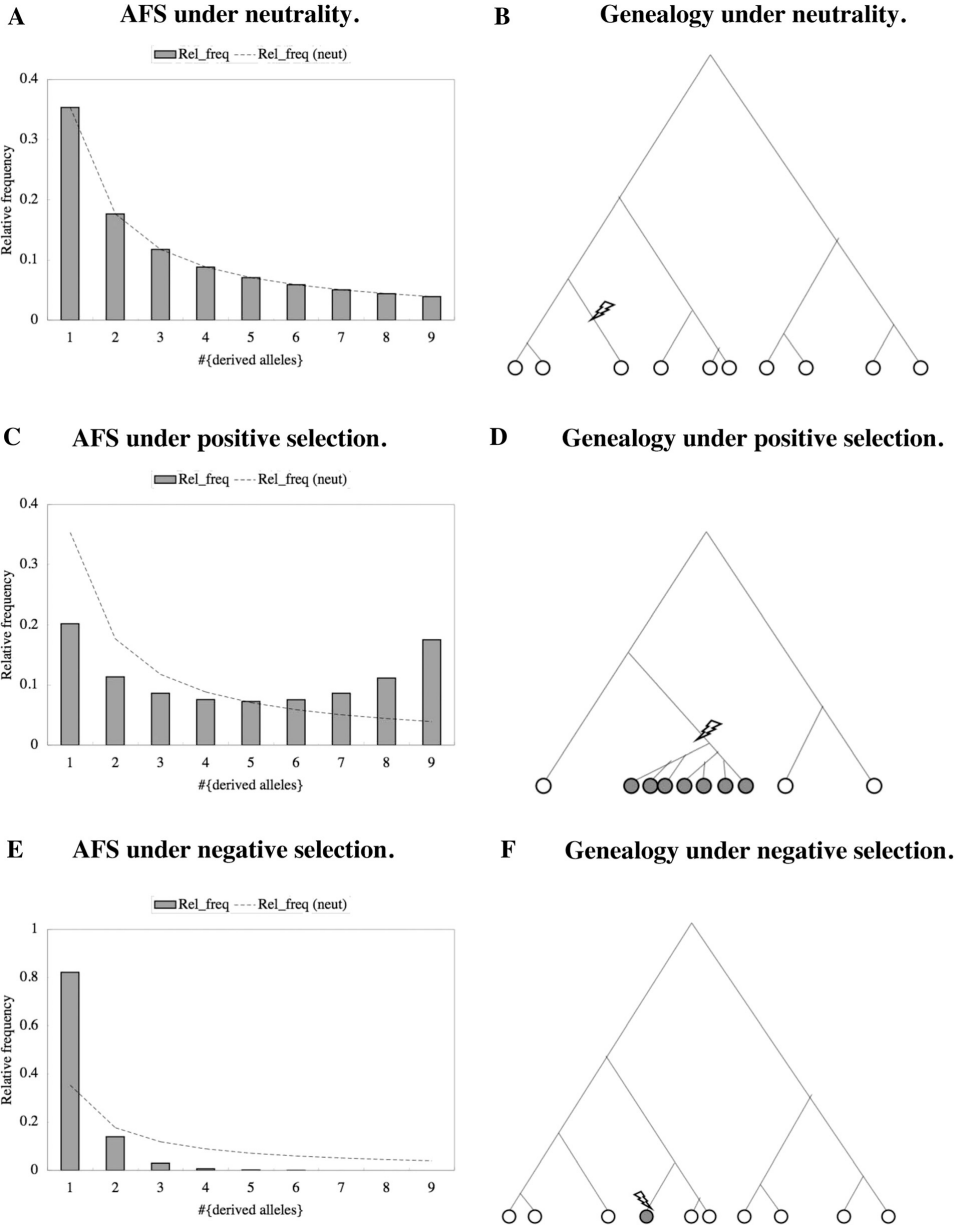
**Impact of selection on allele frequency spectrum and gene genealogy in the infinite-site model.** Panels **A**, **C**, and **E** are schematic allele frequency spectra (AFSs) of the infinite-site model under selective neutrality, positive selection, and negative selection, respectively. Bar graphs are “observed” spectra, and dashed lines are expectations under selective neutrality. Panels **B**, **D**, and **F** are schematic genealogies of n (= 10) sampled sequences that contain a mutation that is selectively neutral, positively selected, and negatively selected, respectively. Open circles are wild type and selectively neutral mutant sequences (or derived alleles). Shaded circles represent mutant sequences that are positively or negatively selected. A lightening bolt represents a mutation event that gave rise to the mutant sequence(s) in each sample. We can see that it is harder to distinguish a single negatively selected mutation event (**E**,**F**) from a neutral one (**A**,**B**) than to distinguish a single positively selected mutation event (**C**,**D**) from a neutral one, because common features of negatively selected mutations are also quite common among selectively neutral mutations. (See Methods for details).

Traditionally, detecting negative selection on a mutant allele has been difficult in a population genetics framework (e.g., [25,51-55]; but see [56]). Let us first explain why this is the case. A common way to detect selection is to compare a test statistic to its distribution under the null hypothesis of selective neutrality (e.g., [5-12]). If the test statistic deviates significantly from the bulk of the null-distribution, the mutant is deemed to be under selection. This strategy has been somewhat successful in detecting positive or balancing selection [18-24], because these selection regimes skew the mutant allele frequency spectrum (AFS) toward high mutant allele frequencies (e.g., Figure 1C), which occur with low probabilities under the selectively neutral infinite-site model (Figure 1A). In terms of a gene genealogy (Figure 1B), we can also say that such selectively positive mutants show strong signals because they often account for a large proportion of sampled sequences (Figure 1D).

Negative selection, on the other hand, skews the mutant AFS toward low frequencies (Figure 1E), which are highly populated even under selective neutrality (Figure 1A). For example, consider the proportion of singleton mutant sites out of all polymorphic sites when *n* sequences are sampled. Under the selectively neutral infinite-site model [26] in a constant-size population, it is approximately given by [7,57]: $\frac{1\;+\;\frac{1}{n-1}}{\sum_{k=1}^{n-1}\frac{1}{k}}$, which is ~19.5% when *n*=100, and ~10.2% even when *n*=10,000. Therefore, even in the extreme case in which a deleterious mutant only leaves a single offspring among as many as 10,000 sampled sequences, the signal of negative selection cannot acquire the statistical significance of less than 5%. (Of course, an individual carrying a negatively selected mutation may not have any offspring at all. We will not discuss such a case because our methods only work with *observed* mutant alleles.) In terms of gene genealogy, we can say that a deleterious mutant modifies the shape of the genealogy only modestly, if any (e.g., [51-55]), because such a mutant tends to occupy only the tip of the genealogy, with fewer offspring lasting for shorter times than neutral ones (Figure 1F). These characteristics have prevented individual events of deleterious mutations from being detected via population genetics methods (e.g., [25] and references therein; but see [56]).

**Figure 2:**
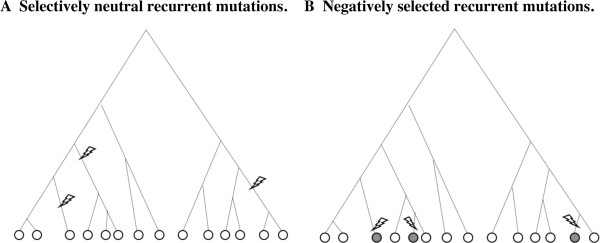
**Selectively neutral and negatively selected recurrent mutations mapped on gene genealogy.** Panels **A** and **B** schematically illustrate recurrent mutations that are selectively neutral and negatively selected, respectively, mapped on genealogies of n (=14) sequences. M=3 mutation events of the same nature are assumed to have occurred along each genealogy. When the mutations are neutral (**A**), at least one of them is likely to result in substantially more than one mutant in the sample. When the mutations are negatively selected (**B**), in contrast, it is likely that every mutation event leaves very few (often one) sampled mutant(s), and the mutant lineages are usually short-lived. NOTATION. In both panels, open and shaded circles represent selectively neutral and negatively selected sampled sequences, respectively. A lightening bolt denotes a mutation event.

However, the situation is totally different if mutations of a particular type occur *recurrently* across the gene genealogy. Let us consider a case where *M*(>1) mutation events of the same type are detected on the genealogy of *n* sampled sequences (Figure 2). If the mutants are selectively neutral, then it is quite likely that at least one of the mutation events resulted in substantially more than one sampled mutant (Figure 2A). In contrast, if the mutants are strongly selected against, it is likely that each of the *M* detected events only left one sampled mutant (Figure 2B) or a few at most. To roughly estimate the probability that each of all the *M* events resulted in only one mutant in the sample, let us assume that the events are mutually independent and that there is no back mutation. Then, for *each* neutral mutation, the probability that it resulted in only one sampled mutant should be approximately given by the relative frequency of true singletons in the infinite-site model, ${\left(\sum_{k=1}^{n-1}\frac{1}{k}\right)}^{-1}$, because the number of resulting mutants should be determined only by the location of the mutation event in the gene genealogy but should not depend on other characteristics of the mutation (under the current assumption). Thus, assuming also that the *M* mutation events do not interfere with one another, the probability that *all* the mutation events resulted in only one sampled mutant each under selective neutrality will be roughly approximated by:

$${\left(\sum_{k=1}^{n-1}\frac{1}{k}\right)}^{-M}.$$

Even with *n*=100, for example, the probability is ~3.7% when *M*=2, and ~0.7% when *M*=3, enabling us to detect negative selection with a sufficient statistical significance. In actual situations, however, the mutation events may interfere with one another, deviating the actual probability from the rough estimation above, and the probability function will depend on the “mutation kinetics,” *i.e.,* possible genetic states and the rates of mutations between the states. Besides, *M* will decrease as the negative selection becomes stronger and as the rate of mutation becomes smaller. Thus, it is not easily predictable how widely applicable our new tests will be. We, therefore, conducted an extensive simulation analysis to examine the actual detection powers of our new tests, as well as their applicable range in the parameter space of mutation rates and selection intensity.

Based on the above rationale, we devised two test statistics. One is the size of the most common class of identical-by-descent mutants in a sample (*Max*^*D*^), which is tested *conditionally on* the number of forward mutations from the ancestral state to the mutant state, *M*, that were mapped on the genealogy. This statistic is denoted by *Max*^*D*^|_*M*_. The other statistic is the total number of mutants in the sample (*Tot*^*D*^), again tested *conditionally on M*. This statistic is denoted by *Tot*^*D*^|_*M*_. The first statistic is somewhat reminiscent of the test statistic in Ewens’ test [5]; their similarities and crucial differences will be explained in *Discussion*. To calculate these test statistics for each subject locus, we need to know the numbers *M* and *Max*^*D*^. These are inferred by using a genealogy of the sampled sequences.

### Detecting negative selection on recurrent mutations using gene genealogy (II): Overall procedure

**Figure 3:**
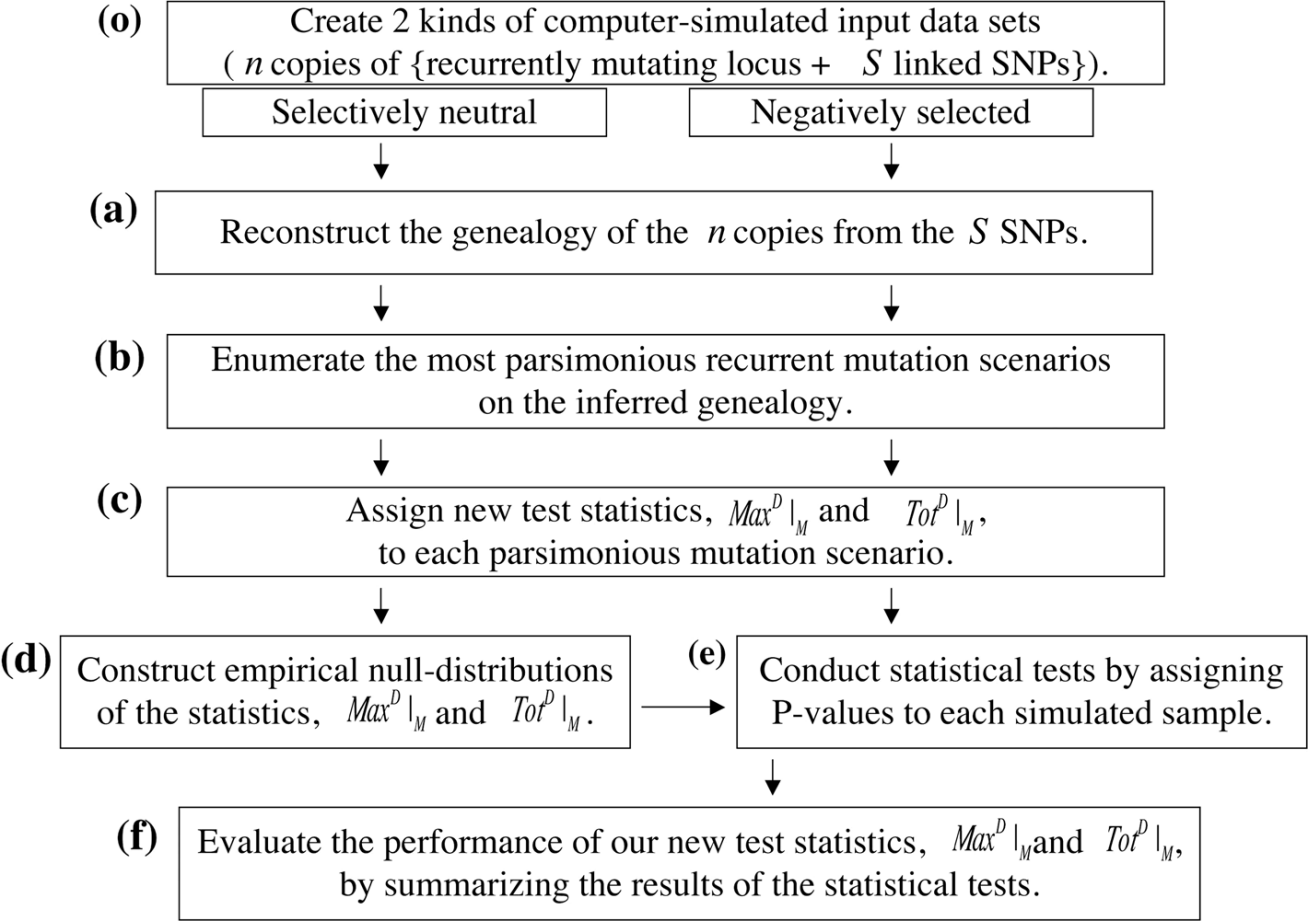
**Overall flowchart of the simulation analysis in this study.** See Methods for details (**o**) and (**a**)-(**f**) designate steps in the overall procedure.

A flowchart for the procedures employed in the new tests is shown in Figure 3. We first need to sample sequences of a locus where recurrent mutations are expected, such as an SV region or a microsatellite, from multiple individuals. Then an allelic state at the locus is assigned to each of the sampled sequences. To infer the genealogy of the sampled sequences, we will use SNPs that either reside in the locus itself or are linked to it. In this study, we create two input data sets by computer simulations, one under selective neutrality and the other under negative selection.

After the input data are obtained, we first infer the genealogy of the sampled sequences using the SNPs [step (a) in Figure 3]. Second, based on the inferred genealogy, we enumerate the most parsimonious mutation scenarios that will realize the allelic states of the sampled sequences with a minimum number of mutations [step (b)]. Third, for each mutation scenario, we will calculate the two test statistics, *Max*^*D*^|_*M*_ and *Tot*^*D*^|_*M*_ [step (c)]. Fourth, the statistics calculated for the mutation scenarios based on selectively neutral loci will be gathered to constitute the “empirical null-distributions” of the statistics [step (d)], which will in turn be used to assign the P-values to each locus that was simulated under negative selection [step (e)]. Finally, the results of such statistical tests will be summarized to evaluate the performance of our new tests [step (f)].

In the following sections, we describe the components of the procedure in more detail.

### Simulations to generate sequence sets under a constant-size population

**Figure 4:**
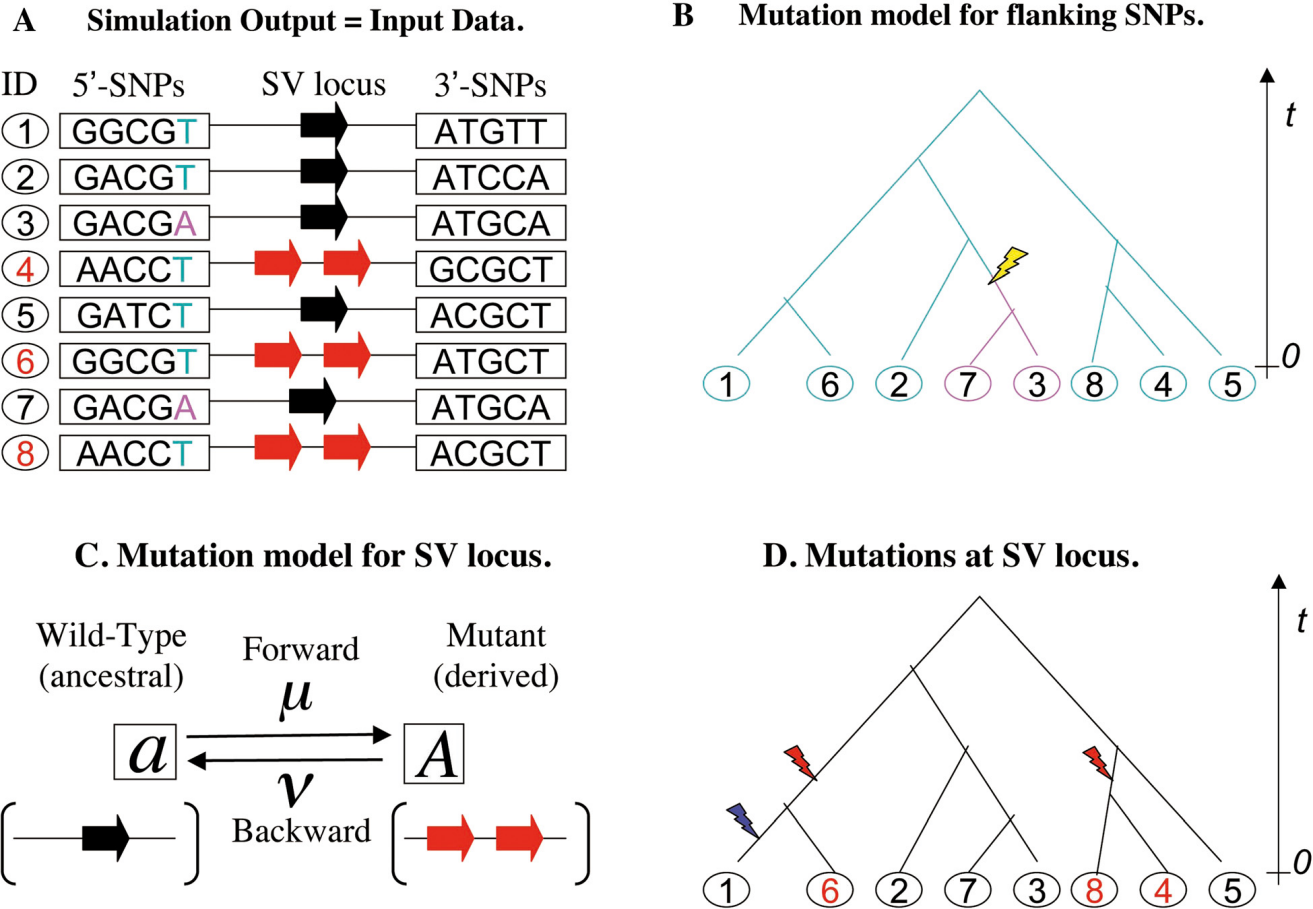
**Schematic illustration of simulation to generate input data.** Panel **A** illustrates a simulation output, which will become an input data to be fed into various neutrality tests. It consists of n sequences sampled from a population, and each sequence is composed of an SV locus flanked by S SNP sites. (In this example, n = 8 and S = 10.) **B.** Flanking SNPs are simulated according to the infinite-site model, which guarantees that each SNP site underwent only one mutation (yellow lightening bolt) along the sequence genealogy. The panel shows the mutation at the SNP site on the immediate left of the SV locus (colored cyan and magenta). **C.** The SV locus is simulated according to the two-state model of recurrent mutations. The character “a“denotes the wild-type (or ancestral) state (here a one-copy state represented by a thick black arrow), and the character “A“denotes the mutant (or derived) state (here a two-copy state represented by two thick red arrows). The forward and backward mutation rates per generation are denoted by μ and v, respectively. **D.** The SV states in this example were generated by two forward mutations (red lightening bolts) and a backward mutation (blue lightening bolt) along the genealogy, resulting in m = 3 sampled mutants (red ID numbers).

Using the *msms* coalescent simulator [49], we created a large input dataset of simulated sequence samples, each consisting of *n* (= 20, 50, 100, or 200) sequences of a recurrently mutating locus accompanied by *S* (= 50) neutral SNPs (Figure 4A), sampled from a constant-size population of *N* (= 10,000) diploid individuals. All simulations were done with no recombination. *msms* simulates SNPs under the infinite-site model [26] (Figure 4B), and the recurrent mutations at the locus under the two-state model [58], with “*a*“and “*A*“denoting the wild-type (ancestral) and mutant (derived) states, respectively (Figure 4C and D). In Figure 4, the wild-type state and the mutant state are single-copied and duplicated, respectively, at the SV locus. Black and red ID numbers in Figure 4 are assigned to sequences with the wild-type state and those with the mutant state, respectively. Let *μ* and *v* denote the forward and backward mutation rates (per locus per haploid genome per generation), respectively (Figure 4C), and *θ*_*μ*_ (≡4*Nμ*) and *θ*_*v*_ (≡4*Nv*) represent the rescaled mutation rates. We used the following mutation rates:

Forward mutation rate: *θ*_*μ*_  =  10^− 1^,  10^− 1/2^,  1 (= 10^0^),  10^+ 1/2^,  10^+ 1^;

Backward/forward ratio: $v/\mu \;=\;0,\;\frac{1}{2},\;1,\;2,\;3$.

Throughout this study, we employed an additive (or genic) selection scheme. The relative fitness values of the ancestral homozygote, heterozygote, and derived homozygote were 1, 1+*s*, and 1+2*s*, respectively. *σ* (≡ 4*Ns*) denotes the rescaled selection coefficient. We used the following selection coefficients::

$$\sigma \;=\;0\;\left(\text{neutral}\right),\;-10^{+1},\;-10^{+3/2},\;-10^{+2},\;-10^{+5/2}.$$

For each of the 4×5×1=20 combinations of *n*,*v*/*μ*, and *σ*=0 for selectively neutral models, we simulated 10,000 samples with *θ*_*μ*_=10^-1^, 5,000 samples with *θ*_*μ*_=10^-1/2^, 3,000 samples with *θ*_*μ*_=1, 3,000 samples with *θ*_*μ*_=10^+1/2^, and 1,000 samples with *θ*_*μ*_=10^+1^. For negatively selected models with *σ*<0, we only used *v*/*μ*=0, 1, 3. For each of the 4×5×3×4=240 combinations of *n*, *θ*_*μ*_, *v*/*μ*, and *σ*<0, we simulated 1,000 samples. It should be noted that the simulations were conducted without regard to the allelic states at the recurrently mutated locus. Thus, the simulated samples include those that could not capture recurrent mutations within the genealogy, in addition to those that could.

### Inferring gene genealogies and mutation scenarios (brief description)

The genealogy among the sequences in each simulated sample was first inferred via the Neighbor-Joining (NJ) method [59] using the number of SNP sites with different states as a pairwise distance between two sequences. Second, we removed interior branches not supported by any SNP site (Additional file 1: Figure S1F). Third, we placed a root at the mid-point between the most distant pair of sequences. Fourth, because all existing parsimony algorithms (e.g., [60,61]) may overestimate the number of mutations under some circumstances (Additional file 1: Figure S1G), we mapped mutation events at the recurrently mutating locus onto the resulting “SNP-supported tree” by using a new maximum parsimony algorithm that we have especially designed for this purpose. The new algorithm enumerates all possible mutation scenarios that could result in the minimum number of mutations, each accompanied by additional interior branches necessary to realize the scenario (Additional file 1: Figure S1H). The section “*Inferring Gene Genealogies and Mutation Scenarios (Rationale)*” of *Supplementary methods* in *Supplementary Notes* (Additional file 1) describes the rationale behind this new parsimony algorithm and our genealogy reconstruction method. Additional file 2 is dedicated entirely to a detailed description of this new parsimony algorithm.

### New statistical tests to detect negative selection

Given an empirical cumulative null-distribution for *Max*^*D*^|_*M*_ or *Tot*^*D*^|_*M*_ as defined by Equations (S5a,b) in *Supplementary methods* in Additional file 1, we can define the empirical P-value. When a parsimonious scenario for a sequence set, which is in general under selection, has *Max*^*D*^|_*M*_  =  *x*^*Obs*^, the empirical P-value of the scenario under the “null-hypothesis,” *Y*, is::

(1a) $$P{\;}^{E}\left(\text{scenario}\;\text{with}\;\text{Ma}x^{D}{|}_{M}=x^{\text{Obs}}\right)\;\equiv \;P_{0}^{E}\left[\text{Ma}x^{D}{|}_{M}\leq {\bar{x}}^{\text{Obs}}\;\left|\right)\;Y\right].$$

To be conservative, we defined ${\bar{x}}^{\text{Obs}}$ as *x*^*Obs*^ if it is in the domain of the null distribution, or otherwise the smallest value of *Max*^*D*^|_*M*_ among those greater than *x*^*Obs*^ in the domain of the null distribution. Similarly, the empirical P-value of a scenario with *Tot*^*D*^|_*M*_  =  *x*^*Obs*^ under the “null-hypothesis,” Y, is defined as::

(1b) $$P^{E}\;\left(\text{scenario}\;\text{with}\;\text{To}t^{D}{|}_{M}=x^{\text{Obs}}\right)\;\equiv \;P_{0}^{E}\left[\text{To}t^{D}{|}_{M}\leq {\bar{x}}^{\text{Obs}}\;\left|\right)\;Y\right].$$

Then we estimated the empirical P-value *of a sequence set*, which the new neutrality test actually uses, with the average of the empirical P-values over parsimonious scenarios:

(2) $$P{\;}^{E}\left(\text{sequence}\;\text{set}\right)\;=\;\frac{\sum_{\text{parsimonious}\;\text{scenarios}\;\text{for}\;\mathrm{the}\;\text{sequence}\;\text{set}}P^{E}\;\left(\text{scenario}\right)}{\#\left\{\text{parsimonious}\;\text{scenarios}\;\text{for}\;\mathrm{the}\;\text{sequence}\;\text{set}\right\}}$$

where *P*^*E*^(*scenario*) is (1a) and (1b), when the test statistic is *Max*^*D*^|_*M*_ and *Tot*^*D*^|_*M*_, respectively.

### Performance tests under expanding population

We also examined the performance of our new statistical tests on sequence data sets simulated under a population that expanded recently. As an expanding population, we used a simple model that broadly reproduces the European demography inferred by [62]. In terms of forward time evolution, the model population begins with an ancestral (bottleneck) population at equilibrium with the constant size *N*_*B*_=2100. Then the population is shrunk to *N*_*EU*0_=1000 at *T*_*EU-AS*_=21200 years ago (when it separated from the Asian population), and then it expands exponentially. For the expansion rate, *r*, we used the maximum-likelihood estimate for the European population, *r*_*EU*_=4.0×10^-3^ per generation and a generation time of 25 years. We also used the lower and upper bounds of the parametric bootstrap bias-corrected 95 % confidence interval, *r*_*EU*_=2.6×10^-3^ and 5.7×10^-3^ per generation [62].

Other parameters were basically the subsets of those used for the performance tests under the constant-size population. A caveat is that population genetic parameters are rescaled so that their *raw* values (but *not* their population-scaled values) match the values for the constant population of size *N*=10000. More specifically, we used sample sizes of *n* = 100 and 200, backward/forward ratios of *v*/*μ* = 0, 1, and 3, and selection coefficients equivalent to *σ*  =  0 (*neutral*),   − 10^+ 3/2^,   − 10^+ 2^,   − 10^+ 5/2^. As for the forward mutation rate, *θ*_*μ*_, we used the same exact setting as for the constant-size population.

We conducted two performance tests. First, we examined the performance of our new tests just as we did under the constant-size population, assuming that the expansion rate *r*=*r*_*EU*_ was inferred exactly. Second, to examine the effect of erroneous inference of *r*=*r*_*EU*_, our new tests with the empirical null-distributions computed with *r*_*EU*_=4.0×10^-3^ were applied to the sequence sets simulated under *r*_*EU*_=2.6×10^-3^ and *r*_*EU*_=5.7×10^-3^.

## Results

### Performance of our new parsimony algorithm

The new neutrality tests as described in this paper depend on a new parsimony algorithm that we developed to map mutation events on the sequence genealogy. Therefore, we first compared the new parsimony algorithm with traditional tree reconstruction algorithms, in terms of the accuracy of tree reconstruction. As a representative of the traditional tree reconstruction algorithm, we used the neighbor-joining (NJ) method [59]. We first note that, under the current situation where a tree is reconstructed only from sites following the infinite-site model, the NJ method should infer trees as accurately as the maximum-likelihood (ML) method, which is known to be the most accurate under most situations. A problem is that most traditional tree reconstruction algorithms forcefully infer a fully resolved tree by randomly inserting (zero-length) branches to “resolve” practically multifurcated nodes. Our new parsimony algorithm solves this problem by starting with a multifurcated tree whose branches are all supported by SNP sites, and further resolving phylogenetic relationships by taking advantage of the recurrent mutations (see Additional files 1 and 2 for details). To make sure that this strategy actually works, we applied both the NJ method and our new parsimony algorithm to each sequence set simulated as detailed in the next subsection, and compared the reconstructed trees with the true genealogy among simulated sequences. When the sample size *n*=100 and *v*/*μ*=1, for example, each NJ tree has 73±5 false-positive branches (the numbers represent mean±standard deviation), while each tree via our new parsimony has on average 1±1 false-positive branches. Next we defined the “additional true-branch rate” as $\frac{\mathrm{ATP}}{\mathrm{ATP}+\mathrm{FP}}$, where *ATP* is the number of true-positive branches not supported by SNPs, and *FP* is the number of false-positive branches. Under these conditions, the additional true-branch rate of our new parsimony algorithm (0.378±0.298) was more than five times higher than that obtained by the NJ method (0.071±0.035). Results were similar under other conditions (as long as the sample size was quite large). Additional file 1: Tables S2 and S3 show the results in more details.

### Frequency of recurrent mutations captured by gene genealogy

Because our new tests are only useful when recurrent mutations are detected on a genealogy of sampled sequences, we first examined the relative frequencies of recurrent mutations that can be captured by gene genealogies out of the cases where the recurrently mutating locus is polymorphic. Table 1 and Additional file 1: Tables S4 and S5 summarize the relative frequencies for the backward/forward ratios, *v*/*μ* = 0, 1 and 3, and the numbers of sampled sequences, *n* = 100, 50 and 200. Roughly speaking, deletions should typically have *v*/*μ* = 0, because undoing a deletion is usually impossible. Inversions should have *v*/*μ* around 1 because of the symmetry between forward and backward mutations. Duplications are known to have *v*/*μ*≥2 (e.g., [63]), so we chose *v*/*μ* = 3 as a representative value. As expected, the frequency of detected recurrent mutations increases as the mutation rate increases, as the negative selection becomes weaker, and as the sample size increases. For *v*/*μ* = 0, the “NA” marks are seen at high forward mutation rates (*θ*_*μ*_ ≥ 10^+ 1/2^) and at weak negative selection (*σ* ≥ − 10) (section A of the tables). This is because these cases have no back mutations to prevent the frequent forward mutations from fixing the mutant state in the population.

**Table 1:** Relative frequencies of recurrent mutations captured by gene genealogy, out of polymorphic loci

**A.** v/*μ*=0.					
*θ*_*μ*_	*σ*=0 (neutral)	*σ* = − 10	*σ* = − 10^3/2^	*σ* = − 10^2^	*σ* = − 10^5/2^
10^-1^	0.091	0.141	0.071	0.014	0.000
10^-1/2^	0.231	0.216	0.190	0.057	0.026
1(=10^0^)	0.470	0.625	0.467	0.238	0.102
10^+1/2^	NA^a^	0.856	0.905	0.648	0.300
10^+1^	NA	NA	0.995	0.981	0.759
**B.***v*/*μ*=1.					
*θ*_*μ*_	*σ*=0(neutral)	*σ* = − 10	*σ* = − 10^3/2^	*σ* = − 10^2^	*σ* = − 10^5/2^
10^-1^	0.097	0.080	0.029	0.041	NA
10^-1/2^	0.306	0.212	0.165	0.082	0.026
1(=10^0^)	0.744	0.668	0.463	0.263	0.104
10^+1/2^	0.986	0.966	0.898	0.628	0.313
10^+1^	1.000	1.000	1.000	0.980	0.729
**C.***v***/***μ*=3.					
*θ*_*μ*_	*σ*=0(neutral)	*σ* = − 10	*σ* = − 10^3/2^	*σ* = − 10^2^	*σ* = − 10^5/2^
10^-1^	0.170	0.098	0.039	0.027	NA
10^-1/2^	0.434	0.226	0.159	0.073	0.042
1(=10^0^)	0.797	0.631	0.412	0.215	0.104
10^+1/2^	0.987	0.963	0.864	0.622	0.338
10^+1^	0.999	0.999	0.997	0.961	0.713

NOTE. The results are shown for sets with *n*=100 sampled sequences each. *v*/*μ* is the backward/forward ratio of mutation rates. *θ*_*μ*_(≡ 4*Nμ*) is the rescaled forward mutation rate. *σ*(≡ 4*Ns*) denotes the rescaled selection coefficient.

^a^ “NA” is assigned to a category with less than 30 polymorphic loci.

Although we also examined the simulations with *n* = 20, their gene genealogies rarely captured the recurrent mutations unless the forward mutation rate is extremely high (*θ*_*μ*_ ≥ 10). Thus, we judged that our new test is useful only when the sample size is fairly large, and focused on the case of *n* = 100, unless otherwise stated.

### Number of mutations mapped on the gene genealogy

**Figure 5:**
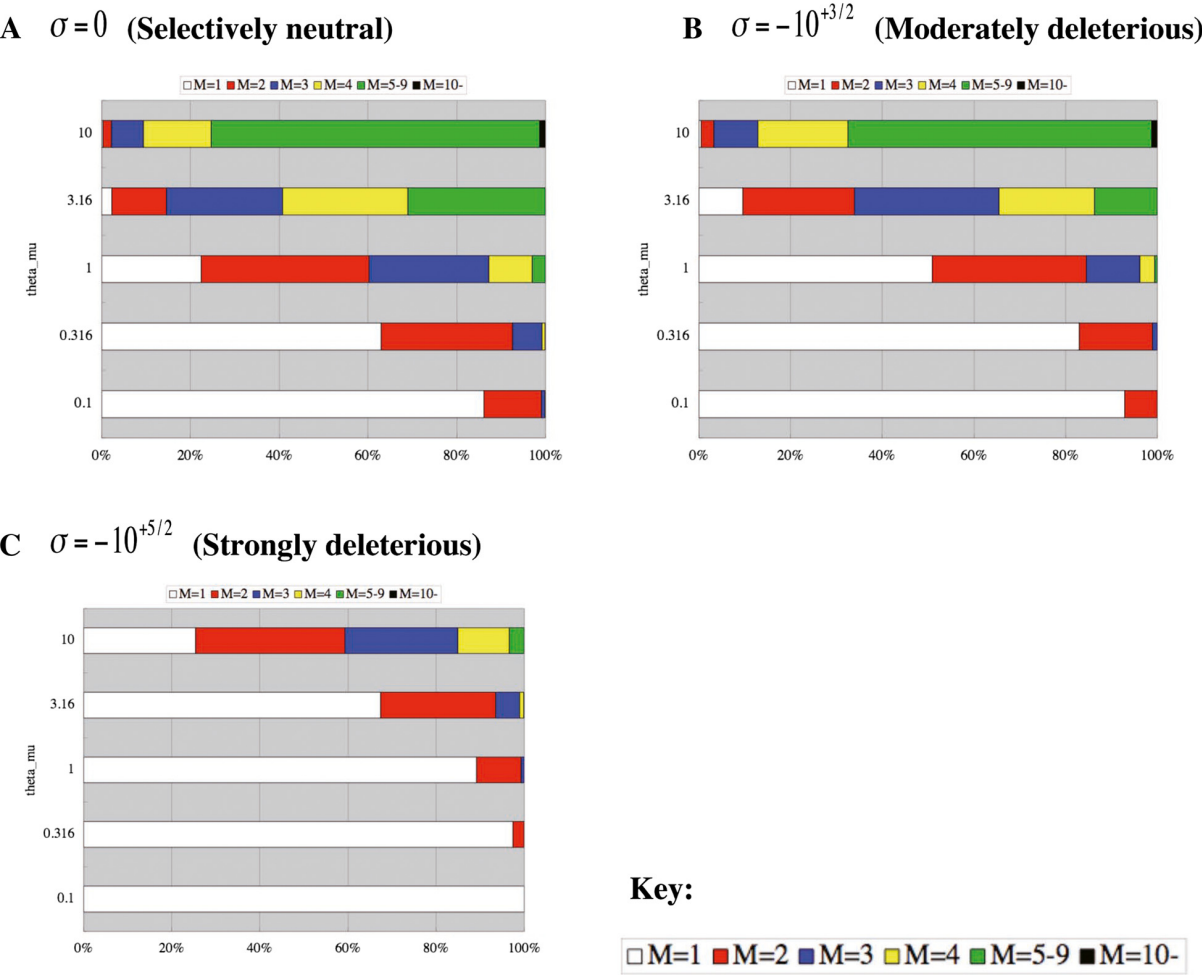
**Composition of the number of mutations at each recurrently mutating locus.** This figure is for a fixed backward/forward ratio (v/μ = 1) and a fixed sample size (n = 100). The composition of the number M of forward mutations mapped on a genealogy, among recurrently mutating loci showing polymorphism, is shown under various forward mutation rate (θμ) and the selection intensity (σ). Panels **A**, **B**, and **C** give results with σ=0**,***σ*  =   − 10^+ 3/2^, and *σ*  =   − 10^+ 5/2^, respectively. In each panel, a horizontal bar shows the composition for a value of θμ specified on the left. In each horizontal bar, white, red, blue, yellow, green and black rectangles represent the proportions of mutation loci with M = 1, 2, 3, 4, 5–9, and 10-, respectively.

The horizontal bar graphs (spectra) in Figure 5 show the proportions of the parsimonious scenarios classified with the number of forward mutations mapped on each gene genealogy (*M*), with various combinations of the forward mutation rate (*θ*_*μ*_) and the selection intensity (*σ*), under fixed values of *v*/*μ* (= 1) and *n* (= 100). We can see that the classes with many mutations increase in proportion as the mutation rate becomes higher and the negative selection becomes weaker. Another noticeable point is that highly deleterious mutations (e.g., with *σ* =   − 10^+ 5/2^) that are quite frequent (e.g., with *θ*_*μ*_  =  10^+ 1/2^,  10^+ 1^) have spectra of mutation numbers very similar to those of selectively neutral mutations with modest mutation rates (e.g., *θ*_*μ*_  =  10^− 1/2^,  10^0^). This phenomenon is understandable because the average number of mutations should correlate positively with the mutation rate and negatively with the selection coefficient. The mutation-number composition depends quite slightly on *v*/*μ* (compare Figure 5 with Additional file 1: Figures S2 and S3). These results suggest that, unless we know the mutation rates (i.e., *θ*_*μ*_ and *v*/*μ*) in advance, it is dangerous to use a statistic for detecting negative selections that strongly correlates with *M*. Such a statistic would confuse the effects of mutation rates with those of selection. This led us to the new test statistics, *Max*^*D*^|_*M*_ and *Tot*^*D*^|_*M*_, which are conditional on *M*.

### Distributions of new test statistics under selective neutrality and negative selection

To detect negative selection on recurrent mutations, we devised two test statistics, *Max*^*D*^|_*M*_ and *Tot*^*D*^|_*M*_. The statistic *Max*^*D*^|_*M*_ is the size of the most common class of identical-by-descent mutants in the sample (at the recurrently mutating locus) inferred with a genealogy (*Max*^*D*^), tested *conditionally on* the number of forward mutation events (*M*). The statistic *Tot*^*D*^|_*M*_ is the total number of mutants in the sample (*Tot*^*D*^(≡*m*)), again tested *conditionally on M*. Briefly, these test statistics are expected to be smaller under negative selection than under neutrality, because the descendants of deleterious mutants are unlikely to proliferate. And, because *M* is fixed, the statistics are expected to be mostly immune to the problem discussed in the last section.

**Figure 6:**
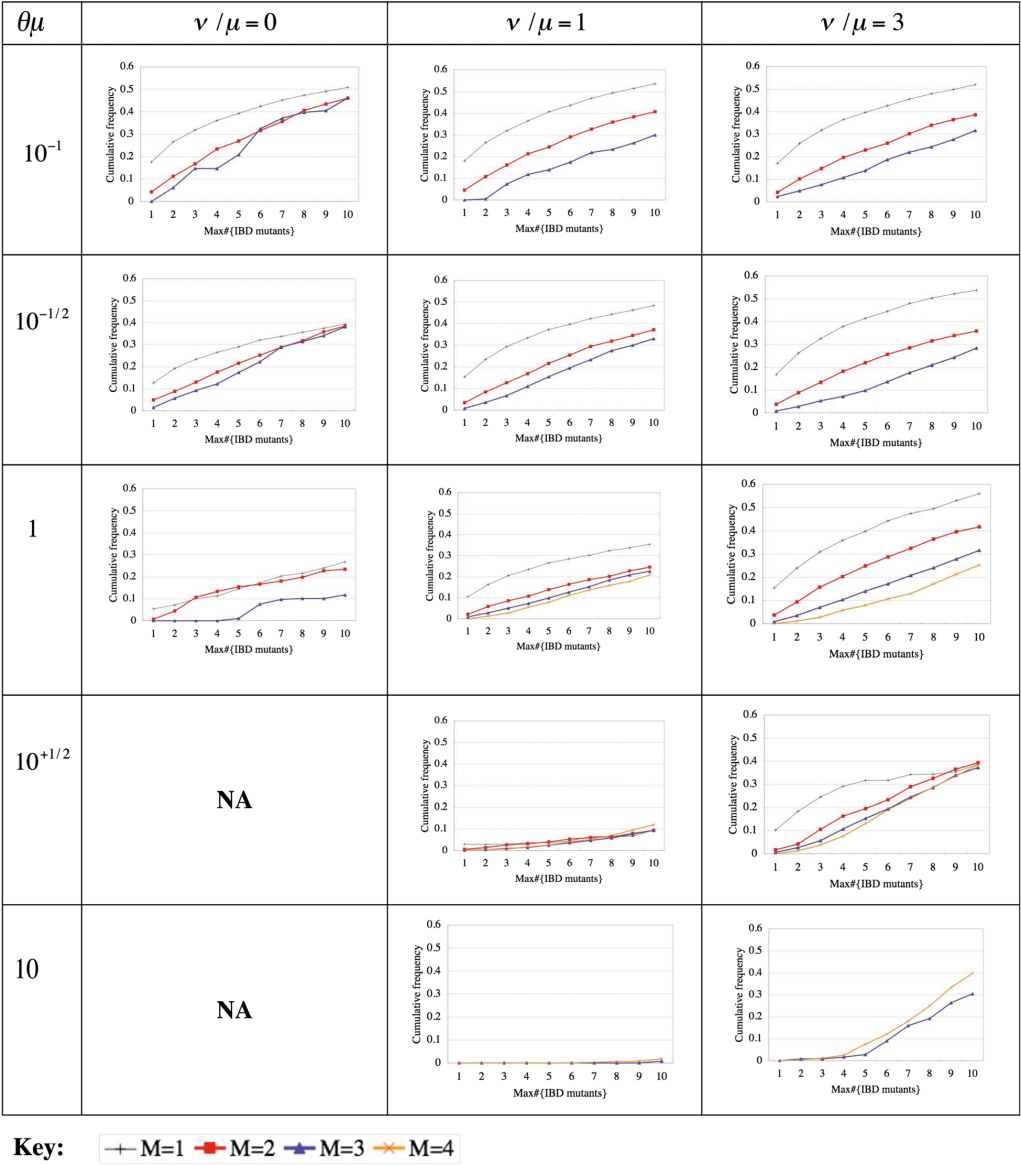
**Cumulative distributions of our new test statistic, *Max***^***D***^|_***M, ***_**under selective neutrality.** Each panel shows the cumulative distributions of our new test statistic, *Max*^*D*^|_*M,*_ for v/μ specified by the column and θ_μ_ specified by the row. The selection coefficient is fixed at σ=0 (selectively neutral), and the sample size is fixed to be n = 100. In each panel, a thin black line shows the cumulative distribution for M =1 as a control, and bold lines colored red, blue, and orange represent the distributions for M = 2, 3, and 4, respectively. “NA” and missing lines indicate that the categories in question did not gather enough numbers of simulated loci.

Figure 6 and Additional file 1: Figure S4 show the distributions of the new test statistics, *Max*^*D*^|_*M*_ and *Tot*^*D*^|_*M*_, respectively, under selective neutrality (*σ*=0) and different combinations of mutation rate parameters. When the mutation rate is low (*θ*_*μ*_  ≤  10^− 1/2^), the distributions depend little on *θ*_*μ*_ or *v*/*μ*. This is understandable given that mutation events are likely to be sparse on the genealogy and that backward mutations should impact the distributions only slightly, if at all, under low mutation rates. As the mutation rate becomes larger (*θ*_*μ*_ ≥  10^0^ (=1)), small values of the test statistics get less and less common, and this tendency is conspicuous for smaller *ν*/*μ* values. Probably, this is partly because parsimony methods tend to underestimate the number of mutation events as the mutation rate increases. Nevertheless, such dependence on the mutation rate will only make our new test statistics more conservative (in terms of false positive rate). We also examined the distributions of *Max*^*D*^|_*M*_ at strongly deleterious loci with *σ*=-100 (Additional file 1: Figure S5). We can observe that the cumulative distributions rapidly converge to 1, and the comparison with the null-distribution (Figure 6) implies a high yield. Taken together, these properties of the distributions of *Max*^*D*^|_*M*_ and *Tot*^*D*^|_*M*_ indicate that they are not only fairly powerful “neutrality tests,” but also robust against variation in the mutation rate.

### Performance of our new neutrality tests to detect negative selection on recurrent mutations

In the above section, the distributions were obtained under fixed values of *θ*_*μ*_ and *v*/*μ*. We have to remember, however, *θ*_*μ*_ is usually unknown. Although *v*/*μ* may be figured out to some extent if the type of the recurrent mutation is known, this may not always be the case. Thus, we defined the null-distributions of the test statistics, *Max*^*D*^|_*M*_ and *Tot*^*D*^|_*M*_, by assuming that the forward mutation rate is power-law distributed, i.e., *P* [ *θ*_*μ*_ > *X* ]  = *A* · *X*^− *α*^, where *α* is the exponent that specifies the power-law. Recent genome-scale data analyses on CNVs indicated that a majority of CNV loci show low rates, satisfying *θ*_*μ*_<0.1 (e.g., [42]), and that quite a large number of CNV loci have high rates, satisfying *θ*_*μ*_>1 or even *θ*_*μ*_>10 (e.g., [45]). Power-law distributions could interpolate such observations well. We used the values *α* = 0.5, 1, and 2, which seem to span a reasonable range. Relative weights of *θ*_*μ*_ are shown in Additional file 1: Table S1. The exponent *α* = 0.5 seems to give proportions somewhat similar to those obtained by [45] for CNV loci with high mutation rates, and *α* = 2 consists almost exclusively of the lowest mutation rate (*θ*_*μ*_ = 0.1). The performance of our new tests remained almost unchanged across *α* = 0.5, 1, and 2 (compare e.g., Table 2 with Additional file 1: Tables S6 and S7). So, we will only show the results for *α* = 1. Regarding *v*/*μ*, we prepared two different null-distributions: one with a fixed value of *v*/*μ* that is assumed as known in advance, and the other with the null-distribution averaged over unknown values of *v*/*μ*. The specific definitions of the null-distributions are described in *Methods* and in Additional file 1. Surprisingly, our new tests with unknown *v*/*μ* performed almost as well as those with known *v*/*μ* (compare e.g., Table 2 with Additional file 1: Table S8). Thus, in the following, we will only show the results when *v*/*μ* is unknown.

**Table 2:** **False positive and true positive rates via***Tot*^*D*^|_*M*_**, when *v */ *μ *is not known in advance**

**A.** v/*μ*=0.					
*θ*_*μ*_	*σ*=0(neutral)	*σ* = − 10	*σ* = − 10^3/2^	*σ* = − 10^2^	*σ* = − 10^5/2^
10^-1^	0.055	0.063	0.182	NA	NA
10^-1/2^	0.059	0.052	0.162	0.333	NA
1(=10^0^)	0.005	0.097	0.294	0.521	0.615
10^+1/2^	NA^a^	0.021	0.208	0.543	0.774
10^+1^	NA	NA	0.008	0.488	0.699
**B.***v*/*μ*=1.					
*θ*_*μ*_	*σ*=0 (neutral)	*σ* = − 10	*σ* = − 10^3/2^	*σ* = − 10^2^	*σ* = − 10^5/2^
10^-1^	0.057	0.063	NA	NA	NA
10^-1/2^	0.038	0.175	0.203	0.625	NA
1(=10^0^)	0.028	0.110	0.339	0.519	0.739
10^+1/2^	0.009	0.058	0.266	0.497	0.706
10^+1^	0.000	0.002	0.081	0.527	0.728
**C.***v*/*μ*=3.					
*θ*_*μ*_	*σ*=0 (neutral)	*σ* = − 10	*σ* = − 10^3/2^	*σ* = − 10^2^	*σ* = − 10^5/2^
10^-1^	0.051	0.000	NA	NA	NA
10^-1/2^	0.037	0.118	0.351	0.500	NA
1(=10^0^)	0.037	0.140	0.240	0.528	0.815
10^+1/2^	0.050	0.131	0.293	0.547	0.746
10^+1^	0.078	0.142	0.293	0.585	0.734

NOTE. Here, *n*=100 and *α* = 1, as well as 5% nominal significance level, are fixed. The tables show proportions of loci that tested positive via *Tot*^*D*^|_*M*_ out of those whose gene genealogies revealed recurrent mutations. *ν*/*μ* is the backward/forward ratio of mutation rates. *θ*_*μ*_(≡ 4*Nμ*) is the rescaled forward mutation rate. *σ*(≡ 4*Ns*) denotes the rescaled selection coefficient.

^a^ “NA” is assigned to a category with less than 10 loci with revealed recurrent mutations.

With the null-distributions at hand, we examined the performance of our new tests by applying them to the samples of sequences simulated under negative selection. We chose the nominal significance level of 5%. To figure out the actual rate of false-positives (i.e., type I errors), we also applied the tests to sequence samples simulated under selective neutrality. Overall, the two test statistics performed similarly well, with *Tot*^*D*^|_*M*_ performing slightly better than *Max*^*D*^|_*M*_ (compare e.g., Table 2 with Additional file 1: Table S9). Thus, henceforth, we will only show the results for *Tot*^*D*^|_*M*_. Table 2, Additional file 1: Tables S10 and S11 show the proportions of simulated samples with size *n* = 100, 50, and 200, respectively, that tested positive via *Tot*^*D*^|_*M*_ (under *α* = 1 and using null-distributions for unknown *v*/*μ*), out of the samples whose gene genealogies identified recurrent mutations. The proportions could be regarded as true positive rates if the simulations are under negative selection, and as false-positive rates if the simulations are under selective neutrality. Both tests demonstrate high true-positive rates of ~50-80%, while keeping the false-positive rates down to around 5% or less, for strongly negative selection (with *σ* = − 10^+ 2^,   − 10^+ 5/2^) and with large sample sizes (*n* = 100 and 200) (Table 2 and Additional file 1: Table S11). Although the true positive rates somewhat dropped for moderately negative selection (with *σ* = − 10^+ 3/2^), still 10-30% of the cases were detected. On the other hand, the true positive rates for weakly deleterious mutations (with *σ*=-10) were marginal, hovering around 10% or less. Thus our new tests will have little power when detecting weak negative selection on recurrent mutations, no matter how frequently the mutations occur. The tests suffered low positive rates also under weak to moderate selection (with *σ* ≥ − 10^+ 3/2^) with a very high mutation rate (with *θ*_*μ*_ = 10), probably because independent forward mutations were erroneously merged on incompletely resolved gene genealogies, which is inevitable. Or it may also be because an excessively high number of forward mutations could in principle prevent *Tot*^*D*^|_*M*_ and *Max*^*D*^|_*M*_ from clearly distinguishing between deleterious mutations and selectively neutral ones.

For a medium sample size (*n* = 50), the true-positive rate is reduced to less than 30% (Additional file 1: Table S10). This is because the null-distributions of *Max*^*D*^|_*M*_ and *Tot*^*D*^|_*M*_ are “inherently discrete,” namely, their smallest non-zero probabilities are slightly greater than 5% for *M* = 2 when *n* = 50.

### Performance of new neutrality tests under expanding populations

Populations of many species including humans are thought to have expanded recently (*e.g*., [64-67]). The population growth is known to increase the number of low-frequency polymorphisms, displaying signals similar to those of negative selection (*e.g*., [68-70]). A recent trend in population genetic analyses is to incorporate such demographic effects into the null-distributions, by inferring the demographic effects independently from a genome-wide collection of selectively neutral polymorphic sites, such as synonymous SNPs [71,72]. Thus, we also examined the performance of our new neutrality tests under such settings. We simulated sequence samples under an expanding population with growth rates of *r*=4.0×10^-3^, 2.6×10^-3^, and 5.7×10^-3^ per generation, which respectively correspond to the maximum likelihood estimate, the lower- and the upper-bounds of 95% confidence interval inferred for the European population [62]. Using the samples simulated under selective neutrality, we constructed empirical null distributions under each growth rate. We first applied our new statistical tests to the samples simulated under negative selection and under the same growth rate that generated the null distribution. Because the null-distributions of *Max*^*D*^|_*M*_ and *Tot*^*D*^|_*M*_ are discrete, and because the allele frequency spectrum under an expanding population skews toward rare alleles, we expected (and confirmed) that a fixed nominal significance level of 5% will result in a low detection rate (data not shown). Thus, we set the nominal significance level at infinitesimally above the probability of *Tot*^*D*^=2 (or equivalently *Max*^*D*^=1), conditional on *M*=2. The new tests exhibited reasonably high detection rates (Table 3). The false positive rates were reasonably low for high mutation rates. Although false-positive rates were quite high for low mutation rates, this may not cause a serious problem, because the detection rates were 2 to 3 fold higher than the false positive rate, and because it is only very rarely that polymorphic loci with low mutation rates show recurrent mutations among the samples (Additional file 1: Table S12). For example, only 7.0-13.3% of neutral polymorphic loci with *θ*_*μ*_=0.1 had *M*≥2. Still, some other statistics that help roughly infer *θ*_*μ*_ or some prior knowledge on *θ*_*μ*_ could be exploited to validate the results of the new tests.

**Table 3:** **False positive and true positive rates via***Tot*^*D*^|_*M*_**, when *v */ *μ *is not known in advance, under expanding population (with correct *r *)**

**A.** r=2.6 × 10^-3^				
*θ*_*μ*_	*σ*=0 (neutral)	*σ* = − 10^3/2^	*σ* = − 10^2^	*σ* = − 10^5/2^
10^-1^	0.185	0.125	NA ^a^	NA
10^-1/2^	0.179	0.315	0.500	NA
1(=10^0^)	0.095	0.184	0.352	0.600
10^+1/2^	0.056	0.141	0.340	0.580
10^+1^	0.018	0.058	0.283	0.551
**B.***r*=4.0 × 10^-3^				
*θ*_*μ*_	*σ* = 0 (neutral)	*σ* = − 10^3/2^	*σ* = − 10^2^	*σ* = − 10^5/2^
10^-1^	0.341	0.333	NA ^a^	NA
10^-1/2^	0.265	0.259	0.516	NA
1(=10^0^)	0.187	0.258	0.509	0.886
10^+1/2^	0.081	0.210	0.491	0.818
10^+1^	0.026	0.115	0.408	0.668
**C.***r*=5.7 × 10^-3^				
*θ*_*μ*_	*σ*=0 (neutral)	*σ* = − 10^3/2^	*σ* = − 10^2^	*σ* = − 10^5/2^
10^-1^	0.372	0.583	0.833	NA ^a^
10^-1/2^	0.288	0.376	0.680	NA
1(=10^0^)	0.236	0.423	0.655	0.957
10^+1/2^	0.080	0.252	0.524	0.780
10^+1^	0.022	0.119	0.401	0.607

NOTE. Here, *n*=100, *α* = 1, and the backward/forward ratio *v*/*μ*=1 are fixed. The nominal significance level is set slightly above the relative frequency of *Tot*^*D*^ = 2 conditional on *M*=2. The tables show proportions of loci that tested positive via *Tot*^*D*^|_*M*_ out of those whose gene genealogies revealed recurrent mutations. *θ*_*μ*_(≡ 4*Nμ*) is the rescaled forward mutation rate. *σ*(≡ 4*Ns*) denotes the rescaled selection coefficient. Here the null distributions are based on the correct recombination rate *r* (per generation).

^a^ “NA” is assigned to a category with less than 10 loci with revealed recurrent mutations.

In actual data analyses, the estimated population growth parameter should suffer some uncertainties (see e.g., [62]). To examine the impacts of such uncertainties, we applied our new tests on the data sets simulated under the both ends of the 95% confidence interval, *r*=2.6×10^-3^ and 5.7×10^-3^, using the null distributions estimated from simulations of neutral mutations with the above MLE, *r*=4.0×10^-3^. Our new tests retained almost the same performance as those using the correct growth parameters (Additional file 1: Table S13), demonstrating that the tests are robust under these uncertainties.

## Discussion

In this study, we introduced two new population genetics tests to detect negative selection on recurrent mutations. Our computer simulation analyses demonstrated high powers of these tests to detect recurrent deleterious mutations in constant-size populations, and moderate detection powers in expanding populations. To the best of our knowledge, this is the first ever attempt to detect negative selection by using recurrent mutations, and our tests turned out to be superior to traditional neutrality tests that do not fare well in this respect. To illustrate this point, we also applied some widely used traditional neutrality tests, Ewens’ test [5], the Ewens-Watterson test [6], and Tajima’s D test [7], to our constant-population dataset (Additional file 1). We found that these tests detected selection only slightly better than expected by chance (Additional file 1: Tables S14, S15 and S16). This is understandable because applying a traditional neutrality test to SNPs in the flanking regions of a locus undergoing recurrent deleterious mutations is tantamount to attempting to detect “background selection” on a linked genomic region *using only information from a single locus*, which was shown to be very difficult (e.g., [73]). Of course, out tests will not undermine the value of these traditional neutrality tests, because they are known to detect other types of deviations from the standard neutral population genetic model (see e.g., [12,25,74]).

### Outstanding issues

We should keep in mind that this study is merely a first step, because the tests have so far been applied to only the simplest cases (a selectively neutral background without recombination in a constant-size population or a regularly expanding population). For future tests to be really useful, we will have to examine how robust the tests are against various confounding factors, such as population substructure and migration (e.g., [62,75,76]), background selection, recombination, and mutation kinetics. Although such analyses were not conducted in this study, we may be able to roughly predict the effects of some of such factors and potential countermeasures.

Recombination will confound the inference of gene genealogy, possibly causing false-positives e.g., by splitting the descendant cluster of a forward mutation event, and false-negatives e.g., by merging the descendant clusters of two independent mutation events. Such factors may only have modest effects on our new tests, because our choice of the number of flanking SNPs (*S*=50) is similar to the typical number of SNPs within a haplotype block in the human genome (e.g., [27,28]), and because mutant clusters under detectable negative selection are usually too small for recombination to either split or merge. Nevertheless, recombination may impact our tests at least occasionally, especially when the subject locus spans and/or is flanked by more than one haplotype block. To be robust under such effects, we will have to grade up our tests so that they can handle multiple genealogies arranged along a tested region.

Another issue that should be explored in the future is the modeling of mutation kinetics. Although we found that the test results do not substantially differ across a wide range of backward/forward ratios, from *v*/*μ* = 0 to *v*/*μ* = 3, they are just within the two-state model [58]. Recurrent mutations could occur more frequently at multistate loci, which might be describable only by their own particular models, such as multisite models (e.g., [56]), a step-wise mutation model [77] or its extended versions (e.g., [78,79]). In principle, model misspecification could lead to erroneous results, so how to assign a correct mutation model to each locus would be an important issue to study. Nevertheless, as long as the locus has only two states, or if its multiple states can be classified into two broad categories under some objective criteria, the results of our study should hold.

### Relationship with background selection

The words “deleterious recurrent mutations” may be reminiscent of background selection, whereby deleterious mutations on a nearly non-recombining genomic region reduce the regional effective population size and thus reduce the regional genetic variability as compared to a freely recombining region (e.g., [73,80-83]). This mechanism could be related to our new neutrality tests in at least two different ways: first as a potential subject of our new tests, and second as a potential noise hampering our tests. These aspects will be discussed in some details in *Supplementary discussion* in Additional file 1. Recently, some complications on background selection have been revealed (e.g., [84,85]). To fully understand how our new tests will be impacted by background selection, or more generally the Hill-Robertson interference [86], we will need further studies using simulated data (e.g., [84]) and possibly data on *Drosophila* genomes (e.g., [85,87-89]).

### Comparing the definitions of our test statistics to those of traditional tests

One of our test statistic, *Max*^*D*^|_*M*_, is somewhat reminiscent of the statistic for Ewens’ test, which is the frequency of the most common haplotype conditional on the number of haplotypes in the sample (*K*). The other test statistic, *Tot*^*D*^|_*M*_, could be regarded as an analog of the statistic for the EW test, which is the haplotype homozygosity conditional on *K*. Despite the similarities, whereas the traditional tests detected negative selection on recurrent mutations at rates that are at best marginally better than that obtained by chance, our new tests detected negative selection at quite high rates. What causes this difference?

One big difference between the two groups of tests is that our tests only count mutant alleles with mutations whose effects we wish to examine, such as structural variations, while traditional tests count *all* haplotypes including those not bearing the mutations of interest. Because deleterious mutants in general account for only a minority among sampled sequences, haplotypes not bearing the mutation of interest determine the major behaviors of the traditional test statistics, which obscures the signals of the deleterious mutations. In contrast, our test statistics, *Max*^*D*^|_*M*_ and *Tot*^*D*^|_*M*_, only contain information on the mutation of interest. Therefore, they are unlikely to be disturbed by stochastic fluctuations affecting other haplotypes.

For theoretical studies of the new tests, it might be better to have analytical formulae for the null-distributions. Given the aforementioned similarity between our tests and Ewens’ and the EW tests, such formulae may be derivable at least under a constant-size population, by modifying the derivation of the Ewens sampling formula [16,17] and/or following a path similar to but slightly different from that to the equations (8) and (11) in [90]. The formulas in [90] were derived under the modified infinite-alleles model with two classes of alleles, one selectively neutral, the other deleterious [91,92]. It should be noted that past studies [91,92] focused on formulas under a fixed number of sampled deleterious mutants. What we need here, however, are *null*-distributions, which must be derived under a fixed *total* number of randomly sampled sequences, including *both* classes of alleles, and under the selective neutrality of both classes. (Also, mutations must be turned off between alleles in the same class.) Once analytical null-distributions are derived under such a neutral two-class model, we will be able to define yet another new statistical test similar to Slatkin’s exact test [9,10], by using the full configuration, (*D*_*1*_,*D*_*2*_,…,*D*_*M*_), of the numbers of sampled mutants resulting from identified forward mutations. Such an “exact test” might be slightly more powerful than the two tests proposed in this paper, because it can partition the sample space more finely. Once derived, the null-distributions may be relatively easily extended to an expanding population, whose effects were also examined in [90].

### Extended application of our new neutrality tests

In this paper, we mainly examined the performance of our new neutrality tests applied to recurrent mutation on a simple SV locus. However, as briefly explained in the *Background*, our new tests could possibly be applicable to other types of recurrent mutations as long as they satisfy two conditions: (i) the subject mutations share some features clearly distinguishable from other, mostly neutral mutations; and (ii) sequences with subject mutations can be sub-classified at least approximately into classes of shared origins by some means, such as a sequence genealogy. As a third kind of subject, we mentioned a class of sites that are lumped together according to putative signs of functional loss or impairment of a gene locus.

For example, phenylketonuria is a disease caused by hundreds of types of disabling or malfunctioning mutations on the phenylalanine hydroxylase (PAH) gene (reviewed e.g., in [93,94]). Our new tests are likely to detect (or rediscover) such diseases (see Additional file 1) and, by analogy, the tests are also expected to detect purifying selection operating on putative genes with unknown functions. This might considerably extend the use of our new tests, because they may help identify cryptic diseases, or they could help validate putative genes that are automatically annotated *e.g*., by genome projects. To make sure that this is true, however, we need to further test their performance in realistic settings.

It should be noted that the sequence genealogy may not need be reconstructed when applying our new tests to this class of mutations, because different mutational origins are likely to be identified by the locations and characteristics of the mutations.

### Potential use of our new parsimony algorithm to enumerate mutation scenarios

As a requirement for our new tests, we developed a new parsimony algorithm that maps a minimum number of mutations on a genealogy while resolving incomplete phylogenetic relationships if necessary, given an incompletely resolved genealogy and current states of sequences at a recurrently mutating locus (Additional file 2). The algorithm is a modified extension of Sankoff’s parsimony algorithm [61] to a multifurcated phylogenetic tree. Although we invented the algorithm in order to define the *Max*^*D*^|_*M*_ and *Tot*^*D*^|_*M*_ test statistics, the algorithm may actually find wider applications. For example, it may be extended to infer a finely resolved gene genealogy by combining fast-evolving characters, such as micro-satellite polymorphisms, with slow-evolving characters, such as SNPs in a linked region.

## Conclusions

Detecting selection on mutants has been a crucial goal of population and medical genetics. However, it has been very difficult to identify negatively selected (deleterious) mutants via purely population genetics methods, mostly because deleterious mutants leave only weak molecular signals that are very difficult to detect. We came up with the novel idea of synergizing the signals left by recurrent mutation events on gene genealogy, and devised two statistics, *Max*^*D*^|_*M*_ and *Tot*^*D*^|_*M*_, to detect negative selection on recurrent mutations at a subject locus. Our simulation analyses demonstrated that the neutrality tests based on these two statistics have high powers to detect negative selection under a constant-size population, and moderate powers under expanding populations. The next task will be to examine whether these methods also work under more realistic population genetics conditions, by including such factors as recombination and population substructure. Our new neutrality tests can be used with segmental mutations, such as genome structural variations and microsatellite mutations, data on which are expected to increase steadily as experimental technologies continue to advance. Our tests open new venues for studying the population genetics of recurrent mutations, and may become useful in molecular medicine by identifying genomic disorders that may have escaped identification by currently existing methods. Most of the scripts and Perl modules used in this study, including the Perl module implementing our new parsimony algorithm to enumerate mutation scenarios, are packaged in their original forms into Additional file 3 (a ZIP archive).

## Abbreviations

CNV: Copy number variation; EW test: Ewens-Watterson test; SNP: Single nucleotide polymorphism; SV: Structural variation.

## Competing interests

The authors declare no competing interests.

## Authors’ contributions

KE conceived of using gene genealogy to detect negative selection on recurrent mutations. He also designed the study, implemented necessary algorithms, performed simulation data analyses, and drafted and revised the manuscript. GL and DG helped with the design of the study, the interpretation of the data, and with the drafting and revision of the manuscript. All authors read and approved the final manuscript.

## Supplementary Material

Additional file 1 **Supplementary notes, which consist of Supplementary methods, Supplementary discussion, Tables S1- S16, and Figures S1-S5**. Click here for file

Additional file 2 Detailed descriptions of our new parsimony algorithm to enumerate parsimonious mutation scenarios on an incompletely resolved genealogy. Click here for file

Additional file 3 **A ZIP archive that contains the original versions of a Perl module implementing our new parsimony algorithm, as well as of Perl and Bourne shell scripts used for our simulation data analyses to examine the performances of various neutrality tests including our two new tests.** It also contains a README file that describes how to use the modules and scripts. (The modules and scripts will run on a Mac OS X terminal. And they should probably run on other UNIX platforms as well, although we have not tested whether they indeed will.) The latest version of the modules and scripts, as well as some null-distributions, can be found in the DENSERM directory at the FTP repository of the Bioinformatics Organization [95]. Click here for file
