# Supplementary material for: Detecting negative selection on recurrent mutations using gene genealogy
Source: BMC Genet. 2013 May 7;14:37. doi: 10.1186/1471-2156-14-37 (PMC3661350; doi:10.1186/1471-2156-14-37)

Additional File 1:

SUPPLEMENTARY NOTES for

"Detecting negative selection on recurrent mutations using gene genealogy"

by Kiyoshi Ezawa, Giddy Landan, and Dan Graur

Table of Contents:

- Supplementary methods pp. 2-6
- Supplementary discussion pp. 6-8
- References cited pp. 8-10
- Supplementary tables pp. 11-26
- Supplementary figures pp. 27-33

Supplementary methods

Inferring gene genealogies and mutation scenarios (rationale)

The genealogy among the sequences in each simulated sample was inferred via the Neighbor-Joining (NJ) method [1] using the number of SNP sites at which the two sequences differ as a pairwise distance between the sequences. We expect that, in most real-life data, linked SNPs *alone* cannot completely resolve the genealogical relationships (Supplementary figure S1 A), leaving many multi-furcated nodes (Supplementary figure S1 B), because the number of such SNPs will be limited to around 50 – 100 at best (e.g. [2, 3]). However, most of the existing tree reconstruction algorithms, including the NJ method used in this study, are designed to construct a completely resolved tree even in such a case, by arbitrarily resolving multi-furcated nodes in an implementation-dependent manner (Supplementary figure S1 C). Such an arbitrarily resolved tree could often cause an overestimate of the number of mutation events, by splitting a cluster of identical-by-descent states with (an) erroneous interior branch(es) (compare panels D and E in Supplementary figure S1). Thus, from each inferred NJ tree, we first removed interior branches not supported by any SNP sites, and reconstructed an incompletely resolved genealogy, or a "SNP-supported tree" (Supplementary figure S1 F). Then, the root was placed at the mid-point between the pair of sequences with the largest distance.

Onto the "SNP-supported tree" thus constructed, we mapped mutation events at the recurrently mutating locus via a maximum parsimony principle. However, traditional parsimony algorithms (e.g. [4, 5]) could still overestimate the number of mutation events if some of them occur along unresolved branches (compare panels D and G in Supplementary figure S1). Because an overestimated number of mutations could cause false-positives in our new tests, we devised a new parsimony algorithm that errs on a conservative side by adding interior branches, if necessary, to put descendant nodes with the same state under an additional node (Supplementary figure S1 H). The algorithm tends to underestimate the number of mutation events if it does not correctly count the events, conforming to the defining philosophy of parsimony algorithms. The algorithm also enumerates all possible mutation scenarios that could result in the minimum number of mutations, each accompanied by additional interior branches necessary to realize the scenario. Details of this new algorithm, including the complexities that we encounter in actual analyses and how they are solved, are described in *Additional File 2*.

Empirical null-distributions for our new tests

We constructed null-distributions for our new tests empirically from the frequencies of the values of test statistics, $Max^D|_M$ and $Tot^D|_M$, exhibited by the sequence sets simulated under selective neutrality. When a sequence set has P_S (>0) parsimonious scenarios, each parsimonious scenario was assigned a weight factor of $w(scenario) = 1/P_S$, as well as the test statistics $Max^D|_M$ and $Tot^D|_M$. Then, under a fixed combination, (n, θ_μ, ν/μ, $\sigma = 0$), the empirical null-distribution $P_0^E[...]$ for $Max^D|_M$ was estimated as:

$$P_0^E[Max^D|_M = x \mid n,\theta_\mu,\nu/\mu,\sigma=0] = \frac{P_0^E[M\ forward\ mutations, Max^D = x \mid n,\theta_\mu,\nu/\mu,\sigma=0]}{P_0^E[M\ forward\ mutations \mid n,\theta_\mu,\nu/\mu,\sigma=0]}, \quad \text{(S1a)}$$

and $P_0^E[...]$ for $Tot^D|_M$ as:

$$P_0^E[Tot^D|_M = x \mid n,\theta_\mu,\nu/\mu,\sigma=0] = \frac{P_0^E[M\ forward\ mutations, Tot^D = x \mid n,\theta_\mu,\nu/\mu,\sigma=0]}{P_0^E[M\ forward\ mutations \mid n,\theta_\mu,\nu/\mu,\sigma=0]}. \quad \text{(S1b)}$$

In both equations, the following definition (for any outcome X) was used:

$$P_0^E[X \mid n,\theta_\mu,\nu/\mu,\sigma=0] \equiv \frac{\left.\left(\sum_{Parsimonious\ scenarios\ satisfying\ X} w(scenario)\right)\right|_{(n,\theta_\mu,\nu/\mu,\sigma=0)}}{\left.\left(\sum_{All\ parsimonious\ scenarios.} w(scenario)\right)\right|_{(n,\theta_\mu,\nu/\mu,\sigma=0)}}. \quad \text{(S1c)}$$

On the right hand side, the subscript following the vertical bar represents the variables that are fixed. Whether the data are real or simulated, we know the sample size, n, in advance. In reality, however, it is difficult to know *a priori* the mutation rate, θ_μ, for each recurrently mutating locus without additional information. Besides, although the backward/forward ratio, ν/μ, could be inferred from the type of recurrent mutations, such an inference may not necessarily be precise enough. To account for such uncertainties, we dealt with the two parameters as follows. First, θ_μ was assumed to be power law distributed:

$$P[\theta_\mu > X] = A \cdot X^{-\alpha}. \quad \text{(S2)}$$

Here the exponent α dictates the power-law behavior, and A is a normalization factor. This distribution is theoretically expected, because the size of some SVs such as insertions/deletions are known to be power-law distributed (e.g., [6] and references therein), and because the time-frequencies of events are also known to follow the power-law function of the events' sizes (e.g., [7]). Results of recent data analyses on CNV frequencies (e.g., [8, 9]) also seem to support the power-law distribution. We used α = 0.5, 1, and 2 to cover the current knowledge from data

analyses. Next, following a common practice, we regarded these values of $\theta_\mu = 10^{-1}, 10^{-1/2}, 1\,(=10^0), 10^{+1/2}, 10^{+1}$ as the mid-point representatives of the equi-spaced intervals (in the logarithmic scale), $10^{-3/2} \le \theta_\mu < 10^{-3/4}$, $10^{-3/4} \le \theta_\mu < 10^{-1/4}$, $10^{-1/4} \le \theta_\mu < 10^{+1/4}$, $10^{+1/4} \le \theta_\mu < 10^{+3/4}$, $10^{+3/4} \le \theta_\mu < 10^{+3/2}$, respectively. Thus we assigned the relative probability, $P[\theta_\mu \mid \alpha]$, of each of $\theta_\mu = 10^{-1}, 10^{-1/2}, 1\,(=10^0), 10^{+1/2}, 10^{+1}$ under a fixed exponent α as shown in Supplementary table S1. Then, under a fixed combination of n, α, ν/μ, and $\sigma = 0$, we estimated the empirical null-distribution for $Max^D|_M$ as:

$$P_0^E[Max^D|_M = x \mid n,\alpha,\nu/\mu,\sigma=0] = \frac{P_0^E[M\ forward\ mutations, Max^D = x \mid n,\alpha,\nu/\mu,\sigma=0]}{P_0^E[M\ forward\ mutations \mid n,\alpha,\nu/\mu,\sigma=0]}\ , \quad \text{(S3a)}$$

and that for $Tot^D|_M$ as:

$$P_0^E[Tot^D|_M = x \mid n,\alpha,\nu/\mu,\sigma=0] = \frac{P_0^E[M\ forward\ mutations, Tot^D = x \mid n,\alpha,\nu/\mu,\sigma=0]}{P_0^E[M\ forward\ mutations \mid n,\alpha,\nu/\mu,\sigma=0]}\ . \quad \text{(S3b)}$$

On the right-hand sides of both equations, the following definition (for any outcome X) was used:

$$P_0^E[X \mid n,\alpha,\nu/\mu,\sigma=0] \equiv \sum_{\theta_\mu = 10^{-1},10^{-1/2},10^0,10^{+1/2},10^{+1}} P_0^E[X \mid n,\theta_\mu,\nu/\mu,\sigma=0] \cdot P[\theta\mu \mid \alpha]\ . \quad \text{(S3c)}$$

The above equations (S3a-c) still assume that ν/μ is known. We also defined the null-distributions when ν/μ is unknown. The distribution for $Max^D|_M$ is given as:

$$P_0^E[Max^D|_M = x \mid n,\alpha,--,\sigma=0] = \frac{P_0^E[M\ forward\ mutations, Max^D = x \mid n,\alpha,--,\sigma=0]}{P_0^E[M\ forward\ mutations \mid n,\alpha,--,\sigma=0]}\ . \quad \text{(S4a)}$$

The distribution for $Tot^D|_M$ is:

$$P_0^E[Tot^D|_M = x \mid n,\alpha,--,\sigma=0] = \frac{P_0^E[M\ forward\ mutations, Tot^D = x \mid n,\alpha,--,\sigma=0]}{P_0^E[M\ forward\ mutations \mid n,\alpha,--,\sigma=0]}\ . \quad \text{(S4b)}$$

The right-hand sides of the above two equations use the following definition (for any outcome X):

$$P_0^E[X \mid n,\alpha,--,\sigma=0] \equiv \frac{1}{5}\left(\sum_{\nu/\mu = 0,\frac{1}{2},1,2,3} P_0^E[X \mid n,\alpha,\nu/\mu,\sigma=0]\right)\ . \quad \text{(S4c)}$$

Based on the equations (S1a,b), (S3a,b), and (S4a,b), the empirical cumulative distributions are calculated as:

$$P_0^E[Max^D|_M \le x \mid Y] = \sum_{z \le x} P_0^E[Max^D|_M = z \mid Y] \quad \text{(S5a)}$$

for $Max^D|_M$, and

$$P_0^E[Tot^D|_M \le x \mid Y] = \sum_{z \le x} P_0^E[Tot^D|_M = z \mid Y] \qquad \text{(S5b)}$$

for $Tot^D|_M$. In the above equations, Y is a condition specified, either $(n,\ \theta_\mu,\ \nu/\mu,\ \sigma=0)$, $(n,\ \alpha,\ \nu/\mu,\ \sigma=0)$, or $(n,\ \alpha, \text{--},\ \sigma=0)$ (i.e., with the ratio ν/μ unknown).

Other neutrality tests

To compare with our new tests, we also conducted three traditional neutrality tests, Tajima's D test [10], Ewens' test [11], and the Ewens-Watterson test [12]. They were conducted in similar manners as our tests, using the empirical null-distributions as described in the last subsection and in the main *Methods*, with $Max^D|_M$ or $Tot^D|_M$ replaced with appropriate test statistics. One big difference is that the empirical P-values for the traditional tests were defined directly with the sequence set, instead of being mediated by parsimonious scenarios as for our tests, because they are not necessary when defining the test statistics other than $Max^D|_M$ and $Tot^D|_M$. Here we will briefly recall the definitions of the test statistics used.

Tajima's D statistic [10] is defined as:

$$D = \frac{\pi - S/a_1}{\sqrt{\hat{V}ar[\pi - S/a_1]}},$$

where π is the average of the pairwise sequence distances between sampled sequences, S is the number of segregating sites (= 50 in this study), $a_1 \equiv \sum_{i=1}^{n-1} \frac{1}{i}$ is a constant, and

$$\hat{V}ar[\pi - S/a_1] = e_1 S + e_2 S(S-1)$$

is the estimator of the variance of $\pi - S/a_1$. The coefficients in the definition are

$$e_1 = \frac{1}{a_1}\left(\frac{n+1}{3(n-1)} - \frac{1}{a_1}\right) \quad \text{and} \quad e_2 = \frac{1}{a_1^2 + a_2}\left(\frac{2(n^2+n+3)}{9n(n-1)} - \frac{n+2}{na_1} + \frac{a_2}{a_1^2}\right),$$

with $a_2 \equiv \sum_{i=1}^{n-1} \frac{1}{i^2}$. Because the D statistic tends to be negative under negative selection, we defined the P-value in terms of a lower-tailed cumulative distribution under a null-hypothesis Y, as

$$P^E(\textit{sequence set with } D = x^{Obs}) \equiv P_0^E[D \le \overline{x}^{Obs} \mid Y].$$

Ewens' test [11] uses the test statistic, $Max^H|_K$, which is the copy number (i.e., frequency) of the commonest haplotype (Max^H), tested *conditionally on* the number of different

haplotypes in the sample (K). This statistic is expected to be larger under negative selection than under neutrality. Thus the empirical P-value is defined using an upper-tailed cumulative distribution:

$$P^{E}(\textit{sequence set with } Max^{H}|_{K} = x^{Obs}) \equiv P_0^{E}[Max^{H}|_{K} \geq \underline{x}^{Obs} \mid Y] \ ,$$

where $\underline{x}^{Obs}$ is the maximum value of $Max^{H}|_{K}$ in the null distribution not more than x^{Obs}.

The Ewens-Watterson (EW) test [12] uses the test statistic, $F^{H}|_{K}$, which is the haploid homozygosity:

$$F^{H} \equiv \frac{1}{n^2}\sum_{i=1}^{K} f_i^2 \quad ,$$

with f_i being the sample copy number of the i th haplotype, again tested *conditionally on* K. This statistics is also expected to be larger under negative selection than under neutrality, therefore the empirical P-value is defined as that for Ewens' test, with $Max^{H}|_{K}$ replaced by $F^{H}|_{K}$. After conducting the tests, however, we noticed that the detection rate was lower than the nominal false-positive rate in general. Therefore, we also examined the lower-tailed P-value, which turned out to perform better than the upper-tailed one. Thus we will also show the test results based on the lower-tailed P-value.

Supplementary discussion

Relationship with background selection

The words "deleterious recurrent mutations" may be reminiscent of background selection, whereby deleterious mutations on a (nearly) non-recombining genomic region reduce the regional effective population size and thus reduce the regional genetic variability as compared to that in a freely recombining region (*e.g.*, [13-17]). This mechanism could be related to our new neutrality tests at least in two ways: first as a potential subject of our new tests, and second as a potential noise hampering our tests.

Let us first consider whether our new tests can detect (a) "culprit(s)" of backward selection. According to the results of our performance tests, our new tests were good at detecting recurrent deleterious mutations with $|\sigma| (= |4Ns|) \geq 100$ and $1 \leq \theta_{\mu} (= 4N\mu) < 10$. According to [13], such a locus reduces the effective population size of a linked neutral locus to approximately

$\exp\left(\frac{\theta_\mu}{\sigma}\right)$-times that of a free neutral locus. For a locus of recurrent deleterious mutation detectable by our new tests, the multiplicative factor is at least $\exp(-1/10) \approx 0.905$, which is quite close to unity. Thus the effect of a single locus with detectable recurrent deleterious mutation is quite small. Therefore, it may be difficult to detect a *single* "culprit" of background selection with a substantial effect. Nevertheless, if there are many (maybe dozens of) such loci in a (nearly) non-recombining region, the combined effect of such loci may be substantial. Such a situation may be similar to that of the third class of our subjects, with a much bigger θ_μ (maybe about 100 or more). But, as we will see in the following, selection coefficient $\sigma(=4Ns)$ will have to be much bigger than 100 in magnitude, for the subject locus to be detectable.

Second, let us consider how background selection could hamper the detection of negative selection on a subject locus undergoing recurrent mutations. The primary effect of background selection is to reduce the regional effective population size. This will raise the raw mutation rate (μ) and the absolute value of the raw selection coefficient ($|s|$) for the recurrent deleterious mutation to be detectable. This effect can also be re-interpreted as the size reduction of the gene genealogy. When most background loci are under weak or moderate selection, the neutral allele frequency spectrum is known to skew toward rare alleles (*e.g.*, [13]). Because significant background selection occurs almost exclusively on nearly non-recombining regions, the null-distributions of our new tests could incorporate the effect of such background selection if we input the spectrum of neutral SNPs in that region. The situation will be similar to that of an expanding population, under which our new tests showed some success. Thus, even with the noise of background selection, our tests may detect deleterious recurrent mutation at a particular locus provided that it is frequent enough and selected against strongly enough. Moreover, the subject mutation will be distinguished from the background mutation according to their locations. A sample should rarely contain a sequence bearing both the subject mutation and a background mutation if both mutations are strongly selected against. Thus we will be able to determine whether the subject mutation is under strong selection or not, provided that the mutation occurs frequently enough. Recently, some complications on background selection have been revealed (e.g., [18, 19]). To fully understand the effects of background selection, or more generally the Hill-Robertson inteference ([20]), on our new tests, we will need further studies, using simulated data (e.g., [18]) and possibly data on *Drosophila* genomes (e.g., [19, 21-23]).

Phenylteonuria: a possible subject of our new neutrality tests

In the main introduction and in the main discussion, we argued that a third kind of subject of our new tests is a class of reasonably linked loss-of-function mutations on a gene locus. And we named phenylketonuria as a pedagogical (but not necessarily practical) example. Here let us elaborate on this.

Phenylketonuria is a disease caused by hundreds of types of disabling or malfunctioning mutations on the phenylalanine hydroxylase (PAH) gene (reviewed e.g., in [24, 25]). In the European population, its average prevalence is about one case per 10,000 livebirths [26]. Because this disease is autosomal recessive, we can expect to find, on average, two disease-associated mutants out of 200 sampled European sequences of the PAH gene. Moreover, because this disease usually occurs as "composite heterozygous" consisting of two different types of disease-associated alleles (see e.g., [24], or Online Mendelian Inheritance in Man (OMIM) [27]), disease-associated mutants in a sample will likely be of different origins. Therefore, if applied under a proper null mutation model, our new neutrality tests have high chances to detect negative selection on disabling mutations of the PAH gene. Although phenylketonuria is already world-famous, our new tests could be applied also to a set of disabling mutations of a gene that has not been examined well so far. For example, there are thousands of automatically annotated genes whose functions are unknown. Our tests could help evaluate the functional importance of such genes. Such analyses may also uncover cryptic genetic disorders that have escaped identification by established methods so far.

It should be worth a mention that we may not need to reconstruct a gene genealogy when applying our tests to this class of mutations, because we could tell whether the mutations share their origins or not just from their locations and/or their characteristics.

References cited

1. Saitou N, Nei M: **The neighbor-joining method: A new method for reconstructing phylogenetic trees.** *Mol. Biol. Evol* 1987, **4**:406-425.
2. The International HapMap Consortium: **A haplotype map of the human genome.** *Nature* 2005, **437**:1299-1320.
3. The International HapMap 3 Consortium: **Integrating common and rare genetic variation in diverse human populations.** *Nature* 2010, **467**:52-58.

4. Fitch WM: **Toward defining the course of evolution: Minimum change for a specified tree topology.** *Syst Zool* 1971, **20**:406-416.
5. Sankoff D: **Minimal mutation trees of sequences.** *SIAM J Appl Math* 1975, **28**:35-42.
6. Cartwright R: **Problems and solutions for estimating indel rates and length distributions.** *Mol Biol Evol* 2009, **26**:473-480.
7. Solé RV, Manrubia SC, Benton M, Kauffman S, Bak P: **Criticality and scaling in evolutionary ecology.** *Trends Ecol Evol* 1999, **14**:156-160.
8. Conrad DF, Pinto D, Redon R, Feuk L, Gokcumen O, Zhang Y, Aerts J, Andrews TD, Barnes C, Campbell P, Fitzgerald T, Hu M, Ihm CH, Kristiansson K, MacArthur DG, MacDonald JR, Onyiah I, Pang AWC, Robson S, Stirrups K, Valsesia A, Walter K, W J, Wellcome Trust Case Control Consortium, Tyler-Smith C, Carter NP, Lee C, Scherer SW, Hurles ME: **Origins and functional impact of copy number variation in the human genome.** *Nature* 2010, **464**:704-712.
9. Fu W, Zhang F, Wang Y, Gu X, Jin L: **Identification of copy number variation hotspots in human populations.** *Am J Hum Genet* 2010, **87**:494-504.
10. Tajima F: **Statistical model for testing the neutral mutation hypothesis by DNA polymorphism.** *Genetics* 1989, **123**:585-595.
11. Ewens W J: **Testing for increased mutation rate for neutral alleles.** *Theor Popul Biol* 1973, **4**:251-258.
12. Watterson GA: **The homozygosity test of neutrality.** *Genetics* 1978, **88**:405-417.
13. Charlesworth B, Morgan MT, Charlesworth D: **The effect of deleterious mutations on neutral molecular variation.** *Genetics* 1993, **134**:1289-1303.
14. Hudson RR: **How can the low levels of DNA sequence variation in regions of the Drosophila genome with low recombination rates explained?** *Proc. Natl. Acad. Sci USA* 1994, **91**:6815-6818.
15. Charlesworth D, Charlesworth B, Morgan MT: **The pattern of neutral molecular variation under the background selection model.** *Genetics* 1995, **141**:1619-1632.
16. Hudson RR, Kaplan NL: **Coalescent process and background selection.** *Phil Transac Biol Sci* 1995, **349**:19-23.
17. Nordborg M, Charlesworth B, Charlesworth D: **The effect of recombination on background selection.** *Genet. Res.* 1996, **67**:159-174.
18. Kaiser VB, Charlesworth B: **The effects of deleterious mutations on evolution in non-**

recombining genomes. *Trends Genetics* 2009, **25**:9-12.

19. Charlesworth B, Betancourt AJ, Kaiser VB, Gordo I: **Genetic recombination and molecular evolution.** *Cold Spring Harbor Symposia on Quantitative Biology* 2009, **74**:177-186.
20. Hill WG, Robertson A: **The effect of linkage on limits to artificial selection.** *Genet Res* 1966, **8**:269-294.
21. Arguello JR, Zhang Y, Kado T, Fan C, Zhao R, Innan H, Wang W, Long M: **Recombination yet inefficient selection along the Drosophila melanogaster subgroup's fourth chromosome.** *Mol Biol Evol* 2010, **27**:848-861.
22. Campos JL, Charlesworth B, Haddrill PR: **Molecular evolution in nonrecombining regions of the Drosoplhila melanogaster genome.** *Genome Biol Evol* 2012, **4**:278-288.
23. McGaugh SE, Hell CSS, Manzano-Winkler B, Loewe L, Goldstein S, Himmel TL, Noor MAF: **Recombination modulates how selection affects linked sites in Drosophila.** *PLoS Biol* 2012, **10**:e1001422.
24. Scriver CR: **The PAH gene, phenylketonuria, and a paradigm shift.** *Hum Mutat* 2007, **28**:831-845.
25. Blau N, van Spronsen FJ, Levy HL: **Phenylketonuria.** *Lancet* 2010, **376**:1417-1427.
26. Loeber JG: **Neonatal screening in Europe; the situation in 2004.** *J Inherit Metab Dis* 2007, **30**:430-438.
27. **Online Mendelian Inheritance in Man (OMIM)** [http://www.ncbi.nlm.nih.gov/omim]

Supplementary table S1. Weight factors assigned to the simulation-based null-probabilities with various forward mutation rates

NOTE. In each cell in the bulk is the weight factor (or relative probability), $P[\theta_\mu | \alpha]$, of a given forward mutation rate θ_μ ($\equiv 4N\mu$) under the power-law exponent α. See Eq. (S3c) in *Supplementary methods* for an example of how they are used.

θ_μ	$\alpha = 0.5$	$\alpha = 1$	$\alpha = 2$
10^{-1}	0.589	0.822	0.968
$10^{-1/2}$	0.188	0.122	0.028
$1(=10^0)$	0.106	0.038	0.0028
$10^{+1/2}$	0.059	0.012	0.00028
10^{+1}	0.058	0.0053	3.2×10^{-5}

Supplementary table S2. Accuracy of our new parsimony method compared to traditional tree reconstruction methods: dependence on n and ν/μ.

NOTE. The symbol n denotes the sample size, and ν/μ is the backward/forward rate ratio. In each cell, the upper number is the average number of false-positive branches per genealogy, and the lower number is the average "additional true-branch rate", $\frac{ATP}{ATP+FP}$, where ATP is the number of true-positive branches not supported by SNPs, and FP is the number of false-positive branches. The number in parentheses on the right in each cell is the standard deviation of the quantity whose average is on the left.

A. Traditional tree reconstruction algorithm (represented by NJ).

Sample size (n)	$\nu/\mu = 0$	$\nu/\mu = 1$	$\nu/\mu = 3$
50	28.57 (4.07)<br>0.120 (0.067)	28.63 (4.08)<br>0.118 (0.067)	28.56 (4.08)<br>0.119 (0.067)
100	72.70 (5.24)<br>0.071 (0.035)	72.78 (5.15)<br>0.071 (0.035)	72.85 (5.07)<br>0.070 (0.034)
200	167.53 (5.90)<br>0.040 (0.018)	167.58 (5.91)<br>0.040 (0.018)	167.66 (5.94)<br>0.040 (0.018)

B. Our new parsimony algorithm.

Sample size (n)	$\nu/\mu = 0$	$\nu/\mu = 1$	$\nu/\mu = 3$
50	0.37 (0.74)<br>0.449 (0.379)	0.70 (1.01)<br>0.392 (0.325)	0.62 (0.91)<br>0.400 (0.344)
100	0.59 (1.09)<br>0.444 (0.352)	1.06 (1.46)<br>0.378 (0.298)	1.02 (1.36)<br>0.383 (0.309)
200	0.87 (1.46)<br>0.428 (0.333)	1.44 (1.91)<br>0.368 (0.283)	1.47 (1.87)<br>0.363 (0.286)

Supplementary table S3. Accuracy of our new parsimony method compared to traditional tree reconstruction methods: dependence on θ_μ and σ.

NOTE. θ_μ and σ are the rescaled forward mutation rate and the rescaled selection coefficient, respectively. The results are shown for sets with the sample size $n = 100$ and the backward/forward rate ratio $\nu / \mu = 1$. The layout of the numbers in each cell is the same as that for Supplementary table S2.

A. Traditional tree reconstruction algorithm (represented by NJ).

θ_μ	$\sigma = 0$ (neutral)	$\sigma = -10$	$\sigma = -10^{3/2}$	$\sigma = -10^2$	$\sigma = -10^{5/2}$
10^{-1}	73.15 (5.14)<br>0.070 (0.034)	72.56 (4.88)<br>0.071 (0.036)	72.32 (4.86)<br>0.073 (0.036)	71.20 (5.24)<br>0.080 (0.036)	72.45 (4.58)<br>0.072 (0.036)
$10^{-1/2}$	73.06 (5.18)<br>0.070 (0.035)	72.34 (4.96)<br>0.073 (0.034)	73.16 (5.41)<br>0.070 (0.035)	73.25 (5.08)<br>0.068 (0.035)	72.24 (5.14)<br>0.071 (0.035)
$1 (= 10^0)$	72.54 (5.07)<br>0.072 (0.035)	72.50 (5.02)<br>0.071 (0.034)	72.27 (5.32)<br>0.071 (0.035)	72.89 (5.08)<br>0.070 (0.035)	72.27 (5.35)<br>0.073 (0.037)
$10^{+1/2}$	72.87 (5.15)<br>0.071 (0.034)	72.44 (5.10)<br>0.072 (0.034)	72.54 (5.16)<br>0.072 (0.035)	72.56 (4.98)<br>0.071 (0.034)	72.96 (5.16)<br>0.071 (0.035)
10^{+1}	72.80 (5.24)<br>0.070 (0.036)	72.61 (5.19)<br>0.071 (0.035)	72.70 (5.21)<br>0.070 (0.034)	72.93 (5.12)<br>0.069 (0.034)	72.46 (5.23)<br>0.073 (0.035)

B. Our new parsimony algorithm.

θ_μ	$\sigma = 0$ (neutral)	$\sigma = -10$	$\sigma = -10^{3/2}$	$\sigma = -10^2$	$\sigma = -10^{5/2}$
10^{-1}	0.06 (0.22)<br>0.838 (0.331)	0.02 (0.14)<br>0.939 (0.225)	0.03 (0.17)<br>0.931 (0.230)	0.00 (0.00)<br>1.00 (0.00)	0.00 (0.00)<br>1.00 (0.00)
$10^{-1/2}$	0.20 (0.40)<br>0.663 (0.386)	0.10 (0.31)<br>0.797 (0.351)	0.04 (0.20)<br>0.898 (0.288)	0.03 (0.15)<br>0.917 (0.244)	0.00 (0.00)<br>1.00 (0.00)
$1 (= 10^0)$	0.76 (0.73)<br>0.475 (0.329)	0.46 (0.62)<br>0.586 (0.366)	0.22 (0.45)<br>0.694 (0.371)	0.08 (0.28)<br>0.802 (0.356)	0.02 (0.12)<br>0.889 (0.284)
$10^{+1/2}$	2.20 (1.01)<br>0.327 (0.209)	1.83 (1.06)<br>0.362 (0.243)	0.99 (0.94)<br>0.451 (0.324)	0.35 (0.56)<br>0.548 (0.409)	0.13 (0.35)<br>0.489 (0.460)
10^{+1}	4.29 (1.20)<br>0.243 (0.139)	4.06 (1.20)<br>0.251 (0.153)	3.38 (1.28)<br>0.265 (0.173)	1.73 (1.14)<br>0.300 (0.258)	0.48 (0.67)<br>0.340 (0.388)

Supplementary table S4. Relative frequencies of recurrent mutations captured by gene genealogy, out of polymorphic loci

NOTE. The results are shown for sets *with* $n = 50$ sampled sequences each. ν / μ is the backward/forward ratio of mutation rates. θ_μ ($\equiv 4N\mu$) is the rescaled forward mutation rate. σ ($\equiv 4Ns$) denotes the rescaled selection coefficient.

[a] "NA" is assigned to a category with less than 30 polymorphic loci.

A. $\nu / \mu = 0$ **.**

θ_μ	$\sigma = 0$ (neutral)	$\sigma = -10$	$\sigma = -10^{3/2}$	$\sigma = -10^{2}$	$\sigma = -10^{5/2}$
10^{-1}	0.070	0.105	0.026	0.030	NA
$10^{-1/2}$	0.178	0.184	0.109	0.029	0.063
$1(=10^{0})$	0.345	0.511	0.318	0.174	0.064
$10^{+1/2}$	NA[a]	0.797	0.791	0.407	0.168
10^{+1}	NA	NA	0.991	0.886	0.476

B. $\nu / \mu = 1$ **.**

θ_μ	$\sigma = 0$ (neutral)	$\sigma = -10$	$\sigma = -10^{3/2}$	$\sigma = -10^{2}$	$\sigma = -10^{5/2}$
10^{-1}	0.073	0.064	0.063	0.000	NA
$10^{-1/2}$	0.249	0.178	0.139	0.070	0.026
$1(=10^{0})$	0.629	0.525	0.337	0.134	0.043
$10^{+1/2}$	0.958	0.897	0.752	0.410	0.160
10^{+1}	0.999	0.998	0.986	0.861	0.479

C. $\nu / \mu = 3$ **.**

θ_μ	$\sigma = 0$ (neutral)	$\sigma = -10$	$\sigma = -10^{3/2}$	$\sigma = -10^{2}$	$\sigma = -10^{5/2}$
10^{-1}	0.133	0.038	0.023	0.027	NA
$10^{-1/2}$	0.356	0.179	0.092	0.047	0.000
$1(=10^{0})$	0.700	0.494	0.343	0.115	0.039
$10^{+1/2}$	0.939	0.866	0.695	0.388	0.173
10^{+1}	0.992	0.983	0.951	0.810	0.465

Supplementary table S5. Relative frequencies of recurrent mutations captured by gene genealogy, out of polymorphic loci

NOTE. The results are shown for sets *with* $n = 200$ sampled sequences each. ν / μ is the backward/forward ratio of mutation rates. θ_μ ($\equiv 4N\mu$) is the rescaled forward mutation rate. σ ($\equiv 4Ns$) denotes the rescaled selection coefficient.

[a] "NA" is assigned to a category with less than 30 polymorphic loci.

A. $\nu / \mu = 0$ **.**

θ_μ	$\sigma = 0$ (neutral)	$\sigma = -10$	$\sigma = -10^{3/2}$	$\sigma = -10^2$	$\sigma = -10^{5/2}$
10^{-1}	0.109	0.086	0.059	0.049	0.020
$10^{-1/2}$	0.293	0.278	0.212	0.144	0.070
$1(=10^0)$	0.471	0.735	0.553	0.416	0.190
$10^{+1/2}$	NA[a]	0.906	0.959	0.832	0.473
10^{+1}	NA	NA	0.998	0.999	0.945

B. $\nu / \mu = 1$ **.**

θ_μ	$\sigma = 0$ (neutral)	$\sigma = -10$	$\sigma = -10^{3/2}$	$\sigma = -10^2$	$\sigma = -10^{5/2}$
10^{-1}	0.124	0.115	0.060	0.047	0.000
$10^{-1/2}$	0.388	0.292	0.218	0.119	0.042
$1(=10^0)$	0.822	0.747	0.607	0.367	0.206
$10^{+1/2}$	0.997	0.992	0.966	0.808	0.475
10^{+1}	1.000	0.998	1.000	0.998	0.922

C. $\nu / \mu = 3$ **.**

θ_μ	$\sigma = 0$ (neutral)	$\sigma = -10$	$\sigma = -10^{3/2}$	$\sigma = -10^2$	$\sigma = -10^{5/2}$
10^{-1}	0.222	0.115	0.097	0.098	0.048
$10^{-1/2}$	0.507	0.308	0.221	0.117	0.078
$1(=10^0)$	0.877	0.748	0.578	0.348	0.164
$10^{+1/2}$	0.994	0.988	0.947	0.801	0.501
10^{+1}	1.000	1.000	1.000	0.996	0.929

Supplementary table S6. False positive and true positive rates via $Tot^D|_M$, when ν/μ is *not* known in advance

NOTE. Here, $n = 100$ and α = ***0.5***, as well as 5% nominal significance level, are fixed. The tables show proportions of loci that tested positive via $Tot^D|_M$ out of those whose gene genealogies revealed recurrent mutations. ν/μ is the backward/forward ratio of mutation rates. θ_μ ($\equiv 4N\mu$) is the rescaled forward mutation rate. σ ($\equiv 4Ns$) denotes the rescaled selection coefficient.

[a] "NA" is assigned to a category with less than 10 loci with revealed recurrent mutations.

A. $\nu/\mu = 0$ **.**

θ_μ	$\sigma = 0$ (neutral)	$\sigma = -10$	$\sigma = -10^{3/2}$	$\sigma = -10^2$	$\sigma = -10^{5/2}$
10^{-1}	0.058	0.063	0.182	NA	NA
$10^{-1/2}$	0.065	0.060	0.177	0.333	NA
$1(=10^0)$	0.005	0.113	0.324	0.537	0.615
$10^{+1/2}$	NA[a]	0.024	0.239	0.575	0.774
10^{+1}	NA	NA	0.012	0.525	0.714

B. $\nu/\mu = 1$ **.**

θ_μ	$\sigma = 0$ (neutral)	$\sigma = -10$	$\sigma = -10^{3/2}$	$\sigma = -10^2$	$\sigma = -10^{5/2}$
10^{-1}	0.057	0.063	NA	NA	NA
$10^{-1/2}$	0.044	0.208	0.203	0.625	NA
$1(=10^0)$	0.033	0.130	0.368	0.533	0.739
$10^{+1/2}$	0.011	0.075	0.304	0.524	0.706
10^{+1}	0.000	0.002	0.090	0.578	0.749

C. $\nu/\mu = 3$ **.**

θ_μ	$\sigma = 0$ (neutral)	$\sigma = -10$	$\sigma = -10^{3/2}$	$\sigma = -10^2$	$\sigma = -10^{5/2}$
10^{-1}	0.054	0.000	NA	NA	NA
$10^{-1/2}$	0.040	0.143	0.351	0.500	NA
$1(=10^0)$	0.046	0.150	0.269	0.537	0.815
$10^{+1/2}$	0.062	0.160	0.343	0.583	0.756
10^{+1}	0.085	0.158	0.324	0.629	0.744

Supplementary table S7. False positive and true positive rates via $Tot^D|_M$, when ν/μ is *not* known in advance

NOTE. Here, $n=100$ and α = ***2.0***, as well as 5% nominal significance level, are fixed. The tables show proportions of loci that tested positive via $Tot^D|_M$ out of those whose gene genealogies revealed recurrent mutations. ν/μ is the backward/forward ratio of mutation rates. θ_μ ($\equiv 4N\mu$) is the rescaled forward mutation rate. σ ($\equiv 4Ns$) denotes the rescaled selection coefficient.

[a] "NA" is assigned to a category with less than 10 loci with revealed recurrent mutations.

A. $\nu/\mu=0$ **.**

θ_μ	$\sigma=0$ (neutral)	$\sigma=-10$	$\sigma=-10^{3/2}$	$\sigma=-10^2$	$\sigma=-10^{5/2}$
10^{-1}	0.048	0.063	0.182	NA	NA
$10^{-1/2}$	0.055	0.052	0.162	0.333	NA
$1(=10^0)$	0.005	0.076	0.245	0.496	0.615
$10^{+1/2}$	NA[a]	0.017	0.175	0.453	0.720
10^{+1}	NA	NA	0.008	0.433	0.594

B. $\nu/\mu=1$ **.**

θ_μ	$\sigma=0$ (neutral)	$\sigma=-10$	$\sigma=-10^{3/2}$	$\sigma=-10^2$	$\sigma=-10^{5/2}$
10^{-1}	0.057	0.063	NA	NA	NA
$10^{-1/2}$	0.034	0.158	0.172	0.625	NA
$1(=10^0)$	0.024	0.086	0.301	0.459	0.739
$10^{+1/2}$	0.008	0.049	0.224	0.427	0.656
10^{+1}	0.000	0.001	0.075	0.463	0.630

C. $\nu/\mu=3$ **.**

θ_μ	$\sigma=0$ (neutral)	$\sigma=-10$	$\sigma=-10^{3/2}$	$\sigma=-10^2$	$\sigma=-10^{5/2}$
10^{-1}	0.046	0.000	NA	NA	NA
$10^{-1/2}$	0.030	0.109	0.333	0.500	NA
$1(=10^0)$	0.029	0.107	0.197	0.444	0.815
$10^{+1/2}$	0.041	0.103	0.238	0.477	0.690
10^{+1}	0.067	0.124	0.247	0.504	0.643

Supplementary table S8. False positive and true positive rates via $Tot^D|_M$, when ν/μ *is known* in advance

NOTE. Here, $n = 100$ and $\alpha = 1$, as well as 5% nominal significance level, are fixed. The tables show proportions of loci that tested positive via $Tot^D|_M$ out of those whose gene genealogies revealed recurrent mutations. ν/μ is the backward/forward ratio of mutation rates. θ_μ ($\equiv 4N\mu$) is the rescaled forward mutation rate. σ ($\equiv 4Ns$) denotes the rescaled selection coefficient.

[a] "NA" is assigned to a category with less than 10 loci with revealed recurrent mutations.

A. $\nu/\mu = 0$ **.**

θ_μ	$\sigma = 0$ (neutral)	$\sigma = -10$	$\sigma = -10^{3/2}$	$\sigma = -10^2$	$\sigma = -10^{5/2}$
10^{-1}	0.055	0.063	0.182	NA	NA
$10^{-1/2}$	0.059	0.053	0.167	0.333	NA
$1(=10^0)$	0.006	0.090	0.274	0.509	0.615
$10^{+1/2}$	NA[a]	0.013	0.179	0.448	0.727
10^{+1}	NA	NA	0.000	0.209	0.568

B. $\nu/\mu = 1$ **.**

θ_μ	$\sigma = 0$ (neutral)	$\sigma = -10$	$\sigma = -10^{3/2}$	$\sigma = -10^2$	$\sigma = -10^{5/2}$
10^{-1}	0.057	0.063	NA	NA	NA
$10^{-1/2}$	0.046	0.208	0.203	0.625	NA
$1(=10^0)$	0.034	0.133	0.371	0.533	0.739
$10^{+1/2}$	0.019	0.110	0.366	0.528	0.706
10^{+1}	0.006	0.018	0.207	0.641	0.749

C. $\nu/\mu = 3$ **.**

θ_μ	$\sigma = 0$ (neutral)	$\sigma = -10$	$\sigma = -10^{3/2}$	$\sigma = -10^2$	$\sigma = -10^{5/2}$
10^{-1}	0.051	0.000	NA	NA	NA
$10^{-1/2}$	0.036	0.118	0.351	0.500	NA
$1(=10^0)$	0.035	0.130	0.236	0.519	0.815
$10^{+1/2}$	0.032	0.095	0.251	0.531	0.746
10^{+1}	0.036	0.068	0.176	0.507	0.730

Supplementary table S9. False positive and true positive rates *via* $Max^D|_M$, when ν/μ is *not* known in advance

NOTE. Here, $n = 100$ and $\alpha = 1$, as well as 5% nominal significance level, are fixed. The tables show proportions of loci that tested positive *via* $Max^D|_M$ out of those whose gene genealogies revealed recurrent mutations. ν/μ is the backward/forward ratio of mutation rates. θ_μ ($\equiv 4N\mu$) is the rescaled forward mutation rate. σ ($\equiv 4Ns$) denotes the rescaled selection coefficient.

[a] "NA" is assigned to a category with less than 10 loci with revealed recurrent mutations.

A. $\nu/\mu = 0$ **.**

θ_μ	$\sigma = 0$ (neutral)	$\sigma = -10$	$\sigma = -10^{3/2}$	$\sigma = -10^2$	$\sigma = -10^{5/2}$
10^{-1}	0.055	0.063	0.182	NA	NA
$10^{-1/2}$	0.055	0.060	0.162	0.333	NA
$1(=10^0)$	0.005	0.076	0.269	0.504	0.615
$10^{+1/2}$	NA[a]	0.021	0.164	0.477	0.726
10^{+1}	NA	NA	0.004	0.411	0.618

B. $\nu/\mu = 1$ **.**

θ_μ	$\sigma = 0$ (neutral)	$\sigma = -10$	$\sigma = -10^{3/2}$	$\sigma = -10^2$	$\sigma = -10^{5/2}$
10^{-1}	0.057	0.063	NA	NA	NA
$10^{-1/2}$	0.035	0.142	0.188	0.625	NA
$1(=10^0)$	0.025	0.099	0.319	0.467	0.739
$10^{+1/2}$	0.006	0.056	0.227	0.443	0.656
10^{+1}	0.000	0.001	0.051	0.440	0.646

C. $\nu/\mu = 3$ **.**

θ_μ	$\sigma = 0$ (neutral)	$\sigma = -10$	$\sigma = -10^{3/2}$	$\sigma = -10^2$	$\sigma = -10^{5/2}$
10^{-1}	0.047	0.000	NA	NA	NA
$10^{-1/2}$	0.033	0.109	0.333	0.500	NA
$1(=10^0)$	0.032	0.118	0.204	0.454	0.815
$10^{+1/2}$	0.040	0.111	0.243	0.492	0.701
10^{+1}	0.052	0.096	0.218	0.489	0.666

Supplementary table S10. False positive and true positive rates via $Tot^D|_M$, when ν/μ is *not* known in advance

NOTE. Here, $n = 50$ and $\alpha = 1$, as well as 5% nominal significance level, are fixed. The tables show proportions of loci that tested positive via $Tot^D|_M$ out of those whose gene genealogies revealed recurrent mutations. ν/μ is the backward/forward ratio of mutation rates. θ_μ ($\equiv 4N\mu$) is the rescaled forward mutation rate. σ ($\equiv 4Ns$) denotes the rescaled selection coefficient.

[a] "NA" is assigned to a category with less than 10 loci with revealed recurrent mutations.

A. $\nu/\mu = 0$ **.**

θ_μ	$\sigma = 0$ (neutral)	$\sigma = -10$	$\sigma = -10^{3/2}$	$\sigma = -10^2$	$\sigma = -10^{5/2}$
10^{-1}	0.000	0.000	NA	NA	NA
$10^{-1/2}$	0.009	0.012	0.037	NA	NA
$1(=10^0)$	0.005	0.026	0.034	0.048	NA
$10^{+1/2}$	NA[a]	0.018	0.091	0.100	0.078
10^{+1}	NA	NA	0.015	0.236	0.199

B. $\nu/\mu = 1$ **.**

θ_μ	$\sigma = 0$ (neutral)	$\sigma = -10$	$\sigma = -10^{3/2}$	$\sigma = -10^2$	$\sigma = -10^{5/2}$
10^{-1}	0.004	0.000	NA	NA	NA
$10^{-1/2}$	0.003	0.000	0.028	NA	NA
$1(=10^0)$	0.005	0.021	0.074	0.022	NA
$10^{+1/2}$	0.007	0.030	0.125	0.126	0.067
10^{+1}	0.001	0.004	0.090	0.254	0.202

C. $\nu/\mu = 3$ **.**

θ_μ	$\sigma = 0$ (neutral)	$\sigma = -10$	$\sigma = -10^{3/2}$	$\sigma = -10^2$	$\sigma = -10^{5/2}$
10^{-1}	0.003	NA	NA	NA	NA
$10^{-1/2}$	0.004	0.012	0.000	NA	NA
$1(=10^0)$	0.008	0.039	0.038	0.000	NA
$10^{+1/2}$	0.037	0.062	0.112	0.097	0.048
10^{+1}	0.064	0.097	0.180	0.271	0.181

Supplementary table S11. False positive and true positive rates via $Tot^D|_M$, when ν/μ is *not* known in advance

NOTE (shared with B and C). Here, $n = 200$ and $\alpha = 1$, as well as 5% nominal significance level, are fixed. The tables show proportions of loci that tested positive via $Tot^D|_M$ out of those whose gene genealogies revealed recurrent mutations. θ_μ ($\equiv 4N\mu$) is the rescaled forward mutation rate. σ ($\equiv 4Ns$) denotes the rescaled selection coefficient.

[a] "NA" is assigned to a category with less than 10 loci with revealed recurrent mutations.

A. $\nu/\mu = 0$.

θ_μ	$\sigma = 0$ (neutral)	$\sigma = -10$	$\sigma = -10^{3/2}$	$\sigma = -10^2$	$\sigma = -10^{5/2}$
10^{-1}	0.046	0.208	NA	NA	NA
$10^{-1/2}$	0.051	0.098	0.242	0.465	NA
$1(=10^0)$	0.020	0.080	0.208	0.349	0.620
$10^{+1/2}$	NA[a]	0.024	0.197	0.498	0.640
10^{+1}	NA	NA	0.010	0.501	0.736

B. $\nu/\mu = 1$.

θ_μ	$\sigma = 0$ (neutral)	$\sigma = -10$	$\sigma = -10^{3/2}$	$\sigma = -10^2$	$\sigma = -10^{5/2}$
10^{-1}	0.069	0.032	0.182	NA	NA
$10^{-1/2}$	0.033	0.109	0.165	0.306	NA
$1(=10^0)$	0.027	0.096	0.191	0.415	0.638
$10^{+1/2}$	0.008	0.064	0.269	0.497	0.639
10^{+1}	0.000	0.001	0.077	0.593	0.712

C. $\nu/\mu = 3$.

θ_μ	$\sigma = 0$ (neutral)	$\sigma = -10$	$\sigma = -10^{3/2}$	$\sigma = -10^2$	$\sigma = -10^{5/2}$
10^{-1}	0.040	0.061	0.263	0.300	NA
$10^{-1/2}$	0.031	0.087	0.228	0.294	0.5000
$1(=10^0)$	0.032	0.108	0.212	0.379	0.548
$10^{+1/2}$	0.050	0.169	0.325	0.507	0.610
10^{+1}	0.063	0.127	0.329	0.632	0.731

Supplementary table S12. Relative frequencies of recurrent mutations captured by gene genealogy, out of polymorphic loci, under expanding population

NOTE. Here, the sample size $n = 100$ and the backward/forward ratio $\nu / \mu = 1$ are fixed. And we used three values of the recombination rate r (per generation). θ_μ ($\equiv 4N\mu$) is the rescaled forward mutation rate. σ ($\equiv 4Ns$) denotes the rescaled selection coefficient.

[a] "NA" is assigned to a category with less than 30 polymorphic loci.

A. $r = 2.6 \times 10^{-3}$.

θ_μ	$\sigma = 0$ (neutral)	$\sigma = -10^{3/2}$	$\sigma = -10^{2}$	$\sigma = -10^{5/2}$
10^{-1}	0.023	0.024	0.021	0.005
$10^{-1/2}$	0.086	0.098	0.087	0.022
$1(=10^{0})$	0.335	0.259	0.197	0.089
$10^{+1/2}$	0.743	0.680	0.518	0.262
10^{+1}	0.993	0.975	0.949	0.673

B. $r = 4.0 \times 10^{-3}$.

θ_μ	$\sigma = 0$ (neutral)	$\sigma = -10^{3/2}$	$\sigma = -10^{2}$	$\sigma = -10^{5/2}$
10^{-1}	0.031	0.042	0.016	0.010
$10^{-1/2}$	0.124	0.120	0.053	0.029
$1(=10^{0})$	0.374	0.360	0.215	0.135
$10^{+1/2}$	0.861	0.786	0.590	0.311
10^{+1}	0.994	0.992	0.974	0.713

C. $r = 5.7 \times 10^{-3}$.

θ_μ	$\sigma = 0$ (neutral)	$\sigma = -10^{3/2}$	$\sigma = -10^{2}$	$\sigma = -10^{5/2}$
10^{-1}	0.057	0.044	0.028	0.004
$10^{-1/2}$	0.161	0.150	0.073	0.033
$1(=10^{0})$	0.492	0.367	0.290	0.095
$10^{+1/2}$	0.892	0.848	0.641	0.299
10^{+1}	0.998	0.991	0.980	0.710

Supplementary table S13. False positive and true positive rates via $Tot^D|_M$, when ν/μ is not known in advance, under expanding population (with *incorrect* r)

NOTE. Here, $n = 100$, $\alpha = 1$, and the backward/forward ratio $\nu/\mu = 1$ are fixed. The nominal significance level is set slightly above the relative frequency of $Tot^D = 2$ conditional on $M = 2$. The tables show proportions of loci that tested positive via $Tot^D|_M$ out of those whose gene genealogies revealed recurrent mutations. θ_μ ($\equiv 4N\mu$) is the rescaled forward mutation rate. σ ($\equiv 4Ns$) denotes the rescaled selection coefficient. Here the null distributions are based on the *incorrect* recombination rate $r = 4.0 \times 10^{-3}$ (per generation).

[a] "NA" is assigned to a category with less than 10 loci with revealed recurrent mutations.

A. $r = 2.6 \times 10^{-3}$

θ_μ	$\sigma = 0$ (neutral)	$\sigma = -10^{3/2}$	$\sigma = -10^2$	$\sigma = -10^{5/2}$
10^{-1}	0.185	0.125	NA [a]	NA
$10^{-1/2}$	0.190	0.329	0.500	NA
$1(=10^0)$	0.103	0.190	0.385	0.650
$10^{+1/2}$	0.083	0.188	0.394	0.650
10^{+1}	0.029	0.095	0.377	0.641

C. $r = 5.7 \times 10^{-3}$

θ_μ	$\sigma = 0$ (neutral)	$\sigma = -10^{3/2}$	$\sigma = -10^2$	$\sigma = -10^{5/2}$
10^{-1}	0.372	0.583	0.833	NA [a]
$10^{-1/2}$	0.288	0.376	0.680	NA
$1(=10^0)$	0.234	0.423	0.655	0.957
$10^{+1/2}$	0.076	0.247	0.520	0.780
10^{+1}	0.016	0.098	0.386	0.607

Supplementary table S14. False positive and true positive rates via Tajima's D test, when ν/μ is not known

NOTE. Here, $n = 100$ and α = 1, as well as 5% nominal significance level, are fixed. The tables show proportions of loci that tested positive *via Tajima's D test,* out of those whose gene genealogies revealed recurrent mutations. ν/μ is the backward/forward ratio of mutation rates. θ_μ ($\equiv 4N\mu$) is the rescaled forward mutation rate. σ ($\equiv 4Ns$) denotes the rescaled selection coefficient.

[a] "NA" is assigned to a category with less than 10 loci with revealed recurrent mutations.

A. $\nu/\mu = 0$ **.**

θ_μ	$\sigma = 0$ (neutral)	$\sigma = -10$	$\sigma = -10^{3/2}$	$\sigma = -10^2$	$\sigma = -10^{5/2}$
10^{-1}	0.042	0.000	0.091	NA	NA
$10^{-1/2}$	0.034	0.043	0.059	0.000	NA
$1(=10^0)$	0.036	0.056	0.044	0.033	0.000
$10^{+1/2}$	NA[a]	0.085	0.056	0.045	0.027
10^{+1}	NA	NA	0.115	0.056	0.045

B. $\nu/\mu = 1$ **.**

θ_μ	$\sigma = 0$ (neutral)	$\sigma = -10$	$\sigma = -10^{3/2}$	$\sigma = -10^2$	$\sigma = -10^{5/2}$
10^{-1}	0.049	0.000	NA	NA	NA
$10^{-1/2}$	0.032	0.075	0.016	0.125	NA
$1(=10^0)$	0.043	0.049	0.044	0.082	0.044
$10^{+1/2}$	0.051	0.063	0.055	0.056	0.033
10^{+1}	0.048	0.052	0.078	0.053	0.046

C. $\nu/\mu = 3$ **.**

θ_μ	$\sigma = 0$ (neutral)	$\sigma = -10$	$\sigma = -10^{3/2}$	$\sigma = -10^2$	$\sigma = -10^{5/2}$
10^{-1}	0.035	0.095	NA	NA	NA
$10^{-1/2}$	0.041	0.025	0.018	0.000	NA
$1(=10^0)$	0.049	0.038	0.052	0.009	0.074
$10^{+1/2}$	0.046	0.043	0.061	0.048	0.061
10^{+1}	0.047	0.042	0.044	0.056	0.043

Supplementary table S15. False positive and true positive rates via the Ewens-Watterson test, when ν/μ is not known

NOTE. Here, $n = 100$ and $\alpha = 1$, as well as 5% nominal significance level, are fixed. The tables show proportions of loci that tested positive *via the EW test,* out of those whose gene genealogies revealed recurrent mutations. ν/μ is the backward/forward ratio of mutation rates. θ_μ ($\equiv 4N\mu$) is the rescaled forward mutation rate. σ ($\equiv 4Ns$) denotes the rescaled selection coefficient.

[a] "NA" is assigned to a category with less than 10 loci with revealed recurrent mutations.

A. $\nu/\mu = 0$ **.**

θ_μ	$\sigma = 0$ (neutral)	$\sigma = -10$	$\sigma = -10^{3/2}$	$\sigma = -10^2$	$\sigma = -10^{5/2}$
10^{-1}	0.036	0.031	0.091	NA	NA
$10^{-1/2}$	0.059	0.035	0.029	0.000	NA
$1(=10^0)$	0.031	0.033	0.044	0.033	0.000
$10^{+1/2}$	NA[a]	0.026	0.046	0.045	0.038
10^{+1}	NA	NA	0.043	0.047	0.031

B. $\nu/\mu = 1$ **.**

θ_μ	$\sigma = 0$ (neutral)	$\sigma = -10$	$\sigma = -10^{3/2}$	$\sigma = -10^2$	$\sigma = -10^{5/2}$
10^{-1}	0.042	0.000	NA	NA	NA
$10^{-1/2}$	0.052	0.025	0.016	0.000	NA
$1(=10^0)$	0.045	0.023	0.023	0.052	0.000
$10^{+1/2}$	0.054	0.054	0.037	0.041	0.017
10^{+1}	0.050	0.061	0.039	0.052	0.030

C. $\nu/\mu = 3$ **.**

θ_μ	$\sigma = 0$ (neutral)	$\sigma = -10$	$\sigma = -10^{3/2}$	$\sigma = -10^2$	$\sigma = -10^{5/2}$
10^{-1}	0.045	0.000	NA	NA	NA
$10^{-1/2}$	0.047	0.034	0.035	0.000	NA
$1(=10^0)$	0.041	0.049	0.049	0.019	0.000
$10^{+1/2}$	0.042	0.047	0.045	0.041	0.056
10^{+1}	0.051	0.042	0.046	0.049	0.033

Supplementary Table S16. False positive and true positive rates *via the Ewens-Watterson test with the lower-tailed P-value*, when ν/μ is *not* known

NOTE. Here, $n = 100$ and $\alpha = 1$, as well as 5% nominal significance level, are fixed. The tables show proportions of loci that tested positive *via the EW test (lower-tailed)* out of those whose gene genealogies revealed recurrent mutations. ν/μ is the backward/forward ratio of mutation rates. θ_μ ($\equiv 4N\mu$) is the rescaled forward mutation rate. σ ($\equiv 4Ns$) denotes the rescaled selection coefficient.

[a] "NA" is assigned to a category with less than 10 loci with revealed recurrent mutations.

A. $\nu/\mu = 0$ **.**

θ_μ	$\sigma = 0$ (neutral)	$\sigma = -10$	$\sigma = -10^{3/2}$	$\sigma = -10^2$	$\sigma = -10^{5/2}$
10^{-1}	0.036	0.031	0.091	NA	NA
$10^{-1/2}$	0.055	0.069	0.029	0.167	NA
$1(=10^0)$	0.061	0.068	0.063	0.041	0.154
$10^{+1/2}$	NA[a]	0.100	0.065	0.067	0.059
10^{+1}	NA	NA	0.097	0.043	0.059

B. $\nu/\mu = 1$ **.**

θ_μ	$\sigma = 0$ (neutral)	$\sigma = -10$	$\sigma = -10^{3/2}$	$\sigma = -10^2$	$\sigma = -10^{5/2}$
10^{-1}	0.031	0.063	NA	NA	NA
$10^{-1/2}$	0.041	0.075	0.031	0.000	NA
$1(=10^0)$	0.047	0.058	0.050	0.089	0.087
$10^{+1/2}$	0.054	0.062	0.065	0.049	0.061
10^{+1}	0.060	0.047	0.065	0.054	0.070

C. $\nu/\mu = 3$ **.**

θ_μ	$\sigma = 0$ (neutral)	$\sigma = -10$	$\sigma = -10^{3/2}$	$\sigma = -10^2$	$\sigma = -10^{5/2}$
10^{-1}	0.048	0.048	NA	NA	NA
$10^{-1/2}$	0.047	0.042	0.070	0.071	NA
$1(=10^0)$	0.038	0.074	0.045	0.046	0.037
$10^{+1/2}$	0.052	0.053	0.044	0.069	0.066
10^{+1}	0.055	0.040	0.059	0.056	0.047

Supplementary figure S1. Possible causes of overestimated numbers of mutation events and our counter-measures.

(A) Usually, a set of sequences collected from a population does not have enough SNPs to fully resolve the genealogical relationships among them, leaving some interior branches unresolved (blue and green thick lines labeled "X" and "Y", respectively, with their descendant samples colored in the same way). Each yellow lightening bolt denotes a point mutation generating a SNP site. (**B**) Therefore, usually, SNPs *alone* should only enable us to reconstruct a multi-furcated tree at best. (The panel shows only the part of the genealogy in panel A enclosed by the red dotted frame.) **(C)** Nevertheless, most existing algorithms forcefully reconstruct a "fully-resolved" tree by arbitrarily inserting interior branches (red lines labeled as "Z" and "W"). Each of such arbitrary branches could split a cluster of identical-by-descent mutant states. Thus, when mutations (at non-SNP sites/loci) occur along branches not supported by SNPs (blue and green lightening bolts in panel **D**), the arbitrary branch could cause an overestimated number of mutation events (red lightening bolts in panel **E**). **(F)** To prevent such over-counting, we therefore removed, from the genealogy reconstructed by an existing algorithm, interior branches not supported by any SNPs. (**G)** Even in such an "SNP-supported" multi-furcated genealogy, however, existing parsimony algorithms still over-count mutation events along unresolved branches. (**H)** To avoid such over-counting, we developed a new parsimony algorithm. The algorithm, if necessary, puts nodes with the same state (and immediately under the same multi-furcated node) under an additional branch (like blue and green thick branches labeled as "X" and "Y"), partially resolving genealogical relations. Then, the algorithm assigns parsimonious mutation events (blue and green lightening bolts) to the added branches. Actually, the algorithm enumerates all the possible parsimonious scenarios with all the possible combinations of such additional branches and nodes (see *Additional File 2* for details on the algorithm itself).

(Supplementary figure S1)

A. Original sequence genealogy.

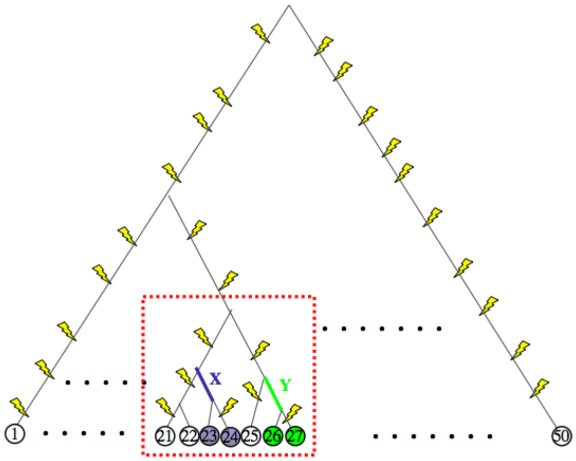

B. Genealogy maximally resolved by SNPs *alone*.

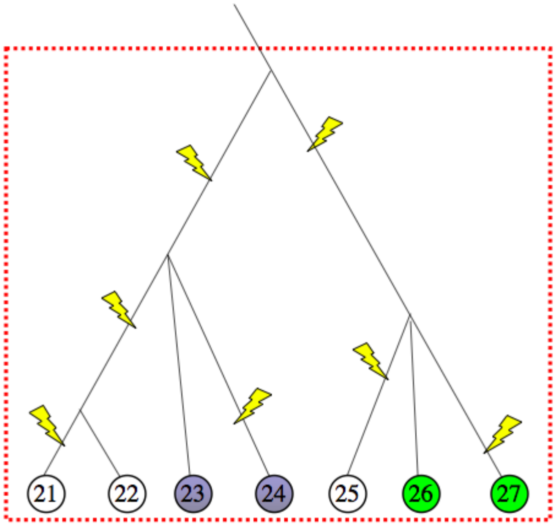

C. Genealogy arbitrarily resolved by an existing tree-reconstruction algorithm.

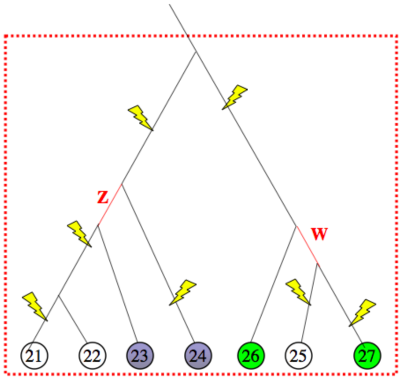

D. Correct mutation scenario (example).

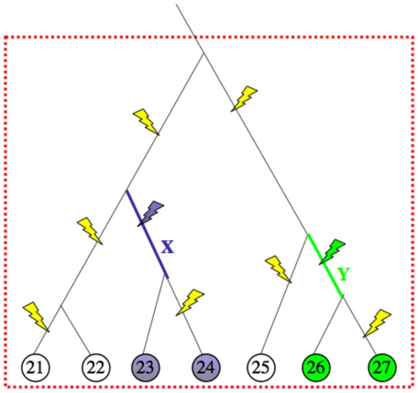

E. Over-counting due to arbitrary branches.

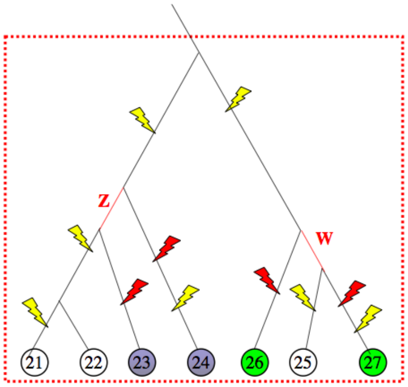

F. First step: removing interior branches not supported by SNPs.

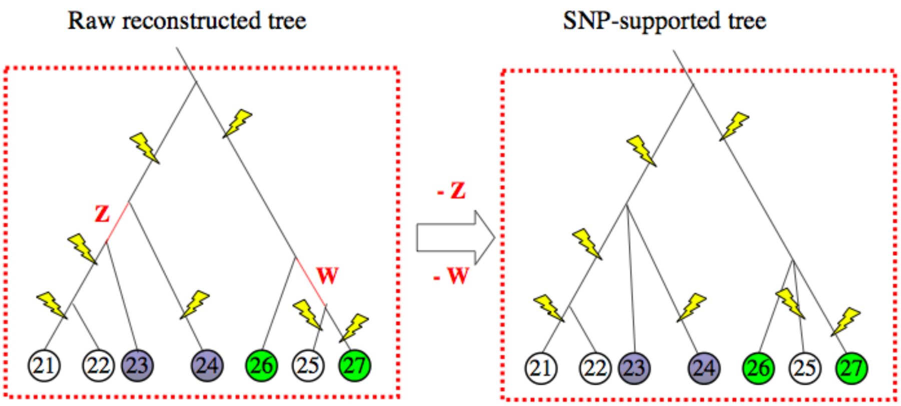

G. Over-counting due to unresolved genealogical relationships.

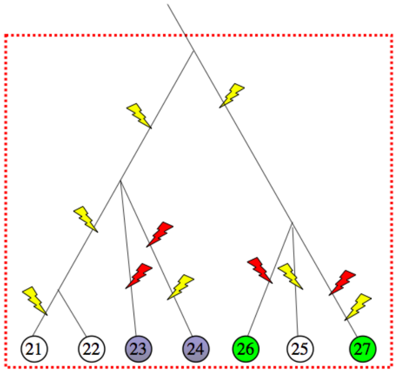

H. Second step: merging nodes sharing the same mutation state under the same multi-furcated node, using our new parsimony algorithm.

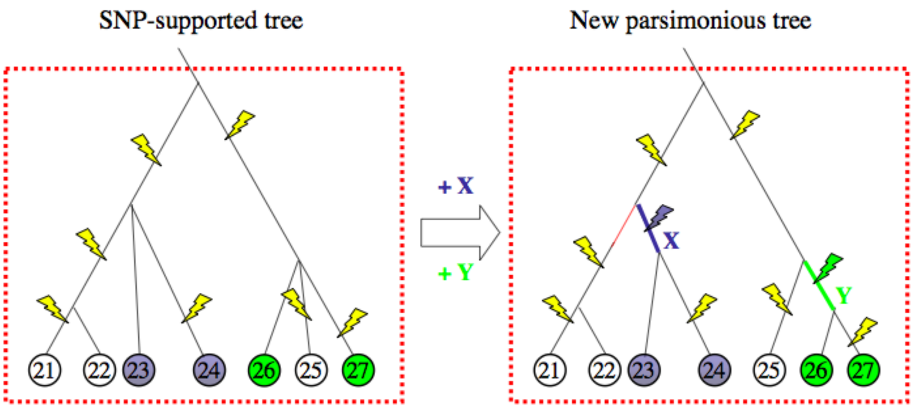

Supplementary figure S2. Composition of the number of mutations at each recurrently mutating locus.

This figure is for a fixed backward/forward ratio (ν/μ = 0) and a fixed sample size (n = 100). The parameter setting, panel assignment, labeling and color code of the compositions are basically the same as those for Figure 5, except that the backward/forward ratio is fixed as ν/μ = 0 here. Note that the compositions for $\theta\mu$ = 3.16 and 10 are missing in panel A (with $\sigma = 0$), because of insufficient numbers of sampled loci with such parameter combinations.

A. $\sigma = 0$ **(Selectively neutral)**

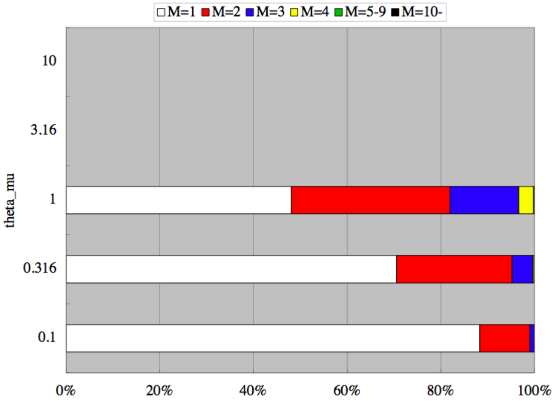

B. $\sigma = -10^{+3/2}$ **(Moderately deleterious)**

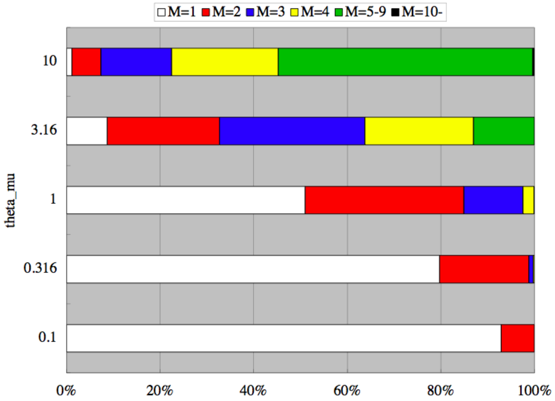

C. $\sigma = -10^{+5/2}$ **(Strongly deleterious)**

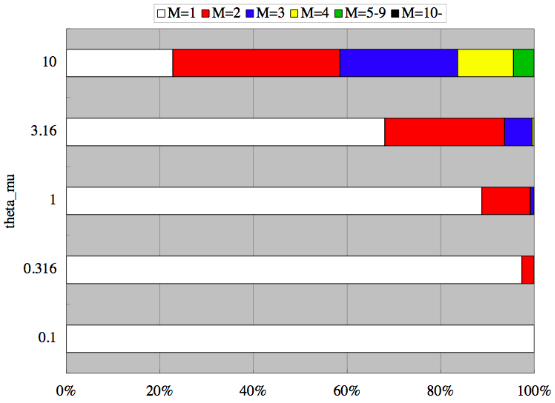

Key:

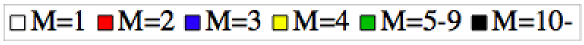

Supplementary figure S3. Composition of the number of mutations at each recurrently mutating locus.

This figure is for a fixed backward/forward ratio (ν/μ = 3) and a fixed sample size (n = 100). The parameter setting, panel assignment, labeling and color code of the compositions are basically the same as those for Figure 5, except that the backward/forward ratio is fixed as ν/μ = 3 here.

A. $\sigma = 0$ **(Selectively neutral)**

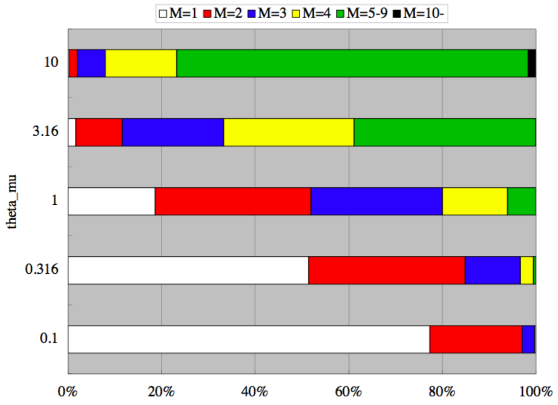

B. $\sigma = -10^{+3/2}$ **(Moderately deleterious)**

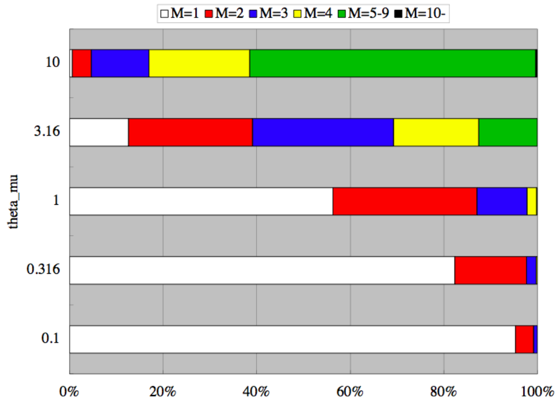

C. $\sigma = -10^{+5/2}$ **(Strongly deleterious)**

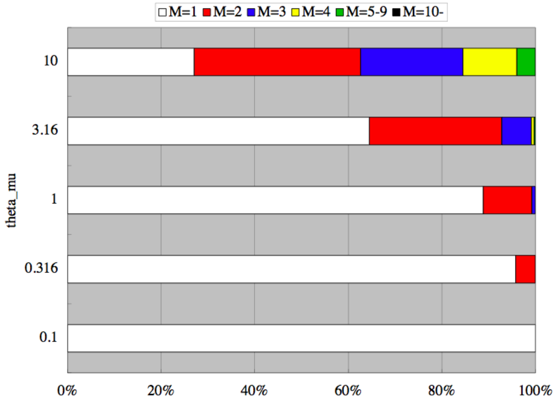

Key:

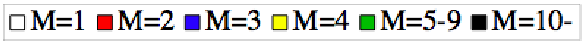

Supplementary figure S4. Cumulative distributions of our new test statistic, $Tot^D|_M$, under selective neutrality ($\sigma = 0$).

The panel assignment, specifications, etc. are basically the same as those for Figure 6.

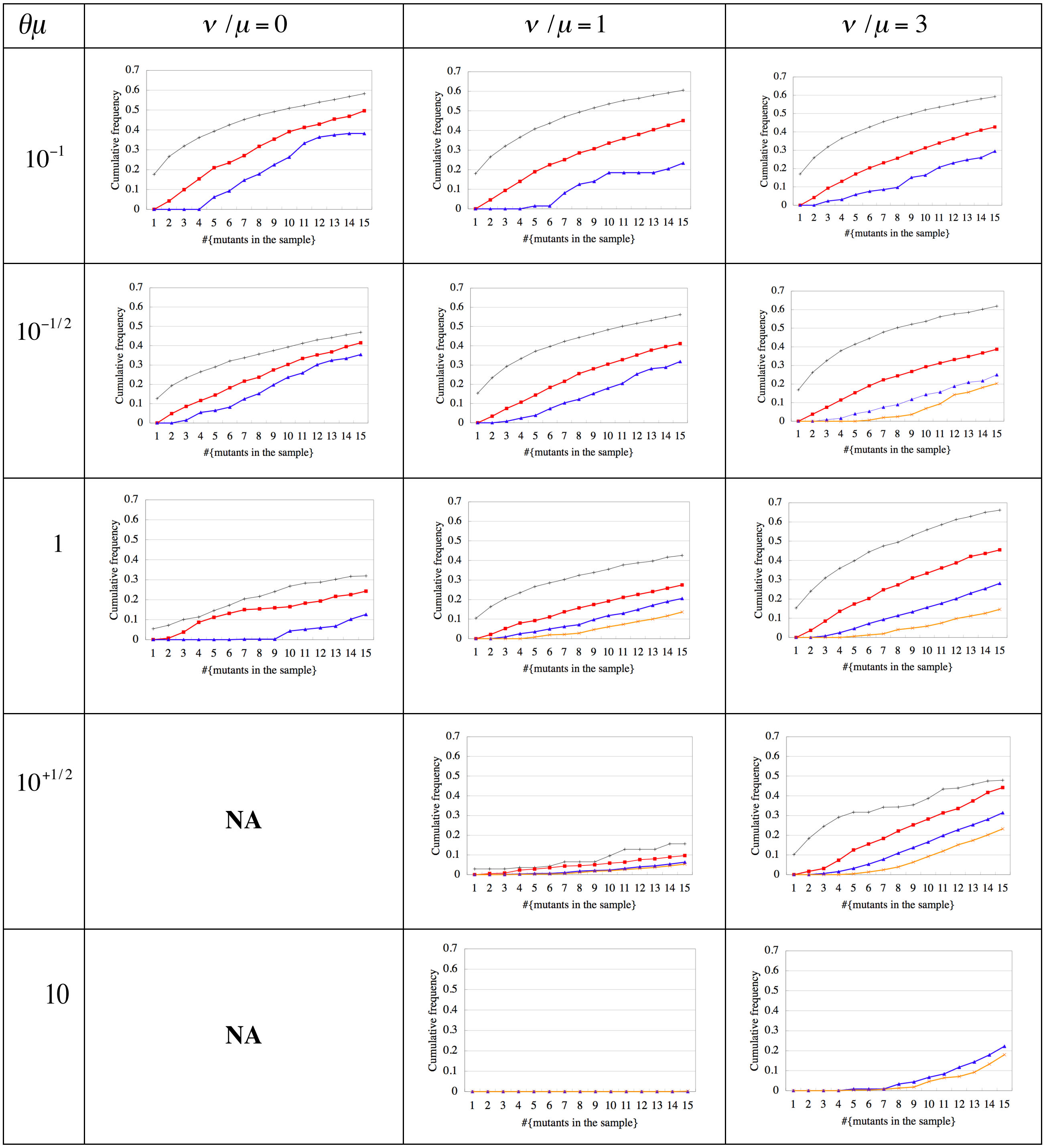

Key:

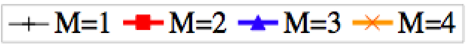

Supplementary figure S5. Cumulative distributions of our new test statistic, $Max^D|_M$, under strong negative selection ($\sigma = -100$).

The panel assignment, specifications, etc. are basically the same as those for Figure 6.

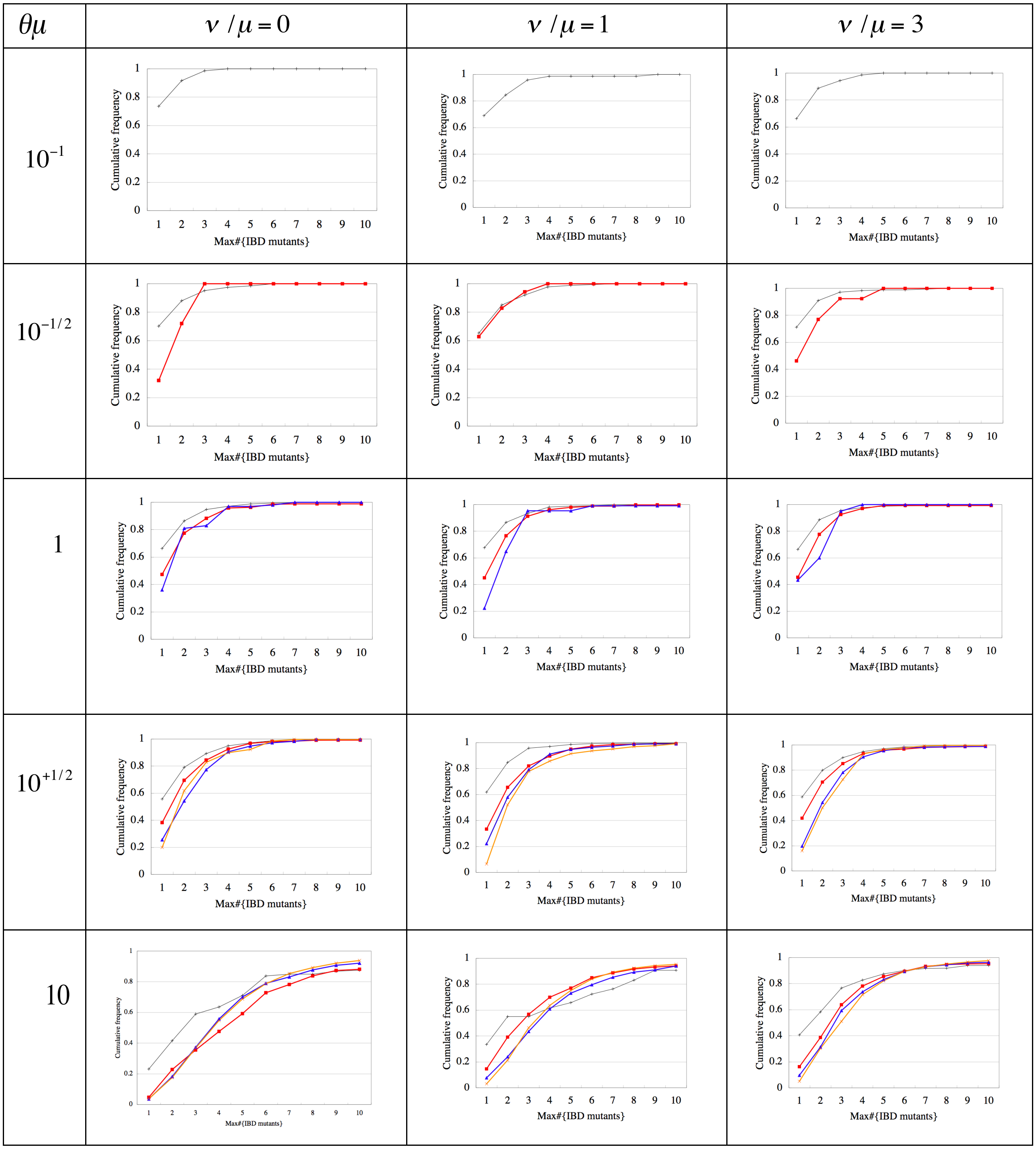

Key:

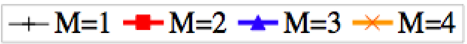

Supplement: Additional file 1 — Supplementary notes, which consist of Supplementary methods, Supplementary discussion, Tables S1- S16, and Figures S1-S5. [file 1471-2156-14-37-S1.pdf]
