# Supplementary material for: Detecting negative selection on recurrent mutations using gene genealogy
Source: BMC Genet. 2013 May 7;14:37. doi: 10.1186/1471-2156-14-37 (PMC3661350; doi:10.1186/1471-2156-14-37)

ADDITIONAL FILE 2 for

"Detecting negative selection on recurrent mutations using gene genealogy"

-- New Algorithm to Enumerate Parsimonious Mutation Scenarios Consistent with an Incompletely Resolved Genealogy --

by Kiyoshi Ezawa, Giddy Landan, and Dan Graur

Table of Contents:

Supplementary^2 methods --- **pp.2-11.**

References cited --- **p.11.**

Supplementary figures SS1-SS13 --- **pp.12-28.**

Supplementary^2 methods

Rationale behind and overview of the new parsimony algorithm

As explained in the main *Methods*, our new test statistics, $Max^D|_M$ and $Tot^D|_M$, cannot be calculated until we know the number M of forward mutations across the genealogy, along with the numbers, $(D_1, D_2, ..., D_M)$, of identical-by-descent mutants under the forward mutation events. One simple method to estimate these numbers would be to apply a maximum parsimony algorithm to the sampled sequences and their genealogy. A problem, however, on the genealogy in population genetics is that it is usually incompletely resolved because the focal locus is linked to only a limited number of polymorphic sites (e.g. Supplementary figure S1 B or F in Supplementary notes, i.e. Additional file 1). Thus, if a mutation at the focal locus occurred along an unresolved branch, traditional parsimony algorithms (e.g. [1, 2]) will overestimate the number of mutations (Supplementary figure S1). Or, even worse, they could erroneously infer the state at the incompletely resolved (i.e. multi-furcated) node (Supplementary figure SS1 B). Given that a majority of SV sites have a modest mutation rate ($\theta\mu < 1$), this will cause a serious over-counting problem in genome-wide analyses.

Therefore, we devised an algorithm that errs on a conservative side. The algorithm clusters together mutants of the same state sharing a parent node by placing them under an additional branch (Supplementary figures S1 H and SS1 C). If all the children of a multi-furcated node have their own unique states (Supplementary figure SS2 A), the problem is simple. All you have to do is to put together the child nodes sharing the same state, and insert a new node per each state (except the state of the originally multi-furcated node) (Supplementary figure SS2 B). A complexity arises when some of the child nodes have two or more possible states each (Supplementary figure SS2 C). In such cases, the child nodes could be clustered differently depending on the choice of the alternative states (Supplementary figure SS2 D). Our algorithm is designed to correctly handle such cases as well.

And last but not least, our algorithm enumerates all possible parsimonious scenarios consistent with the input genealogy, each accompanied by a set of additional branches that are necessary. In a sense, the algorithm is a modified extension of Sankoff's (1975) parsimony algorithm. Thus, theoretically, it could be applicable to any penalty matrix, $pen(s,s')$, not necessarily limited to the Jukes-Cantor (JC)-type penalty matrix [3]: $pen(s,s)=0$ and $pen(s,s')=1$ for $s \neq s'$ (s and s' denote character states), although the current implementation of the algorithm can correctly handle only JC-type penalties. Extension to more general penalty

matrices (or schemes) will be implemented in the future.

In the following subsections, we will describe the algorithm, the overall structure first and details next.

Overall workflow of the new parsimony algorithm

Supplementary figure SS3 shows the schematic images of the broad processes involved in the new parsimony algorithm. The input data consists of the states of the sampled sequences at a focal site/locus and an incompletely resolved genealogy of the sequences (panel A of SS3). Then the algorithm consists of two broad procedures: the first one proceeds from bottom up, and calculates the minimum possible penalties for the sub-tree under each interior node, conditional on each possible state at the node (nearby mini table), while inserting additional nodes (red-rimmed circles) and branches (red segments) if necessary (panel B of SS3); and the second one proceeds from top down, choosing the states (and accompanying additional branches) that give the minimum penalty at each node, finally enumerating all possible mutation scenarios (red lightening bolts) providing the minimum total penalty (panel C of SS3).

(I) Bottom-up part of the new parsimony algorithm

The first large block of procedures starts from the exterior nodes (i.e. tips), and goes up step by step until it reaches the top node (i.e. the root). At each node n, the algorithm calculates the '*conditional*' minimum total penalty, $Pen[n \mid s]$, incurred across the sub-tree under the node, on condition that the node exhibits each state s belonging to the character state space, St. Such conditional minimum penalties can be calculated iteratively, given a matrix of penalties, $pen(s, s')$, from states $s \in St$ to states $s' \in St$, as well as the conditional penalties assigned to the "child nodes", namely the nodes immediately under the subject node (Supplementary figure SS4 A).

When the tree is completely resolved (i.e. bifurcated), the algorithm is nothing other than Sankoff's parsimony algorithm (Supplementary figure SS4 A), which is defined by the following recursion equation:

$$Pen[n \mid s] = \sum_{n' \in Ch(n)} min_{s' \in St} \{pen(s,s') + Pen[n' \mid s']\}, \qquad \text{--- Eq.(SS1)}$$

where $Ch(n)$ is the set of all child nodes of the node n.

If the node under consideration is multi-furcated (Supplementary figure SS4 B),

clustering child nodes having the same state will always reduce the penalty (Supplementary figure SS4 C), as long as there is no state pair, $s, s'(\neq s)$, such that $pen(s, s') = 0$. Let us first consider a simplest situation where every child node $n'(\in Ch(n))$ has its own 'unique character state'. The 'unique character state', $s(n')$, is defined as the character state such that:

$$pen(s'', s(n')) + Pen[n' \mid s(n')] < pen(s'', s') + Pen[n' \mid s'] \quad \text{--- Eq.(SS2)}$$

for $^\forall s' (\neq s(n')) \in St$ and $^\forall s'' \in St$.

Then, the smallest penalty, conditional on a state s on the node n, is obtained by putting all the child nodes with the same unique character state under a new 'mediating node' and by connecting all the new mediating nodes, as well as node n, so that the sum of mutation penalties, $\sum_{e:\, new\ branches} pen(s(u(e)), s(l(e)))$, is minimized (Supplementary figure SS5 A). Here $u(e)$ and $l(e)$ denote the upper-node and the lower-node, respectively, of a new branch e, which will be introduced only when a mutation is necessary. When a JC-type penalty is used, the second step is easy: we just connect the new mediating nodes with node n via a new branch when the unique character state is different from the state s, and connect the child nodes directly with node n when their unique character state is s (Supplementary figure SS5 B). [Although there could be numerous other topologies connecting mediating nodes that provide the same minimum penalty, dwelling on them is unproductive without other information. We employ the 'least resolved' topology providing the minimum penalty because it is the most flexible.] When the penalty matrix is not of JC-type, we will have to find the least penalized topology connecting new mediating nodes under n. An efficient algorithm to achieve this job is now under development. Hereafter, we will thus focus on the parsimonious scenarios under the JC-type penalty.

In general, all child nodes do not necessarily have their own unique character states. In such cases, there may be or may not be a unique parsimonious scenario depending on the situation. Although parsimonious scenarios may be enumerated by exhaustively examining all the combinations of possible states at the child nodes $n'(\in Ch(n))$, it could be prohibitively time-consuming. For example, if there are ten child nodes and four alternative character states for each node, we have to examine $4^{10} \approx 10^6$ combinations. We therefore devised an algorithm that efficiently enumerates the parsimonious scenarios for the node n and its children $(\in Ch(n))$, under the JC-type penalty. The algorithm will be explained in the next section. Because the processes at a bifurcated node are identical to those of Sankoff's parsimony algorithm, we will

mainly describe the processes on a multi-furcated node.

(I-1) Components of the bottom-up part

Supplementary figure SS6 shows the overall workflow of component processes at each node, which make up the recurrent procedure for the bottom-up part of the algorithm.

(o) The input data at each node n are (Supplementary figure SS6, step o; henceforth "Supplementary figure SS6" will be omitted): a set of possible character states, St; a penalty matrix, $[p(s,s')]_{s,s' \in St}$; a set of the child nodes of n, $Ch(n)$; conditional minimum penalty, $Pen[n' \mid s']$, for each child node $n' \in Ch(n)$ and for each character state $s' \in St$.

(i) Classify the child nodes into those with their own "quasi-unique character states" and those without ones (step i). The "quasi-unique character state" $\hat{s}(n')$ of a child node $n'(\in Ch(n))$ is defined as in Eq.(SS2) with the '$<$' replaced by '$\leq$', namely:

$$pen(s'', \hat{s}(n')) + Pen[n' \mid \hat{s}(n')] \leq pen(s'', s') + Pen[n' \mid s'] \qquad \text{--- Eq.(SS3)}$$

for $^\forall s' (\neq \hat{s}(n')) \in St$ and $^\forall s'' \in St$. Then, the quasi-unique character states are tentatively assigned to the former class of child nodes. The character states that are (quasi-)unique to some child node(s) , as well as the current subject state s, are classified as "primary" characters $\{s_P\}$. And the remaining character states are classified as "secondary" characters $\{s_S\}$. The child nodes *without* their own quasi-unique character states will be referred to as "remaining" child nodes.

(ii) Tentatively cluster together the child nodes sharing the same quasi-unique character state, under a newly added node (step ii). This is done for each of the primary characters $\{s_P\}$ other than the subject state s.

(iii) Now the tough part begins, in which we will assign character states to the remaining child nodes, in such a way that the total penalty is the smallest. For this purpose, we first identify the 'best' primary character, $s_{BP}(n'')$, for each such child node n'', which gives the smallest $Pen[n'' \mid s_P]$ among the primary characters $\{s_P\}$. The vector, $\{Pen[n'' \mid s_{BP}(n'')]\}$, which spans the remaining child nodes $\{n''\}$, will serve as a basis for the subsequent processes (step iii).

(iv) Second, compare the penalties, $\{Pen[n'' \mid s_S]\}$, of each secondary character s_S on the remaining child nodes $\{n''\}$, with the penalties of the 'best' primary characters, $\{Pen[n'' \mid s_{BP}(n'')]\}$, component by component. If

$$Min\{\Delta Pen\}[s_S \mid \{s_{BP}(n'')\}] \equiv \underset{s_P:primary}{Min}\{pen(s_P, s_S)\} + \sum_{n'':remaining} Min\{Pen[n'' \mid s_S] - Pen[n'' \mid s_{BP}(n'')],\ \ 0\} \ < 0 ,$$

--- (Eq. SS4)

the secondary character s_S can improve the total penalty. Such characters will be called "(penalty-)improving secondary characters", and will be used for the next procedure (step iv). Secondary characters with $Min\{\Delta Pen\}[s_S \mid \{s_{BP}(n'')\}] = 0$ will be referred to as "(penalty-)keeping secondary characters" and will also be kept for later use, because they might keep the minimum total penalty and provide alternative parsimonious scenarios (step iv).

(v) Then, for each secondary character s_S categorized as "improving" or "keeping" at some node, the remaining child nodes $\{n''\}$ will be sorted out into three classes: "key" nodes satisfying $Pen[n'' \mid s_S] < Pen[n'' \mid s_{BP}(n'')]$, "marginal" nodes satisfying $Pen[n'' \mid s_S] = Pen[n'' \mid s_{BP}(n'')]$, and the rest. The "rest" will be dismissed because they will never improve the penalty (step v).

(vi) Using only the improving secondary characters and placing them solely into some of their key nodes, we will find "core" combinations of the characters that minimize the total penalty (step vi). In consequence, the minimum total conditional penalty, $Pen[n \mid s]$, will also be determined. Details on this process will be described in the next subsection (and illustrated in Supplementary figures SS7 and SS8).

(vii) Based on each core combination of the improving secondary characters, try to make "fleshed-out" alternative combinations by adding other improving secondary characters onto some of their key nodes (step vii and Supplementary figure SS9). The characters will be tried one by one recursively from the least penalized one, and all promising combinations will be exhausted. Basically, each such character s'_S will be examined at its key nodes. At each key node n'', the character will tentatively replace the 'current best' character(s) $\{s_{CB}(n'')\}$ if $Pen[n'' \mid s'_S] < Pen[n'' \mid s_{CB}(n'')]$, and will be tentatively kept as an alternative if $Pen[n'' \mid s'_S] = Pen[n'' \mid s_{CB}(n'')]$. If the total penalty, including the penalty on the additional mutation, $\underset{s_P:primary}{Min}\{pen(s_P, s'_S)\}$, equals the minimum penalty, such addition of s'_S will be employed as an alternative minimum-penalty combination of secondary characters (Supplementary figure SS9).

(viii) Then, from each "fleshed-out" alternative combination, try to make *further* 'fleshed-out' alternatives by adding keeping secondary characters onto some of their key nodes

(step viii), in a manner similar to that for (vii). All promising combinations will be tried.

(ix) Then, from each further alternative combination, try to make *yet further* alternative combinations by putting each of employed improving and keeping secondary characters into their marginal nodes that are *not* occupied by (an)other secondary character(s) yet (step ix). All such combinations will be exhausted.

(x) Then, fill in still vacant remaining nodes, n'', with the best primary characters $\{s_{BP}(n'')\}$, making the "full" combinations of the characters assigned to $\{n''\}$ (step x). All possible combinations of $\{s_{BP}(n'')\}$ will be tried. In the actual implementation, this step and step (ix) were lumped together and performed in a manner that the vacant "remaining nodes" are filled in with $\{s_{BP}(n'')\}$ and with characters used in step (ix) if any, iteratively node by node

(xi) Finally, for each "full" combination, cluster the child nodes that the same character state $s' (\in St)$ is assigned to under an additional node if $s' \neq s$, or just put them under the node n if $s' = s$ (step xi and Supplementary figure SS10 B). The additional nodes bearing primary characters will be placed under the node n. The node occupied by a secondary character s_S will be placed under the node bearing the primary character s_P that has the minimum mutation penalty $pen(s_P, s_S)$, or under the node n if $\underset{s_P:\,primary}{Min}\{pen(s_P, s_S)\} = pen(s, s_S)$ (step xi and Supplementary figure SS10 C). Under a JC-type penalty matrix, this topology will genuinely minimize the total penalty, while leaving the genealogical relationship least resolved.

(xii) As a follow-up, examine clusters each consisting only of a single child node $n' (\in Ch(n))$ bearing its own quasi-unique character $\hat{s}(n')$. For each of such clusters, first we remove the newly introduced "mediating" node, because it is not necessary (step xii and Supplementary figure SS11 B). Then, if
$pen(s, \hat{s}(n')) + Pen[n' \mid \hat{s}(n')] = Pen[n' \mid s']$ for some other character $s' (\neq \hat{s}(n'))$ employed in the current character mapping onto child nodes, we also employ an alternative mapping whose only difference from the original one is the character at node n', which is s' instead of $\hat{s}(n')$, and incorporate the node n' into the cluster whose assigned character is s' (step xii and Supplementary figure SS11 C). For each character mapping (and the resulting minimally resolved genealogy), we perform this process iteratively on each single child node bearing its own quasi-unique character, to exhaust all possible such alternative mappings (and therefore minimally resolved genealogies).

(I-2) Components of the core process (step (vi) in part I)

Here we will detail the core process in the bottom-up part of the algorithm, which searches for the "core" combinations of "improving secondary characters" that minimize the total penalty, and which was briefly described in step (vi) of the last subsection (step vi of Supplementary figure SS6). The workflow of this core process is illustrated in Supplementary figure SS7 (henceforth only step IDs will be referred to).

(o) First, the improving secondary characters, $\{s_S\}_{Min\{\Delta Pen\}[s_S]<0}$, are sorted in ascending order of potential minimum (relative) penalty, $Min\{\Delta Pen\}\left[s_S \mid \{s_{BP}(n'')\}\right]$ (defined in Eq.(SS4)), and will be numbered as $\{s_{S1}, s_{S2}, \ldots, s_{SI}\}$ with $I \equiv$ #{improving secondary characters} (step (o) and Supplementary figure SS8 A). The process starts at the "best" character s_{S1}, and proceeds downwards, looping over the order $i\,(=1,2,\cdots,I)$ of the improving secondary character. The initial candidate of the "core" combination is given by (s_{S1}) (step (o)).

(a) For the i th cycle in the loop, the first process is to compare the potentially achievable minimum total penalty, secondary $\sum_{j=i}^{I} Min\{\Delta Pen\}\left[s_{Sj} \mid \{s_{BP}(n'')\}\right]$, with the current minimum total penalty, $\sum_{S_S \in \{a\ current\ core\ combination\}} Min\{\Delta Pen\}\left[s_S \mid \{s_{BP}(n'')\}\right]$, both defined as in Eq.(SS4). If the former is larger than the latter, the loop is terminated immediately (step (a)).

(b) Second, using s_{Si} as a basis, a "crude combination" is created by trying to add $s_{Si+1}, s_{Si+1}, \cdots, s_{SI}$ in a single downhill run of trials (step (b)). In each trial (concerning the character s_{Sj}), only the "key" nodes of s_{Sj} will be examined (red cells in Supplementary figure SS8 A). At each key node n'', the character s_{Sj} will be tentatively placed if the node has not yet accommodated a secondary character, will tentatively replace the "current best" secondary character(s) $\{s_{CB}(n'')\}$ if $Pen[n'' \mid s_{Sj}] < Pen[n'' \mid s_{CB}(n'')]$, and will be tentatively kept as an alternative if $Pen[n'' \mid s_{Sj}] = Pen[n'' \mid s_{CB}(n'')]$. If the total penalty, including the penalty on the additional mutation, $\underset{s_P:\,primary}{Min}\{pen(s_P, s_{Sj})\}$, is smaller than the current minimum penalty in the i th cycle, such addition of s_{Sj} will replace the current "crude combination" of secondary characters (Supplementary figure SS8 B).

(c) Once gone downhill to the bottom, we will try to further improve the crude combination thus created, $(s_{Si}, s_{Si_1}, \ldots, s_{Si_C})$ (with $i < i_1 < \cdots < i_C \le I$). For this purpose, we will first go one step up by removing s_{Si_C}, and try going downhill again, this time from s_{Si_C+1} on. To

decide whether we should actually try the downhill or not, we monitor the potentially achievable penalty similarly to the process (a). Then, the downhill process will be performed similarly to the process (b) (step (c) and Supplementary figure SS8 C). After the downhill process ends, the best combination in the process replaces the set of previous best combinations if the former is better than the latter, or the former is added to the latter if they tie. The resulting improved combination will be further tried to improve similarly, and the whole process goes on until the basis, s_{Si}, is finally removed.

(d) The best combination in the i th cycle will be compared to the current best combination in the whole process (step (d)). If the former improves the penalty, it replaces the latter. If they tie, the former will be added to the set of current bests.

(II) Top-down part of the new parsimony algorithm

The second large block of procedures starts at the top node (i.e. root), and goes down until it goes through all the nodes. The block is an extension of the top-down part of Sankoff's parsimony algorithm [2], which enumerates all the possible parsimonious mutation scenarios.

When the input tree is completely resolved (i.e. bifurcated), the whole process is exactly that of Sankoff's algorithm [2]:

(i) To the top node (i.e. root) n_{TOP}, assign the character state(s) that minimize(s) the conditional total penalty, $Pen[n_{TOP} \mid s]$ (Supplementary Figure SS12 A);

(ii) To each of other nodes, n, given a state $s^{(P)}$ at its parent node $n^{(P)}$, assign the character state(s), $\{s\}$, that minimize(s) the summed penalty: $pen(s^{(P)}, s) + Pen[n \mid s]$ (Supplementary Figure SS12 B).

When there are more than one minimum-penalty characters at a node, as many parsimonious scenarios as the minimum-penalty characters are created from the parsimonious scenario constructed down to the immediately previous node.

Even when the tree is incompletely resolved (i.e. multi-furcated), the step (i) remains the same. The step (ii), however, appears quite different for a multi-furcated node from that for a bifurcated node (given above), though they are equivalent in philosophy. Thus:

(ii') Given a state at a multi-furcated node, n, a set of parsimonious mutation scenarios down to its children, $\{n'\}$, will be automatically provided as an output of the bottom-up block of the algorithm. Each parsimonious scenario in turn will automatically assign character states to the child nodes (Supplementary figure SS12 C).

Output format

This algorithm enumerates the most parsimonious scenarios, whose mutations incur the minimum penalty in total, and that is consistent with the input incompletely resolved (i.e. multi-furcated) genealogy, as well as the current states, of the sequences. Each scenario accompanies additional branches and nodes necessary to achieve the minimum penalty.

It is not uncommon that there are hundreds to thousands of parsimonious scenarios, which will be hard to be stored in memory in the normal format of states mapped onto the interior nodes, especially given that the tree topology could differ from scenario to scenario. To alleviate the possibly enormous memory consumption, we introduced the following output format:

(1) Each scenario is represented in terms of changes (mutations) mapped onto the branches, instead of (ancestral) states mapped onto the (interior) nodes (Supplementary figure SS13 A). This saves memory especially when only a small number of changes are mapped onto the tree of a large number of sequences.

(2) Lump together scenarios with the same mutations mapped onto the branches immediately under the bifurcated nodes in the input (incompletely resolved) genealogy (Supplementary figure SS13 B). This enables to factorize a group of such scenarios into the mapping of mutations onto the branches under bifurcated nodes and a set of mappings, each of which maps mutations occurring somewhere from a multi-furcated node to its children (Supplementary figure SS13 C).

(3) Instead of storing the set of whole scenarios, store the sets of *components* of scenarios, factorized as in feature (2) (Supplementary figure SS13 C). This could save some memory space.

Possible Future Improvements

One relatively easy improvement would be to move processes (vii) – (xii) of the bottom-up block to the top-down block. Currently, these processes for non-parsimonious states (on the node n) generate data that will not be used in the later steps; all we need is the minimum total penalties, $\{Pen[n \mid s]\}$, conditional on the states $\{s\}$ at the node n, which will be calculated through the processes (i) - (vi). Therefore, this transfer of processes (vii) – (xii) will substantially save computation time and memory.

Also, as mentioned earlier, although the current implementation correctly handles JC-type penalty matrices, this may not be the case with non-JC-type penalties. To be applicable to such penalties, we will have to consider connecting two or more nodes to which secondary characters are assigned. This will complicate the comparison of mutation penalties, $\{pen(s', s'')\}$, and will be tackled in the future.

Another possibly useful modification would be to extend the algorithm so that it can accept a set of inconsistent partitions, instead of a (possibly multi-furcated) genealogy. This will give a firm foundation for handling sequences under recombination.

Program availability

The new parsimony algorithm was implemented in a home-made Perl module, which is called by a home-made Perl script that applies our new neutrality tests to an output file of the coalescent simulator '*msms*' [4] (under a particular set of options).

A package containing the Perl module and the Perl script is available as Additional File 3, or on request to the first author at <kezawa dot ezawa3 at gmal dot com> (replace ' dot ' with '.', and ' at ' with '@').

References cited

1. Fitch WM: **Toward defining the course of evolution: Minimum change for a specified tree topology.** *Syst Zool* 1971, **20**:406-416.
2. Sankoff D: **Minimal mutation trees of sequences.** *SIAM J Appl Math* 1975, **28**:35-42.
3. Jukes TH, Cantor CR: **Evolution of protein molecules.** In *Mammalian protein metabolism*. Volume III. . Edited by Munro MN: ; 196:21-132.
4. Ewing G, Hermisson J: **MSMS: a coalescent simulation program including recombination, demographic structure, and selection at a single locus.** *Bioinformatics* 2010, **26**:2064-2065.

Supplementary figure SS1. Another reason for our new parsimony algorithm.

This figure schematically illustrates common instances where traditional parsimony algorithms (e.g. Sankoff 1975) erroneously infer the mutation scenario, as well as our solution to the problem. **(A)** *The correct* genealogy of an input set of sequences (numbered circles at the bottom), with point mutations at the SNP sites (yellow lightening bolts) mapped on it. A red lightening bolt and red solid circles denote a mutation at the recurrently mutating site/locus and the resulting mutants, respectively. A circle marks an interior node of our focus, with white indicating the wild-type state (at the recurrently mutating site/locus). **(B)** In practice, interior branches *not* supported by SNPs cannot be resolved. In such an incompletely resolved genealogy, traditional parsimony algorithms often infer ancestral states erroneously (red solid circle at the interior node), resulting in an erroneous mutation scenario (red and blue lightening bolts). **(C)** In some of such cases, our new parsimony algorithm can correctly infer the ancestral states and the mutation scenario, by clustering sequences sharing a mutant state under an additional branch (thick line).

A. Correct genealogy

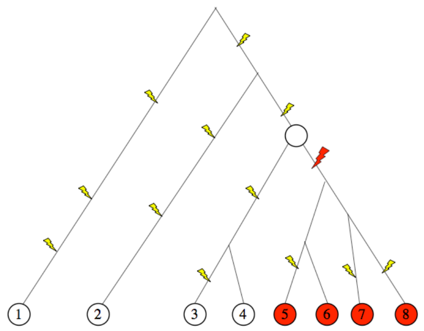

B. Traditional parsimony algorithm

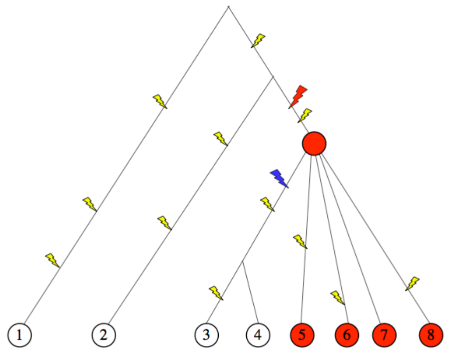

C. Our new parsimony algorithm

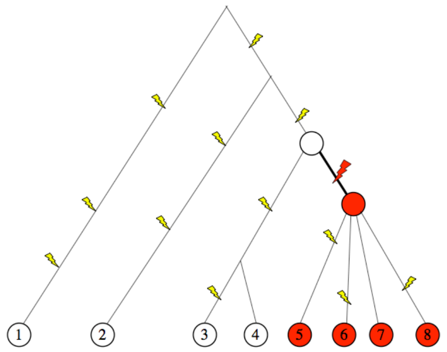

Supplementary figure SS2. Rough illustration of our new parsimony algorithm.

To schematically illustrate what out new parsimony algorithm does, a multi-furcated node and its child nodes are shown as circles in each panel. When every child node has its own unique state (indicated by colors in the circles) [panel **A**], child nodes sharing a state are clustered together under a new mediating node (middle circle of the same color), necessitating only one mutation (lightening bolt of the same color) [panel **B**]. When some child nodes have alternative states (differently colored circles separated by slash) [panel **C**], each such node (thick-rimmed circle) will be clustered differently depending on the assigned state [panel **D**]. A grey circle at the multi-furcated node in panels A and C indicates that the state is unknown yet. An un-numbered circle at the bottom of each genealogy is an outgroup.

A. Input in a simple case.

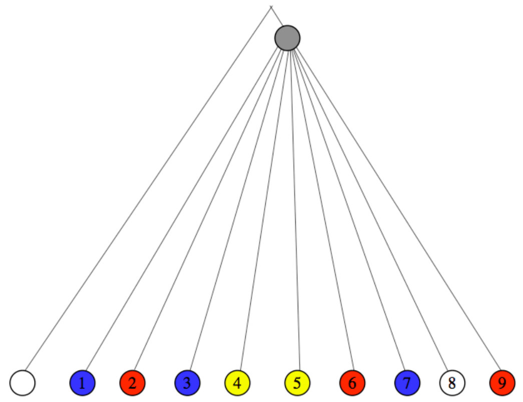

B. Output in the simple case.

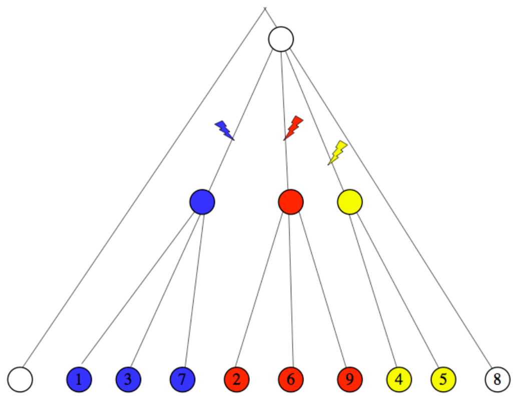

C. Input in a complex case.

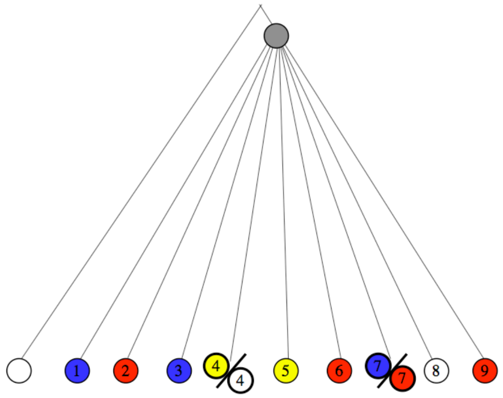

D. Output in the complex case.

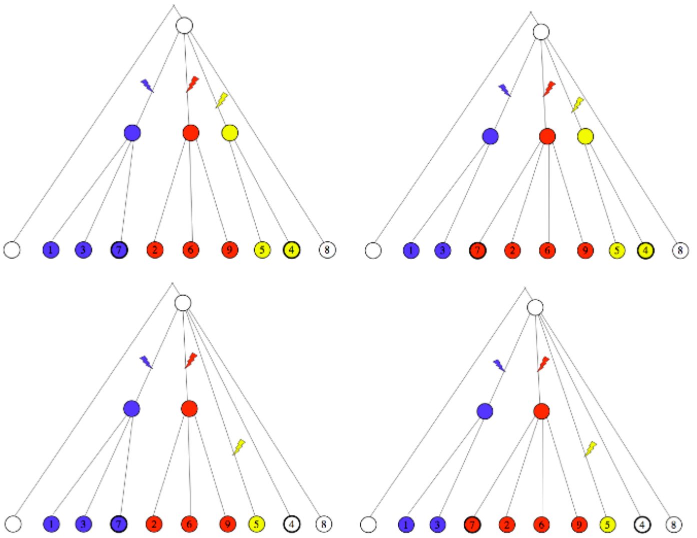

Supplementary figure SS3. Two broad procedures in our new parsimony algorithm.

(A) The input data of our algorithm consists of a (generally incompletely resolved) genealogy of sampled sequences and the states assigned to the sequences. **(B)** The first broad procedure goes on from the exterior nodes up to the top node (i.e. root), calculates the minimum penalty at each interior node on condition that the node has each of the possible states, while inserting additional branches (and nodes) if necessary. **(C)** The second broad procedure goes from the top node down to the exterior nodes, assigning each node (a) state(s) (and (an) additional branch(es) if any) that give(s) the minimum total penalty. Throughout this figure, a two-state model is used for the focal locus. The two states are denoted by black and white circles. A grey circle indicates an unknown state. A Jukes-Cantor type penalty matrix is assumed. A red-rimmed circle and a red segment represent a newly added node and a newly inserted branch, respectively. Dashed arrows indicate the flow of information processing. A red lightening bolt denotes an inferred mutation event. In panel C, the minimum number of mutations under each node is shown in parentheses.

A. Input data.

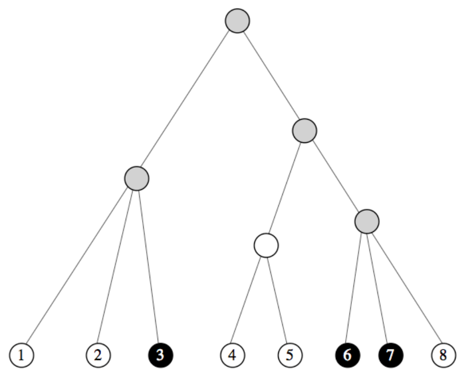

B. Bottom-up procedure.

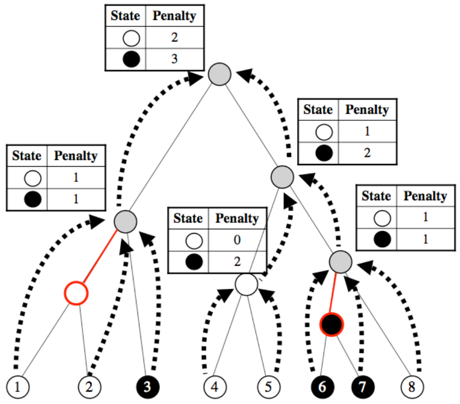

C. Top-down procedure.

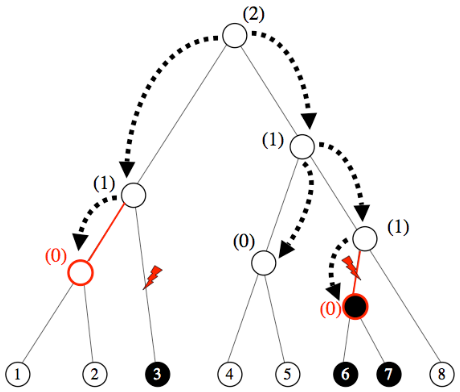

Supplementary figure SS4. Single iteration step of bottom-up procedure at each node (simple cases).

(A) When the subject node is bifurcated, Sankoff's algorithm is applied to calculate the conditional penalties (mini-table near each node). The penalty matrix is shown in the inset around the top-right corner. A two-state model was used for illustration purpose. **(B)** When the subject node is multi-furcated and each of its child nodes has a unique character state (a colored circle), clustering the child nodes sharing the same state will always give the minimum penalty [panel **(C)**]. A red-rimmed circle and a red segment represent a newly added node and a newly inserted branch, respectively, to cluster child nodes sharing a state. A gray circle indicates that the node does not have a state fixed yet.

A. Sankoff's algorithm on bifurcated node.

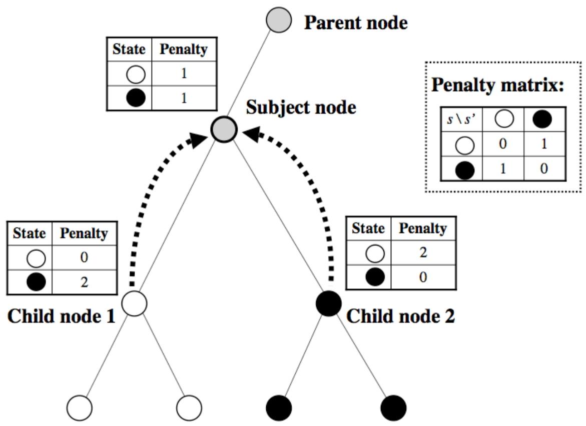

B. Multi-furcated node.

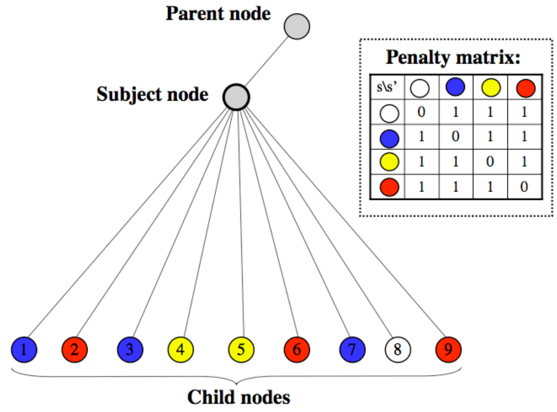

C. Clustering child nodes.

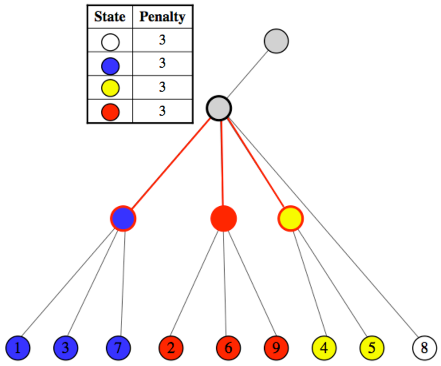

Supplementary figure SS5. Determining the parsimonious genealogical relationships between nodes with different states.

In general, the minimum-penalty conditional on a state at the subject node (white node at the top of each tree) is achieved only under particular genealogies. **(A)** When the penalty matrix is of non-Jukes-Cantor (JC) type, the genealogical relationship among different states becomes important. **(B)** When the matrix is of JC type, the total penalty doesn't depend on the relationship among different states, as long as the nodes sharing the same state cluster together. Thus the star phylogeny will be employed to keep it as flexible as possible. In the genealogy *after* the process in each panel, each lightening bolt and the parenthesized number beside it represent a mutation event and its penalty, respectively.

A. For non-JC type penalty.

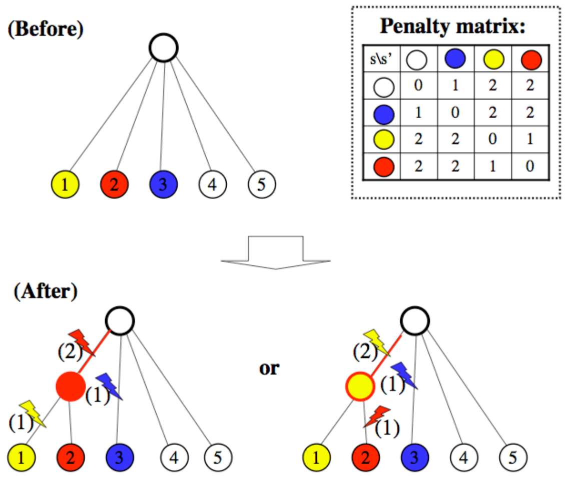

B. For JC type penalty.

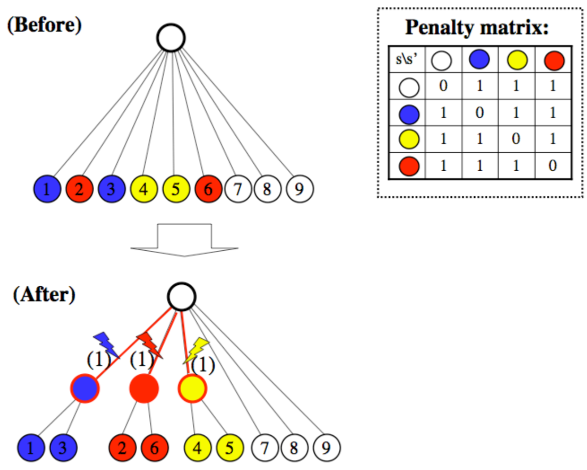

Supplementary figure SS6. Component process of the bottom-up part of the new parsimony algorithm.

The figure diagrams the overall work-flow of the component process at each node of the genealogy. Quoted and double-quoted terms are defined in Supplementary[2] Materials and Methods in this file.

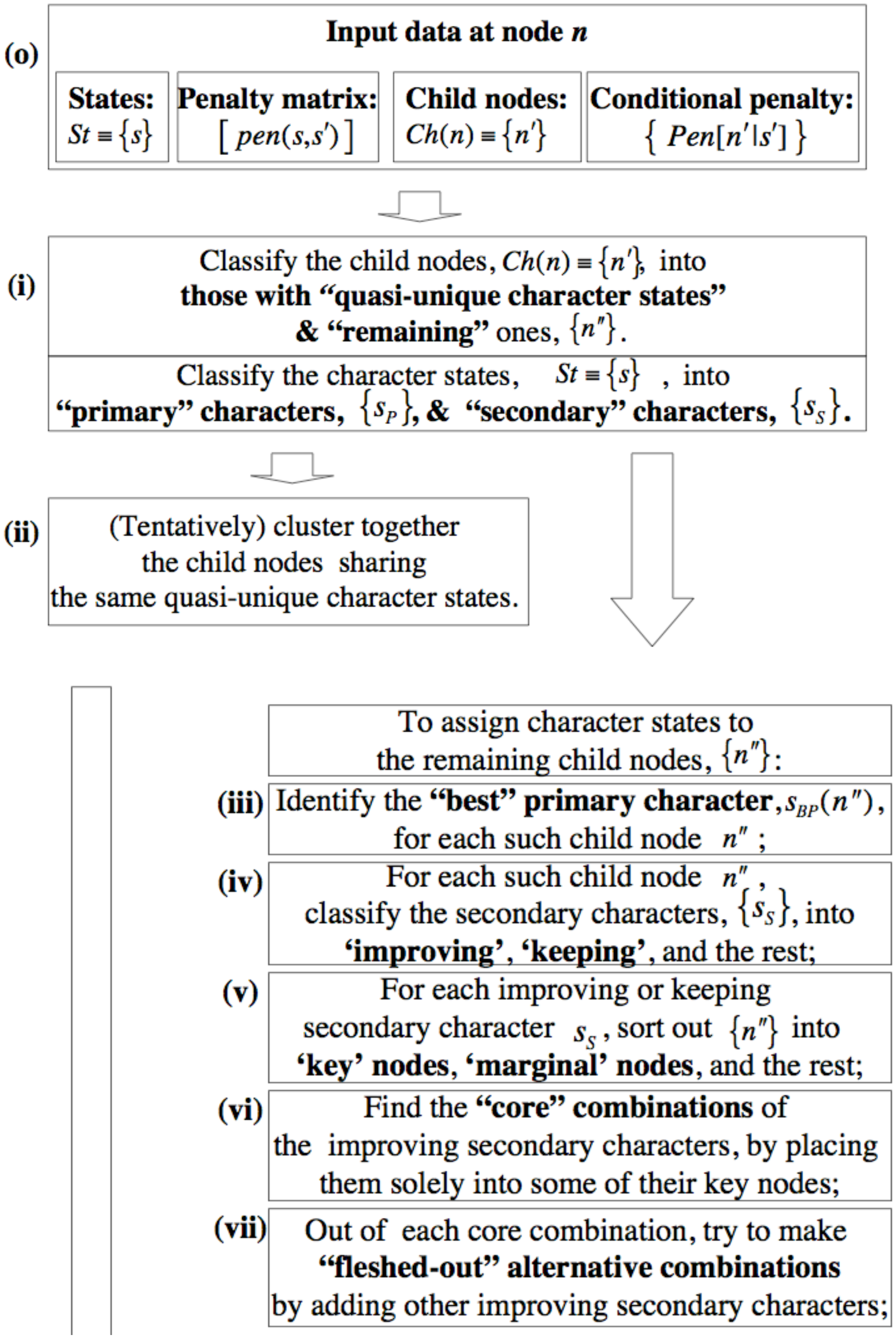

(Supplementary figure SS6)

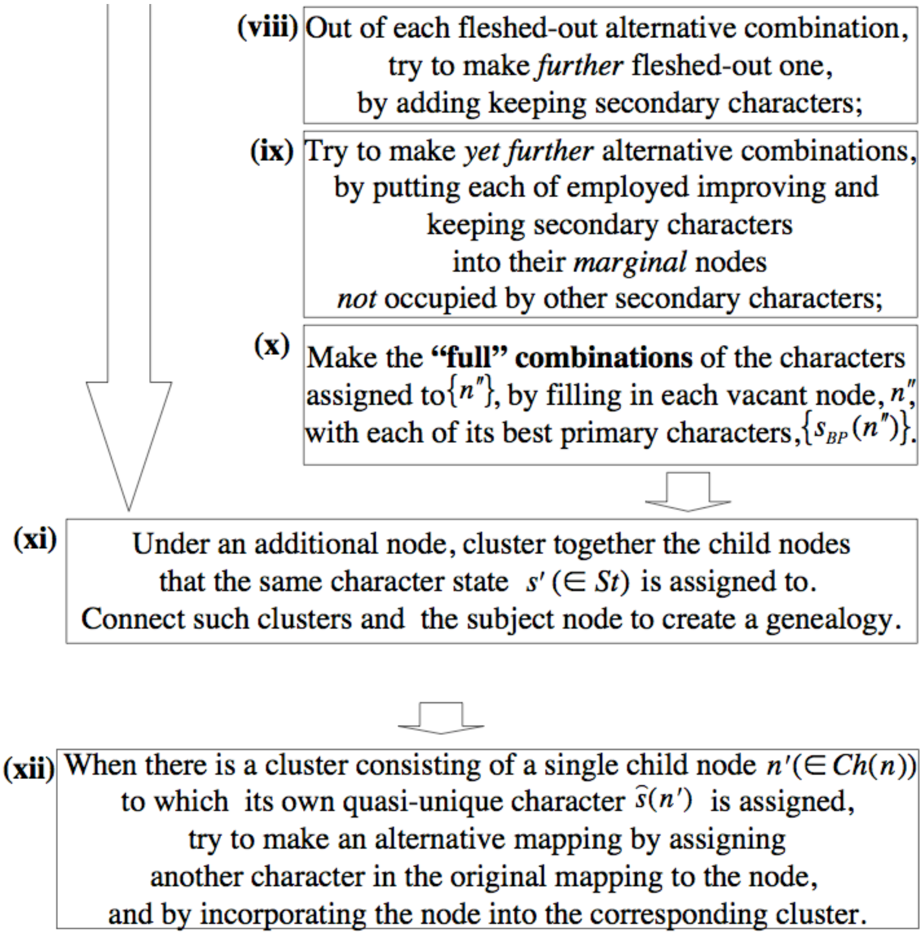

Supplementary figure SS7. Workflow of the core process (step (vi) of Supplementary figure SS6).

Details are described in Supplementary[2] Materials and Methods in this file.

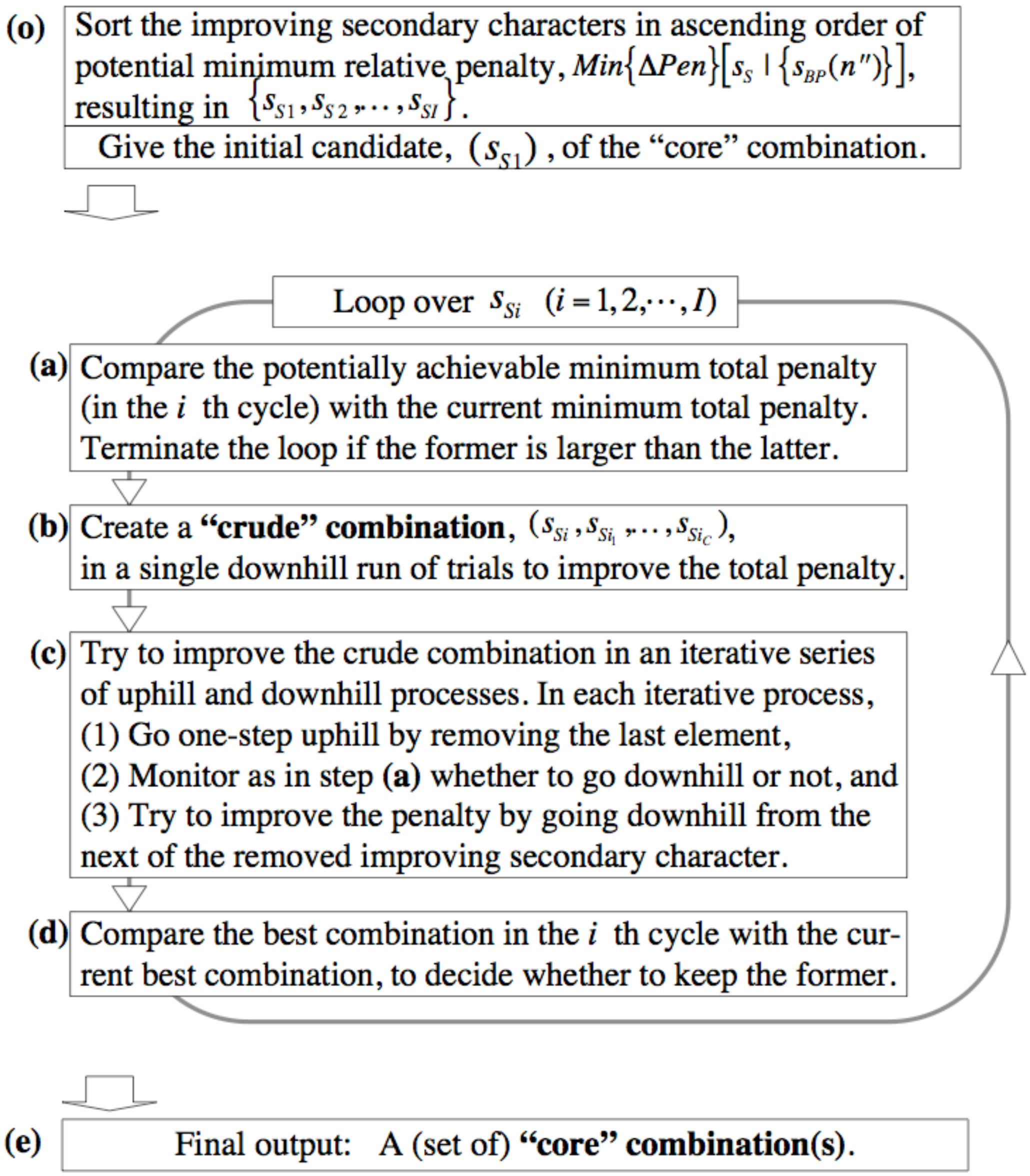

Supplementary figure SS8. Components of the core process (step (vi) of Supplementary figure SS6).

This figure schematically illustrates the component processes of the core process of the bottom-up part of the new parsimony algorithm, which is step (vi) of Supplementary Figure SS6.

NOTATION. In **panel A,** bold-faced figures in the matrices indicate that they will be used in the subsequent processes. In **panel B**, bold-faced letters indicate the improvement (at the node) compared to the last step. Red letters and blue letters indicate that the added character was employed and dismissed, respectively. In **panel C**, a single-lined arrow and a double-lined arrow denote an up-hill step and a downhill step, respectively. Red letters indicate the "current best" combination, and blue letters indicate that the combination is dismissed. (Black thin letters indicate a base-point for a downhill run.)

ABBREVIATIONS: B.P., "best primary" (character); I.S., "improving secondary" (character); K.S., "keeping secondary" (character); R.S., "remaining secondary" (character); Curr_tot_min_penalty, current total minimum penalty; tot_pen, total penalty.

A. Step (o): Sorting the secondary characters and the "remaining" child nodes.

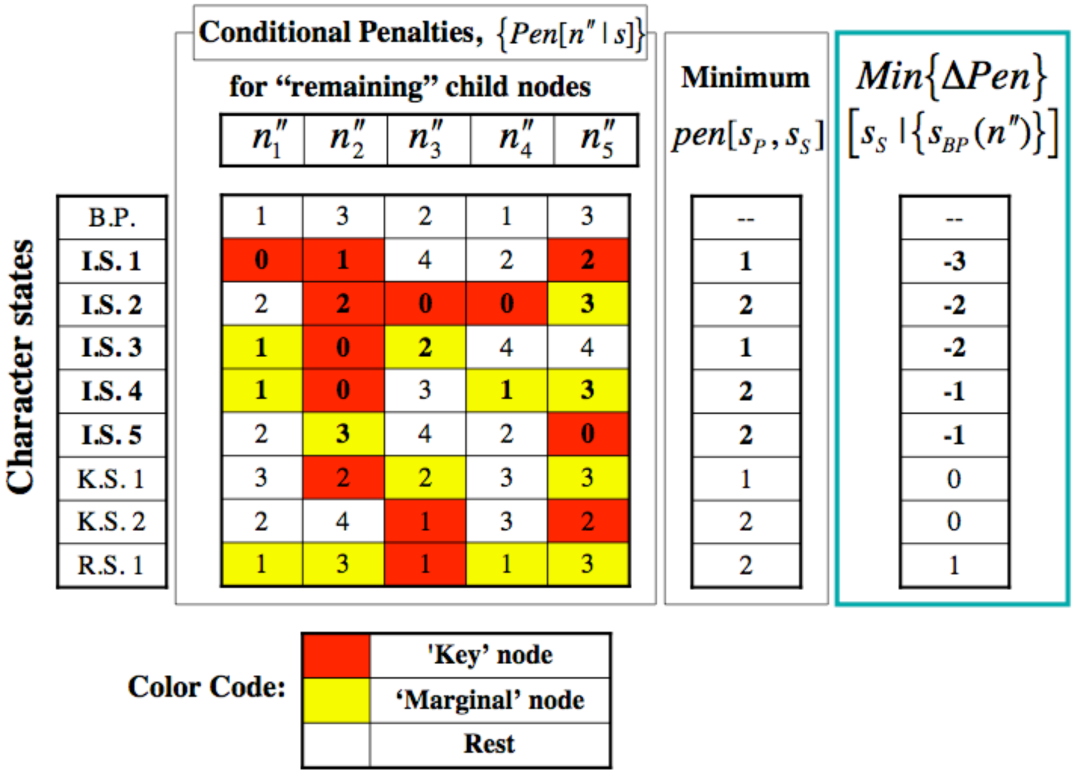

Character states	n''_1	n''_2	n''_3	n''_4	n''_5	Minimum $pen[s_P, s_S]$	$Min\{\Delta Pen\}$ $[s_S \mid \{s_{BP}(n'')\}]$
B.P.	1	3	2	1	3	--	--
**I.S. 1**	**0**	**1**	4	2	**2**	**1**	**-3**
**I.S. 2**	2	**2**	**0**	**0**	**3**	**2**	**-2**
**I.S. 3**	**1**	**0**	**2**	4	4	**1**	**-2**
**I.S. 4**	**1**	**0**	3	**1**	**3**	**2**	**-1**
**I.S. 5**	2	**3**	4	2	**0**	**2**	**-1**
K.S. 1	3	2	2	3	3	1	0
K.S. 2	2	4	1	3	2	2	0
R.S. 1	1	3	1	1	3	2	1

(Supplementary figure SS8)

B. Step (b): A single downhill run of trials to construct a 'crude combination' of characters (in the i = 1st cycle).

Base = ( )

Node	n''_1	n''_2	n''_3	n''_4	n''_5
Character	B.P.	B.P.	B.P.	B.P.	B.P.
$Pen[n''\mid s]$	1	3	2	1	3

$$\sum_{S_S \in \{curr.core\ combi.\}} \underset{s_P:primary}{Min} \{pen(s_P, s_S)\} \;:\; N/A$$

{Curr_tot_ min_penalty} = 10

Initial = (I.S.1)

Node	n''_1	n''_2	n''_3	n''_4	n''_5
Character	**I.S.1**	**I.S.1**	B.P.	B.P.	**I.S.1**
$Pen[n''\mid s]$	**0**	**1**	2	1	**2**

$$\sum_{S_S \in \{curr.core\ combi.\}} \underset{s_P:primary}{Min} \{pen(s_P, s_S)\} = 1$$

{Curr_tot_ min_penalty} = 7
--- employed

Second = (I.S.1, I.S.2)

Node	n''_1	n''_2	n''_3	n''_4	n''_5
Character	I.S.1	I.S.1	**I.S.2**	**I.S.2**	I.S.1
$Pen[n''\mid s]$	0	1	**0**	**0**	2

$$\sum_{S_S \in \{curr.core\ combi.\}} \underset{s_P:primary}{Min} \{pen(s_P, s_S)\} = 3$$

{Curr_tot_ min_penalty} = 6
--- employed

Third = (I.S.1, I.S.2, I.S.3)

Node	n''_1	n''_2	n''_3	n''_4	n''_5
Character	I.S.1	**I.S.3**	I.S.2	I.S.2	I.S.1
$Pen[n''\mid s]$	0	**0**	0	0	2

$$\sum_{S_S \in \{curr.core\ combi.\}} \underset{s_P:primary}{Min} \{pen(s_P, s_S)\} = 4$$

{Curr_tot_ min_penalty} = 6
--- dismissed

Fourth = (I.S.1, I.S.2, I.S.4)

Node	n''_1	n''_2	n''_3	n''_4	n''_5
Character	I.S.1	**I.S.4**	I.S.2	I.S.2	I.S.1
$Pen[n''\mid s]$	0	**0**	0	0	2

$$\sum_{S_S \in \{curr.core\ combi.\}} \underset{s_P:primary}{Min} \{pen(s_P, s_S)\} = 5$$

{Curr_tot_ min_penalty} = 7
--- dismissed

Fifth = (I.S.1, I.S.2, I.S.5)

Node	n''_1	n''_2	n''_3	n''_4	n''_5
Character	I.S.1	I.S.1	I.S.2	I.S.2	**I.S.5**
$Pen[n''\mid s]$	0	1	0	0	**0**

$$\sum_{S_S \in \{curr.core\ combi.\}} \underset{s_P:primary}{Min} \{pen(s_P, s_S)\} = 5$$

{Curr_tot_ min_penalty} = 6
--- dismissed

∴

{The final "crude combination" in the cycle with i = 1} = (I.S.1, I.S.2)

Node	n''_1	n''_2	n''_3	n''_4	n''_5
Character	**I.S.1**	**I.S.1**	**I.S.2**	**I.S.2**	**I.S.1**
$Pen[n''\mid s]$	**0**	**1**	**0**	**0**	**2**

$$\sum_{S_S \in \{curr.core\ combi.\}} \underset{s_P:primary}{Min} \{pen(s_P, s_S)\} = 3$$

{Curr_tot_ min_penalty} = 6

Similarly,

{The final "crude combination" in the cycle with i = 2} = (I.S.2, I.S.3, I.S.5)

Node	n''_1	n''_2	n''_3	n''_4	n''_5
Character	B.P.	**I.S.3**	**I.S.2**	**I.S.2**	**I.S.5**
$Pen[n''\mid s]$	1	**0**	**0**	**0**	**0**

$$\sum_{S_S \in \{curr.core\ combi.\}} \underset{s_P:primary}{Min} \{pen(s_P, s_S)\} = 5$$

{Curr_tot_ min_penalty} = 6

* No "crude combinations" with {Curr_tot_min_penalty} ≤ 6 in the cycles with $i = 3, 4, 5$.

(Supplementary figure SS8)

C. Step (c): Up-and-down trials to improve the minimum total penalty.

In the cycle with i = 1, start from "Crude" = (I.S.1, I.S.2) with *tot_pen* = 6.

=> (I.S.1) with *tot_pen* = 7 -> **(I.S.1, I.S.3) with *tot_pen* = 7**

-> **(I.S.1, I.S.4) with *tot_pen* = 8** -> **(I.S.1, I.S.5) with *tot_pen* = 7**

-> (I.S.1) with *tot_pen* = 7 => ( ) ... **END.**

∴ **The "best combination" in the cycle = (I.S.1, I.S.2) with *tot_pen* = 6.**

In the cycle with i = 2, start from "Crude" = (I.S.2, I.S.3, I.S.5) with *tot_pen* = 6.

=> (I.S.2, I.S.3) with *tot_pen* = 7 => (I.S.2) with *tot_pen* = 8

-> **(I.S.2, I.S.4) with *tot_pen* = 7** -> **(I.S.2, I.S.4, I.S.5) with *tot_pen* = 7**

-> **(I.S.2, I.S.4) with *tot_pen* = 7** => (I.S.2) with *tot_pen* = 8

-> **(I.S.2, I.S.5) with *tot_pen* = 7** -> (I.S.2) with *tot_pen* = 8

=> ( ) **... END.**

∴ **The "best combination" in the cycle = (I.S.2, I.S.3, I.S.5) with *tot_pen* = 6.**

Supplementary figure SS9. Trying to "flesh-out" a "core combination" of secondary characters (step (vi) of Supplementary figure SS6).

This figure schematically illustrates attempts to "flesh-out" a "core combination", (I.S.1, I.S.2), which was obtained in Supplementary Figure SS8. Accordingly, the input setting in panel A of Supplementary Figure SS7 is assumed also here. The figure only illustrates the successful attempts to "flesh-out" the "core" (I.S.1, I.S.2). Red letters indicate successfully added secondary characters. At the bottom is a list of the resulting "fleshed-out" combinations.

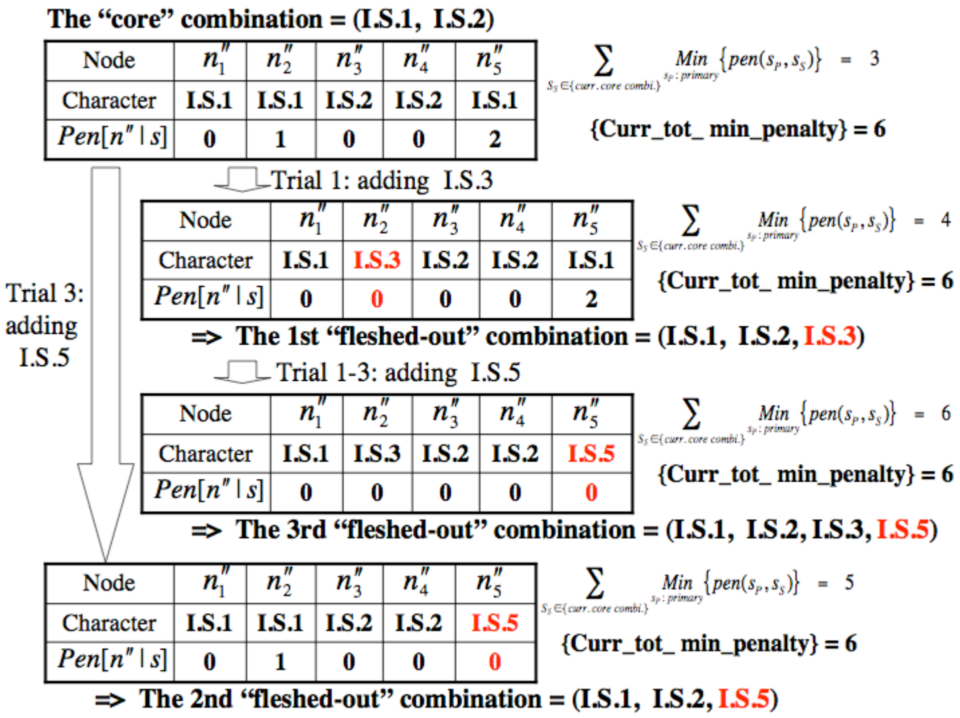

∴

The set of "fleshed-out" combinations (including the original "core") of improving secondary characters:

(I.S.1, I.S.2); (I.S.1, I.S.2, I.S.3); (I.S.1, I.S.2, I.S.5); (I.S.1, I.S.2, I.S.3, I.S.5).

Supplementary figure SS10. Constructing the genealogy of a subject node and its child nodes according to the assigned character states (step xi of Supplementary figure SS6). **(A)** Input data for this step consists of a subject node (thick-rimmed circle at the top), its children (numbered nodes at the bottom), characters assigned to the nodes (colors in the circle), and a penalty matrix. Characters are classified into primary and secondary ones. **(B)** As an interim step, child nodes are clustered according to the characters assigned. **(C)** Finally, genealogical relationships among the clusters (i.e. among the characters) are determined so as to minimize the total penalty. A red segment and a lightening bolt on it represent a newly inserted branch and a mutation event along it, respectively.

A. Input data.

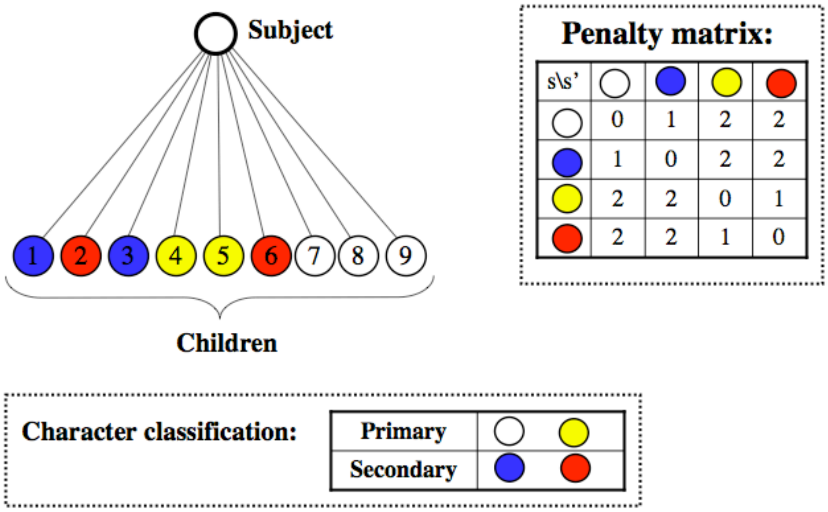

B. Interim step.

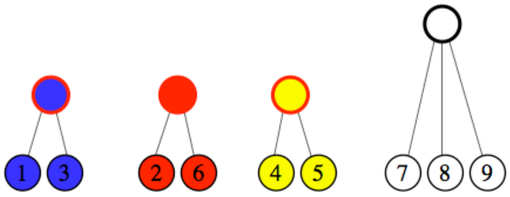

C. Output data.

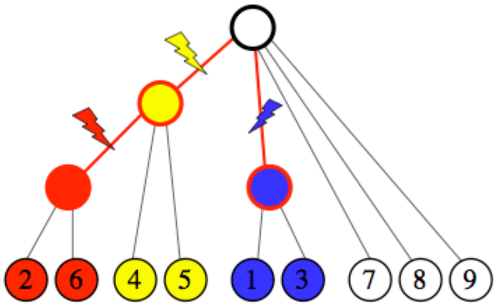

Supplementary figure SS11. Final expansion of the set of alternative parsimonious scenarios (step xii of Supplementary figure SS6).

(A) Input data *of the whole process in Supplementary Figure SS6*, at node n (thick-rimmed circle at the top of the unresolved genealogy) and a state assigned to it (white). Numbered circles at the bottom of the unresolved genealogy represent child nodes. Different colors represent different character states. Child node 4 (n'_4) is thick-rimmed, because it is tentatively occupied by its own quasi-unique character (yellow) and is the focus of attention. **(B)** The parsimonious scenario resulting from the states assigned in the unresolved genealogy in panel A. Note that there is no node mediating nodes n and n'_4. **(C)** Alternative parsimonious scenarios created by assigning different states to child node 4. A parenthesized number below child node 4 (n'_4) is the penalty conditional on its state. A lightening bolt and an accompanying parenthesized number denote a mutation and the penalty it incurs, respectively.

A. Input (of the whole process in Supplementary Figure SS6).

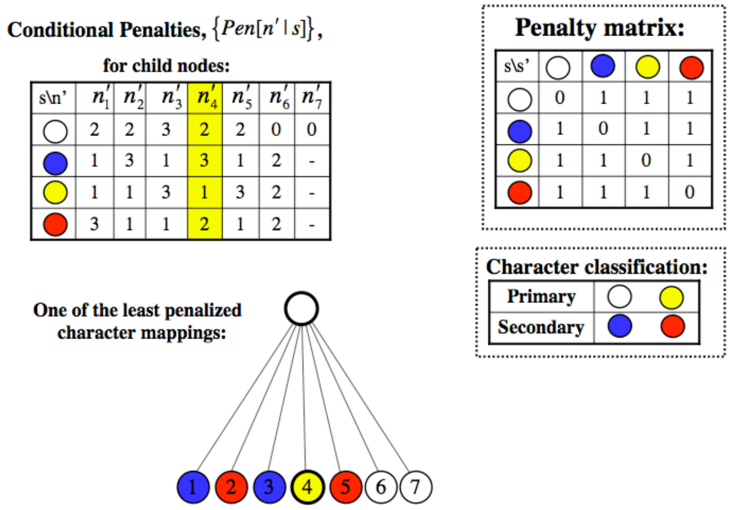

B. Resulting parsimonious scenario.

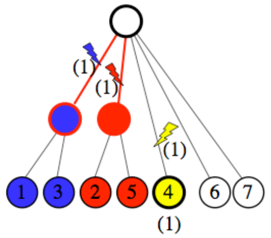

C. Alternative parsimonious scenarios.

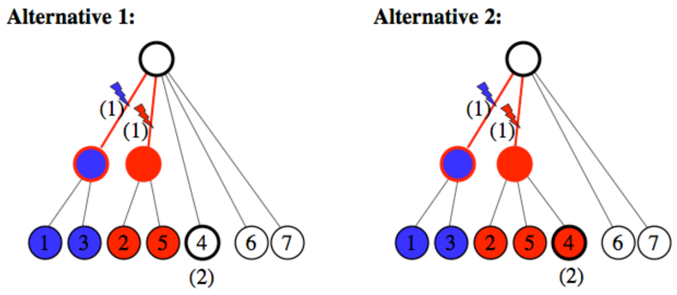

Supplementary figure SS12. Processes of the top-down procedure at each node.

(A) To the top node (thick-rimmed circle at the top), states with the smallest total penalty are assigned, just as in Sankoff's (1975) algorithm. **(B)** To each child (numbered circle) of a bifurcated node (circle labeled 'P'), assign a state giving the smallest penalty conditional on the state of the node 'P', just as in Sankoff's (1975) algorithm. **(C)** For a multi-furcated node (circle labeled 'P'), parsimonious scenarios down to its children (numbered circles), conditional on its states, are already enumerated in the bottom-up part of the new algorithm (in the dashed box at the top). Therefore, once the state of node 'P' is specified in the previous step of the top-down part, the states are automatically assigned to the child nodes, and their genealogical relationships are automatically resolved, as a part of each fixed parsimonious scenario.

A. For the top node.

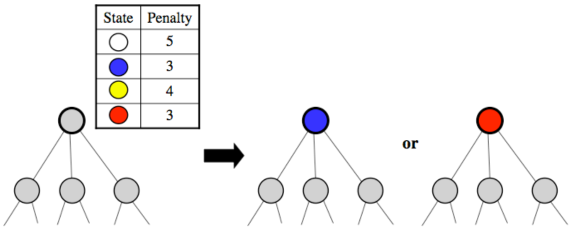

B. For children of a bifurcated node.

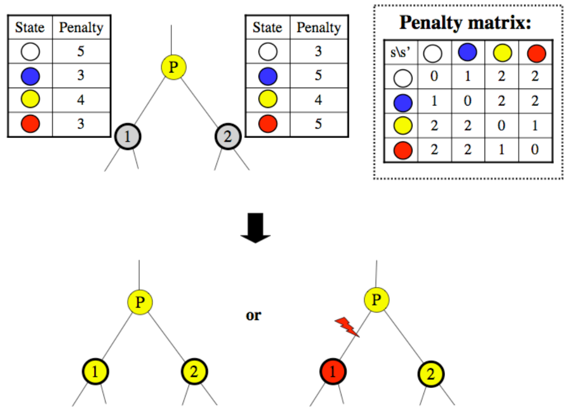

C. For children of a multi-furcated node.

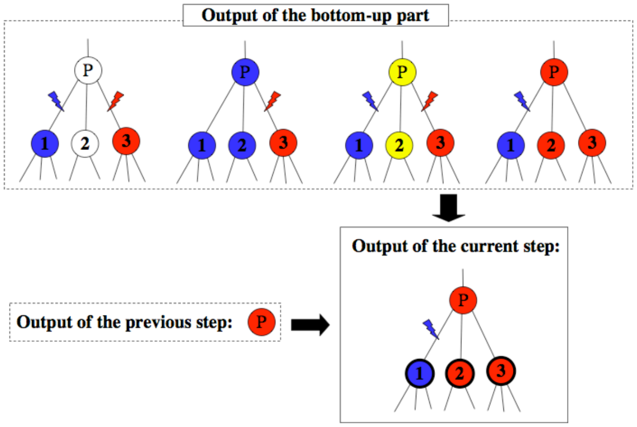

Supplementary figure SS13. Improved output format of the new parsimony algorithm.

(A) In old versions, each parsimonious scenario was represented by a set of states (colored circles) assigned to the (default and newly added) interior nodes (left genealogy). To save the storage space, in the new version, each parsimonious scenario is represented by a set of mutations (lightening bolts) along the (default and newly added) branches. **(B)** In the new version, parsimonious scenarios are lumped together if they share the same mutations along the branches immediately under the bifurcated nodes (thick-edged lightening bolts). **(C)** Once lumped together, such scenarios can be factorized into some components, one consisting of mutations on the branches under bifurcated nodes (thick-edged lightening bolts in the genealogy with a grey-shaded thick-dotted box), and each of the others consisting of mutations somewhere from a multi-furcated node to its children in the input genealogy (thin-edged lightening bolts in each thick dotted box within braces).

A. Recording state changes on branches.

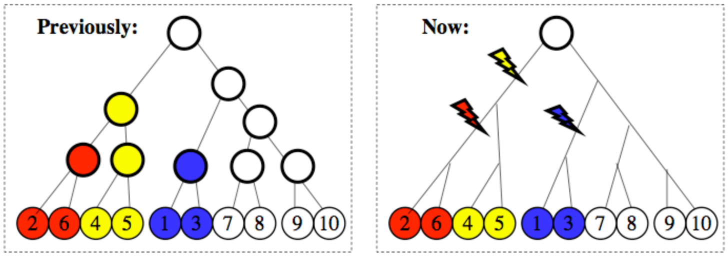

(Supplementary figure SS13)

B. Lumping together parsimonious scenarios.

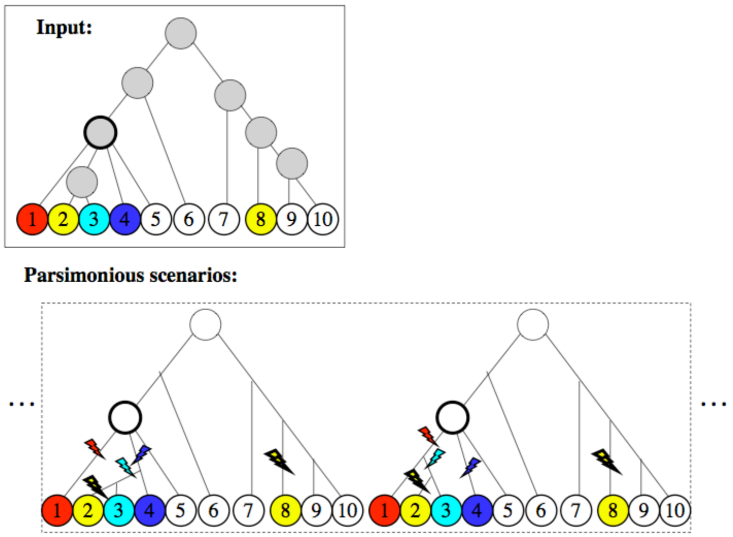

C. Factorizing the sets of scenarios.

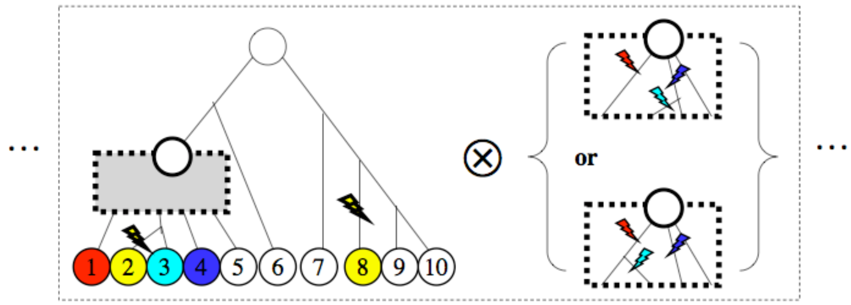

Supplement: Additional file 2 — Detailed descriptions of our new parsimony algorithm to enumerate parsimonious mutation scenarios on an incompletely resolved genealogy. [file 1471-2156-14-37-S2.pdf]
